# Asymmetric Cation‐Olefin Monocyclization by Engineered Squalene–Hopene Cyclases

**DOI:** 10.1002/anie.202108037

**Published:** 2021-09-17

**Authors:** Michael Eichenberger, Sean Hüppi, David Patsch, Natalie Aeberli, Raphael Berweger, Sandro Dossenbach, Eric Eichhorn, Felix Flachsmann, Lucas Hortencio, Francis Voirol, Sabine Vollenweider, Uwe T. Bornscheuer, Rebecca Buller

**Affiliations:** ^1^ Zurich University of Applied Sciences Life Sciences and Facility Management Einsiedlerstrasse 31 8820 Wädenswil Switzerland; ^2^ Department of Biotechnology Delft University of Technology Van der Maasweg 9 2629 HZ Delft The Netherlands; ^3^ Institute of Biochemistry, Dept. of Biotechnology & Enzyme Catalysis Greifswald University Felix-Hausdorff-Strasse 4 17487 Greifswald Germany; ^4^ Fragrances S&T Ingredients Research Givaudan Schweiz AG Kemptpark 50 8310 Kemptthal Switzerland; ^5^ Science & Technology Givaudan International SA Kemptpark 50 8310 Kemptthal Switzerland

**Keywords:** chemoenzymatic synthesis, cyclization, protein engineering, squalene–hopene cyclases, substrate engineering

## Abstract

Squalene–hopene cyclases (SHCs) have great potential for the industrial synthesis of enantiopure cyclic terpenoids. A limitation of SHC catalysis has been the enzymes’ strict (S)‐enantioselectivity at the stereocenter formed after the first cyclization step. To gain enantio‐complementary access to valuable monocyclic terpenoids, an SHC‐wild‐type library including 18 novel homologs was set up. A previously not described SHC (*Aci*SHC) was found to synthesize small amounts of monocyclic (R)‐γ‐dihydroionone from (E/Z)‐geranylacetone. Using enzyme and process optimization, the conversion to the desired product was increased to 79 %. Notably, analyzed *Aci*SHC variants could finely differentiate between the geometric geranylacetone isomers: While the (Z)‐isomer yielded the desired monocyclic (R)‐γ‐dihydroionone (>99 % *ee*), the (E)‐isomer was converted to the (S,S)‐bicyclic ether (>95 % *ee*). Applying the knowledge gained from the observed stereodivergent and enantioselective transformations to an additional SHC‐substrate pair, access to the complementary (S)‐γ‐dihydroionone (>99.9 % *ee*) could be obtained.

## Graphical abstract

The intrinsic capability of squalene–hopene cyclases to convert (*E*)‐ or (*Z*)‐substrates enantiospecifically to (*S*)‐ or (*R*)‐configurated monocyclic terpenoids was put into action by a combination of enzyme evolution and substrate engineering for the synthesis of (*S*)‐ and (*R*)‐γ‐dihydroionone with >99 % *ee*.

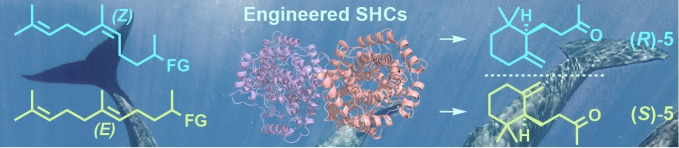

## Introduction

**Scheme 1:**
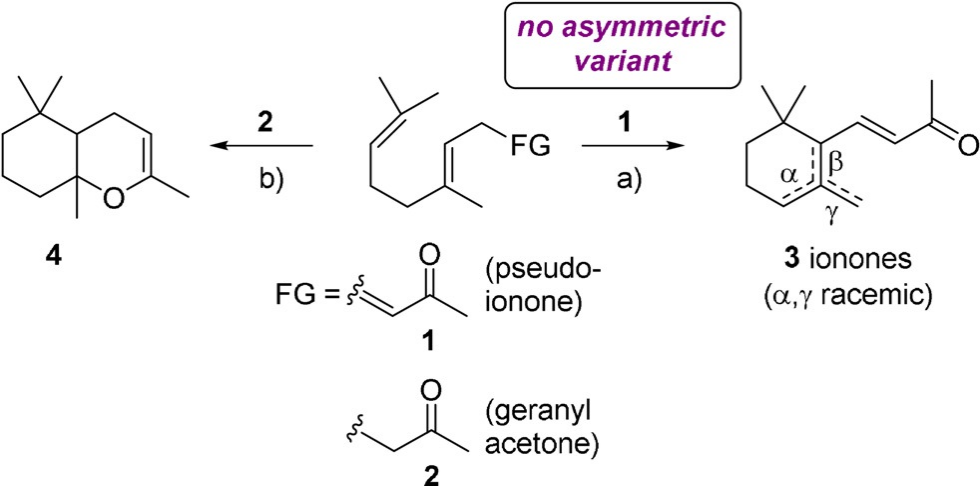
Cation‐olefin cyclizations of pseudoionone (**1**) and geranylacetone (**2**) to racemic ionones (**3**) and bicyclic enolether (**4**), respectively. a) Brønstedt acid b) Brønstedt acid or terpene cyclase.

Ionones are significant contributors to the appealing scents of many flowers and fruits, including violets, roses, or raspberries.^[1]^ They belong to a family of natural products known as apocarotenoids, which are derived from carotenoids by oxidative cleavage catalyzed by carotenoid oxygenases.^[2]^ An efficient synthetic access to racemic ionones by cation‐olefin cyclization of pseudoionone (**1**) was discovered already in the late 19^th^ century by Tiemann and Krüger (Scheme 1).^[3]^ Accordingly, ionones were among the first commercially utilized synthetic fragrance ingredients, featured for example in the iconic fragrance *Vera Violetta* (Roger & Gallet, 1893).

**Scheme 2:**
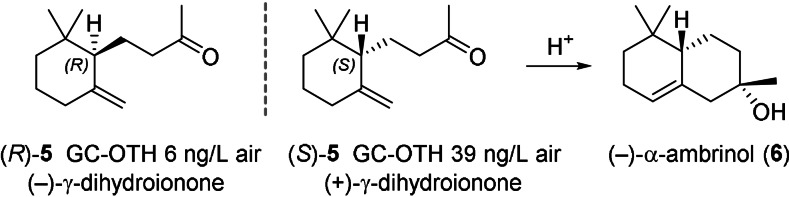
GC odour thresholds (GC‐OTH) of γ‐dihydroionone enantiomers (**5**) and conversion of (*S*)‐**5** to (−)‐α‐ambrinol (**6**).

The organoleptically strongest ionones are the achiral β‐ionone and the (*S*)‐(+)‐isomer of γ‐ionone, which has an over 150x lower perception threshold than its optical antipode.^[4]^ Interestingly, this trend is inverted for the corresponding γ‐dihydro‐analogue, for which the non‐natural (*R*)‐(−)‐isomer has a 6‐fold lower perception threshold compared to the (*S*)‐(+)‐isomer (Scheme 2).^[5]^ The natural (*S*)‐(+)‐γ‐dihydroionone ((*S*)‐**5**) occurs for example in Ambergris and is of interest as an intermediate for the synthesis of (−)‐α‐ambrinol (**6**), which exhibits a highly appreciated animalic scent typical for aged Ambergris tincture (Scheme 2).

The interest in optically active ionones, including both enantiomers of γ‐dihydroionone (**5**), has stimulated the development of numerous methods for their synthesis.^[5]^ However, the most obvious synthesis route toward these compounds is currently missing, namely the asymmetric cation‐olefin cyclization of pseudoionone (**1**) or a suitable derivative thereof. It appears that carbocation formation at the unpolar isoprene end of the linear chain in combination with enantiospecific folding of the linear C_13_ precursor to form a monocycle is difficult to achieve with classical asymmetric catalysis.

**Scheme 3:**
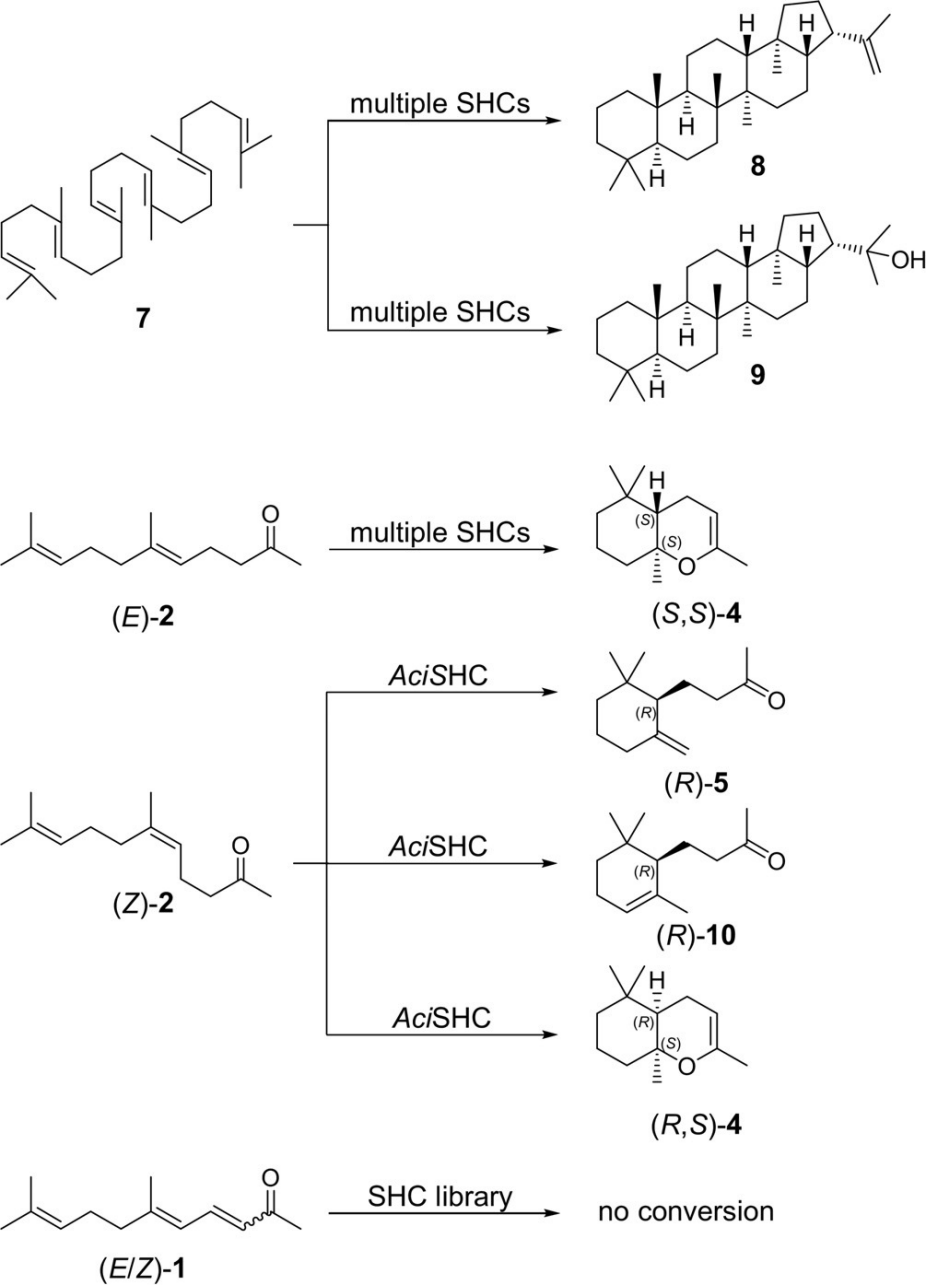
Transformations observed in the screening of the SHC library with substrates **1**, **2** and **7**.

In contrast, squalene–hopene cyclases (SHCs), which belong to the class II terpene cyclases, are capable of locking linear terpenoid substrates in defined chiral conformations, which allows to achieve polyene cyclizations with perfect stereocontrol. Consequently, SHCs have great potential as industrial biocatalysts for the production of enantiopure cyclic terpenoids. A widely spread model reaction is the cyclization of the linear C_30_ triterpene squalene (**7**) into the pentacyclic products hopene (**8**) and hopanol (**9**), through the generation of five new C−C bonds and nine new stereocenters (Scheme 3).^[6]^ The reaction is initiated by the protonation of the unactivated terminal isoprene unit with the unusually acidic middle aspartate of the DXDD active site motif. The excellent chemo‐, regio‐, and stereocontrol over the polycyclization cascade is achieved through pre‐folding of the substrate in a product‐like conformation, stabilization and shielding of the highly reactive carbocation intermediates from side reactions, and a selective termination through base assisted proton elimination or addition of water.[^7^, ^8^] Terpene cyclases from the SHC family are promiscuous enzymes and accept molecules ranging from C_10_ monoterpenoids^[9]^ to C_35_ squalene analogues,^[10]^ and the cyclization reaction can be initiated through protonation of unactivated olefins, carbonyls, and epoxides.^[11]^ This is in contrast to other main families of class II terpene cyclases: oxidosqualene cyclases are limited to substrates containing an epoxide functional group for initial protonation,^[12]^ while class II diterpene cyclases such as *ent*‐copalyl diphosphate synthases are generally only active towards the diphosphate containing substrate geranylgeranylpyrophosphate.^[13]^

Importantly, SHCs have proven to be highly evolvable: Engineered SHC variants with not more than three mutations enabled a viable industrial‐scale process to obtain Ambrofix^TM^,[^14^, ^15^] as well as dramatically increased activity and altered chemo‐ and stereoselectivity of cyclization reactions with mono‐ and sesquiterpenoids, such as geraniol,^[11]^ farnesol,^[16]^ or citronellal.^[17]^ A limitation of SHCs, however, is their strict (*S*)‐enantioselectivity at the stereocenter formed after the first cyclization of all polyisoprenoids tested so far (an overview of products is given in reviews[^18^, ^19^]).

Here, we report our efforts to gain enantio‐complementary access to valuable monocyclic terpenoids such as (*R*)‐ and (*S*)‐γ‐dihydroionone (**5**) via SHC catalysis. Even though the natural diversity of SHC sequences is vast,[^20^, ^21^] most of the work on non‐native substrates has thus far focused on two enzyme variants from *Alicyclobacillus acidocaldarius* (*Aac*SHC) and *Zymomonas mobilis* (*Zmo*SHC1)^[19]^ and only one study reported a screening panel consisting of 12 wild‐type enzymes.^[22]^ Thus, to identify enzymes capable of synthesizing (*R*)‐ and (*S*)‐γ‐dihydroionone (**5**), we opted for a screening approach based on an SHC wild‐type library, which included 18 novel SHC homologs. Building on the ability of a newly identified SHC from *Acidothermus cellulolyticus* to generate the monocyclic (*R*)‐γ‐dihydroionone ((*R*)‐**5**), we optimized the enzyme by directed evolution and could improve the conversion of nerylacetone ((*Z*)‐**2**) to (*R*)‐γ‐dihydroionone ((*R*)‐**5**) by two orders of magnitude to 79 % in 48 h. It should be noted, that during the preparation of this manuscript, a study by the Hauer group was published, which similarly reports the biocatalytic production of (*R*)‐γ‐dihydroionone ((*R*)‐**5**) by an engineered SHC from *Alicyclobacillus acidocaldarius*. After five rounds of directed evolution, the authors identified an *Aac*SHC variant with four mutations, which exhibited excellent selectivity (99.5 % *ee*) and conversion (89 %) in seven days.^[23]^

In our report, we thus confirm the exciting observation that it is possible to obtain (*R*)‐selective monocyclizations via SHC biocatalysis (>99 % *ee*) yet using the distinct *Aci*SHC enzyme (51.6 % sequence identity to *Aac*SHC). In addition, we observed that all of our *Aci*SHC variants exhibited exquisite selectivity in the transformation of the geometric geranylacetone (**2**) isomers: While the (*Z*)‐isomer yielded the desired monocyclic (*R*)‐**5** product, the (*E*)‐isomer led to the formation of the bicyclic enolether (*S*,*S*)‐**4**. Biochemical and docking studies helped us to understand the mechanistic basis of the observed sterodivergent and enantioselective cyclization reactions. Harnessing this knowledge, we ultimately succeeded to additionally obtain the enantio‐complementary (*S*)‐**5** (>99.9 % *ee*) through the application of an appropriately chosen SHC‐substrate pair.

## Results and Discussion

In our quest to create an efficient biocatalyst for the enantioselective production of (dihydro‐)ionones, we aimed to identify an SHC enzyme with the capability to generate monocyclic products from either (*E*/*Z*)‐geranylacetone (**2**) or (*E*/*Z*)‐pseudoionone (**1**). *Aac*SHC,^[24]^ *Zmo*SHC1,^[24]^ and engineered variants of these enzymes^[25]^ were previously reported to be inactive towards **1** and were found to convert **2** exclusively into the bicyclic product **4**. Consequently, we chose to explore the SHC diversity beyond these heavily studied variants by setting up a comprehensive screening panel of 31 wild‐type enzymes, selected to span all major clades of the phylogenetic tree (Figure S1). The screening library consisted of 13 previously characterized class II terpene cyclases from the SHC‐family and 18 novel SHC homologs, which were identified through the presence of two defining PFAM domains for type II triterpene cyclases (PF13249, PF13243) and the SHC‐family specific DXDD active site motif (Table S2). As thermostable enzyme scaffolds can be superior starting points for protein engineering and directed evolution approaches,^[26]^ ten of the novel sequences were explicitly chosen to originate from thermophilic bacteria.

**Figure 1:**
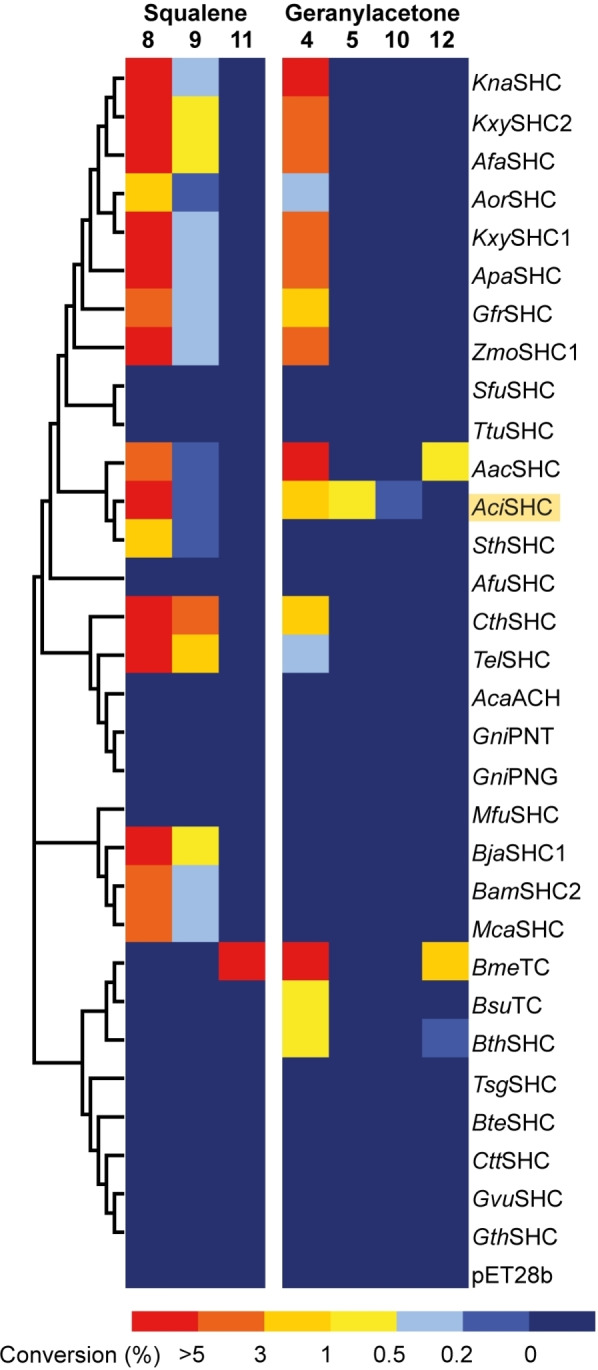
Characterization of the wild‐type SHC library with respect to the enzymes’ activity towards squalene (**7**) and (*E*/*Z*)‐geranylacetone (**2**). Whole‐cell biotransformations were carried out by supplementing cell lysate with 10 mM substrate in 50 mM citrate buffer at pH 6 containing 0.8 % (**7**) or 0.2 % (**2**) of Triton‐X‐100. The SHCs are ordered based on phylogenetic relationship. Highlighted in yellow is *Aci*SHC, the only wild‐type SHC converting **2** into monocyclic products **5** and **10**. Products **11** and **12** could not be structurally assigned.

To characterize our SHC library and evaluate the biocatalysts’ potential for (dihydro)ionone production, we overexpressed the enzymes in *E. coli* BL21(DE3) and carried out whole‐cell biotransformations with 10 mM squalene (**7**), 10 mM (*E*/*Z*)‐geranylacetone (**2**), and 10 mM (*E*/*Z*)‐pseudoionone (**1**). Product formation was analyzed using gas chromatography coupled to mass spectrometry equipped with a flame ionization detector (GC‐MS‐FID) (Figure 1). Nineteen of the investigated SHCs showed activity towards at least one substrate. Notably, ten of the active enzymes correspond to novel SHC homologs, with sequence identities to experimentally characterized variants between 52.6 % and 82.9 %. These results validate our bioinformatic search strategy, and the new enzymes further expand the toolbox of available SHCs for biocatalysis.

While (*E*/*Z*)‐geranylacetone (**2**) was converted by 15 members of our SHC panel (Figure 1), our screen did not identify any SHC homologs with activity towards pseudoionone (**1**), possibly due to steric and/or electronic effects of the conjugated γ,δ‐double bond of **1**, which is the distinguishing feature from **2** (Figure S2). Analyzing the (*E*/*Z*)‐geranylacetone (**2**) conversion data in more detail, we identified *Aci*SHC, a novel SHC homolog from the thermophilic bacterium *Acidothermus cellulolyticus*, as a possible candidate for further development. While conversion of the C_13_ substrate **2** into the bicyclic enol ether (**4**) was widespread among the SHC panel, *Aci*SHC was the only enzyme included in the panel that generated two additional minor products with conversions of 0.7 % and 0.05 %, respectively. These were identified as γ‐dihydroionone (**5**) and α‐dihydroionone (**10**) by GC‐MS through comparison with authentic reference materials.

Intrigued by these results, we created a sequence alignment of the active pocket^[20]^ of the 14 SHCs, which mainly convert (*E*/*Z*)‐geranylacetone (**2**) into the bicyclic product **4** and, in three cases, the structurally unassigned product **12** and compared it to the amino acid distribution of *Aci*SHC (Figure S3). Surprisingly, the sequence alignment revealed that the active site of *Aci*SHC appears to be similar in construction as those of the remaining enzyme panel: Of the 36 residues lining the substrate‐binding pocket, only I41, located more than 18 Å away from the catalytic acid D380, was found to be unique in *Aci*SHC (Figure S3). Thus, we proceeded to investigate the unusual product selectivity of *Aci*SHC by constructing its homology model based on the crystal structure of *Aac*SHC (PDB ID: 1SQC; identity: 51.62 %; similarity: 0.45) using SWISS‐MODEL^[27]^ followed by docking studies of (*E*)‐**2** and (*Z*)‐**2** using the software tool AutoDock Vina.^[28]^ Both substrate stereoisomers afforded a docking state with a productive *pre*‐chair conformation for monocyclization, however, no “all” *pre*‐chair state as required for the formation of the bicyclic product was found (Figure S4).

Thus, even though the identified substrate poses did not fully explain the experimentally observed product distribution, our docking results led us to speculate that already slight changes in the active pocket geometry might result in alternative pre‐folding states of **2**. In this way, the enzyme could channel the substrate either into a cationic cascade necessary for the formation of the bicyclic enol ether (**4**) or allow termination of the reaction after a single ring‐forming event to yield **5**. In the latter case, deprotonation of the exocyclic methylene group could occur through D378, which in our model of *Aci*SHC is situated at a distance of 2.6 Å from the hydrogen of the relevant carbon C‐11. The presence of D378, acting as a catalytic base, could explain the unexpected selectivity for the formation of the energetically unfavorable exocyclic deprotonation product **5** over **10** (Figure S5).

**Figure 2:**
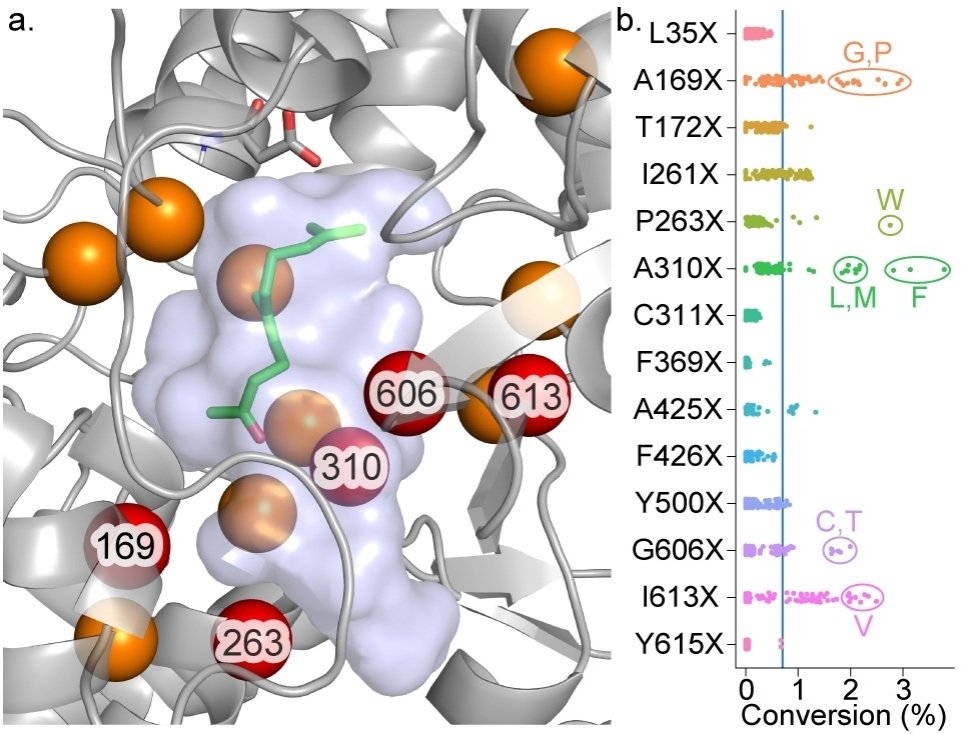
a) Homology model of *Aci*SHC with (*Z*)‐**2** docked in the active pocket. Sites colored in orange/red were targeted for single‐site saturation mutagenesis, the top‐performing sites (red) were then selected for combinatorial mutagenesis in a second evolution round. b) Conversion of (*E*/*Z*)‐geranylacetone (**2**) to γ‐dihydroionone (**5**) (%FID) by the SHC variants generated in the first round of evolution. The blue line represents the wild‐type activity. Top‐performing variants are annotated.

As γ‐dihydroionone (**5**) is a compound of particular interest for the flavour and fragrance industry, we aimed to improve the activity and selectivity of the *Aci*SHC catalyzed conversion of **2** into **5** using structure‐guided directed evolution. Based on the above‐mentioned docking studies of (*E*)‐**2** and (*Z*)‐**2** into a homology model of *Aci*SHC, we chose 14 sites for NNK single‐site saturation libraries. In the first evolution round, we focussed on residues around **2** with the aim to improve pre‐folding. In addition, we targeted the large unoccupied space in the active pocket to limit potentially unproductive binding modes known to occur for small substrates in other SHCs (Figure 2 a).^[11]^ Overall, we screened 90 clones for each of the fourteen libraries in deep‐well plates amounting to the analysis of >1200 enzyme variants. The screening revealed variants with 2.9 to 5.4‐fold increased conversion of **2** into **5** in the libraries A169X, P263X, A310X, G606X, and I613X (Figure 2 b). With the exception of variant G606T, hydrophobic residues were favoured substitutions, and while increased bulk seemed beneficial at sites A169, P263 and A310, smaller amino acids were preferred at I613.

Because all beneficial sites were located in the same area of the active pocket of *Aci*SHC, it seemed plausible that epistatic interactions between the amino acid residues might occur. Going forward, we therefore opted to combine all beneficial mutations and the respective wild‐type amino acid in a five‐site combinatorial library, resulting in a library size of 288 variants. Following library construction by overlap extension PCR, we screened 720 clones for an estimated coverage of 92 %^[29]^ (Figure S6). The best variant for the conversion of (*E*/*Z*)‐**2** identified in the second evolution round was dubbed *Aci*SHC_R2.1 (A169P, A310M, G606C, I613V) and achieved a conversion of **2** into **5** of 21.4 %, a 30‐fold increase over the wild‐type enzyme. In our quest to understand the basis of the increased activity in the engineered *Aci*SHC variants, we sequenced the top ten variants of the second evolution round, all exhibiting a conversion of **2** to **5** of more than 14.4 %. In this analysis, we found nine unique protein sequences with an average of 3.8 mutations. Astonishingly, no single mutation was present in all variants, with the best single site variant, A310F, only occurring in one of the optimized enzymes (Table S3). These findings could indicate that *Aci*SHC can harbor multiple active site geometries, which can induce a productive pre‐folding of **2** for efficient cyclization into **5**.

**Figure 3:**
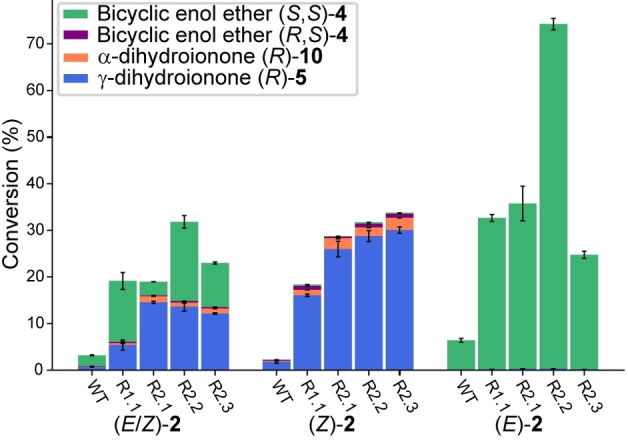
Comparison of the product profile of the wild‐type *Aci*SHC with the best variants from each round of enzyme engineering when supplied with 10 mM (*E*/*Z*)‐geranylacetone (**2**), nerylacetone ((*Z*)‐**2**) or geranylacetone ((*E*)‐**2**). The average total recovery was 86±6 %.

In the analysis of the screening data, we noticed that variants producing high yields of **5** preferentially converted (*Z*)‐**2**. To probe this finding further, we carried out whole‐cell bioconversions with pure (*E*)‐**2** (>99 %) and (*Z*)‐**2** (97 %), prepared by fractionated distillation of the mixture of geometric isomers. Using selected enzyme variants spanning the entire evolutionary trajectory, we found that the biotransformations showed intriguing chemoselectivities in the function of the supplied geranylacetone (**2**) geometric isomer: The best three second‐round *Aci*SHC variants formed almost exclusively monocyclic products **5** and **10** from (*Z*)‐**2** (>96 %) while the bicyclic product **4** was obtained from (*E*)‐**2** (>99 %) (Figure 3).

**Scheme 4:**
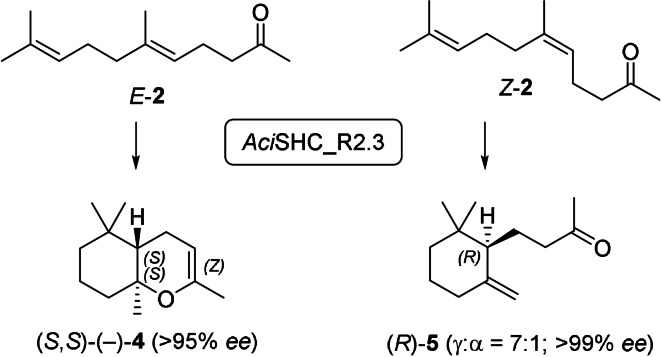
*Aci*SHC_R2.3 catalyzed stereodivergent and enantiospecific cyclization of (*E*)‐ and (*Z*)‐**2** to (*S*,*S*)‐**4** and (*R*)‐**5**, respectively.

Both products were obtained in excellent optical purity: Using variant *Aci*SHC_R2.3, γ‐dihydroionone (**5**) (>99 % *ee*) was formed in the non‐natural laevorotatory form, which could be assigned to the absolute (*R*)‐configuration based on the work of Brenna et al.,^[5]^ whereas the laevorotatory bicyclic enol ether (**4**) (>95 % *ee*) corresponded to the (*S*,*S*)‐configuration as evidenced by comparison to Serra et al.^[30]^ Thus, the SHC enzymes produced the two products **4** and **5** in opposite enantiomeric forms, a process which can be described as a stereodivergent and enantioselective conversion of the (*E*)‐ and (*Z*)‐isomers of **2**. Even when a mixture of (*E*/*Z*)‐**2** was used as substrate, **5** was produced as the (*R*)‐enantiomer and **4** as the (*S*,*S*)‐enantiomer with near to perfect enantioselectivity with all tested variants (Scheme 4, Table S4).

Going forward, we optimized the reaction conditions for *Aci*SHC_R2.3 (A169P, P263W, A310L, I613V), the best second‐round variant for the conversion of the geometric isomer (*Z*)‐**2** to produce (*R*)‐**5** (Figure 3). By optimizing enzyme load (OD 120), temperature (40 °C) and reaction time (48 h), we obtained conversion yields of 79 % for the biocatalytic synthesis of (*R*)‐**5**, underlining the potential of our engineered SHC variant for manufacturing purposes (Figure S7).

**Scheme 5:**
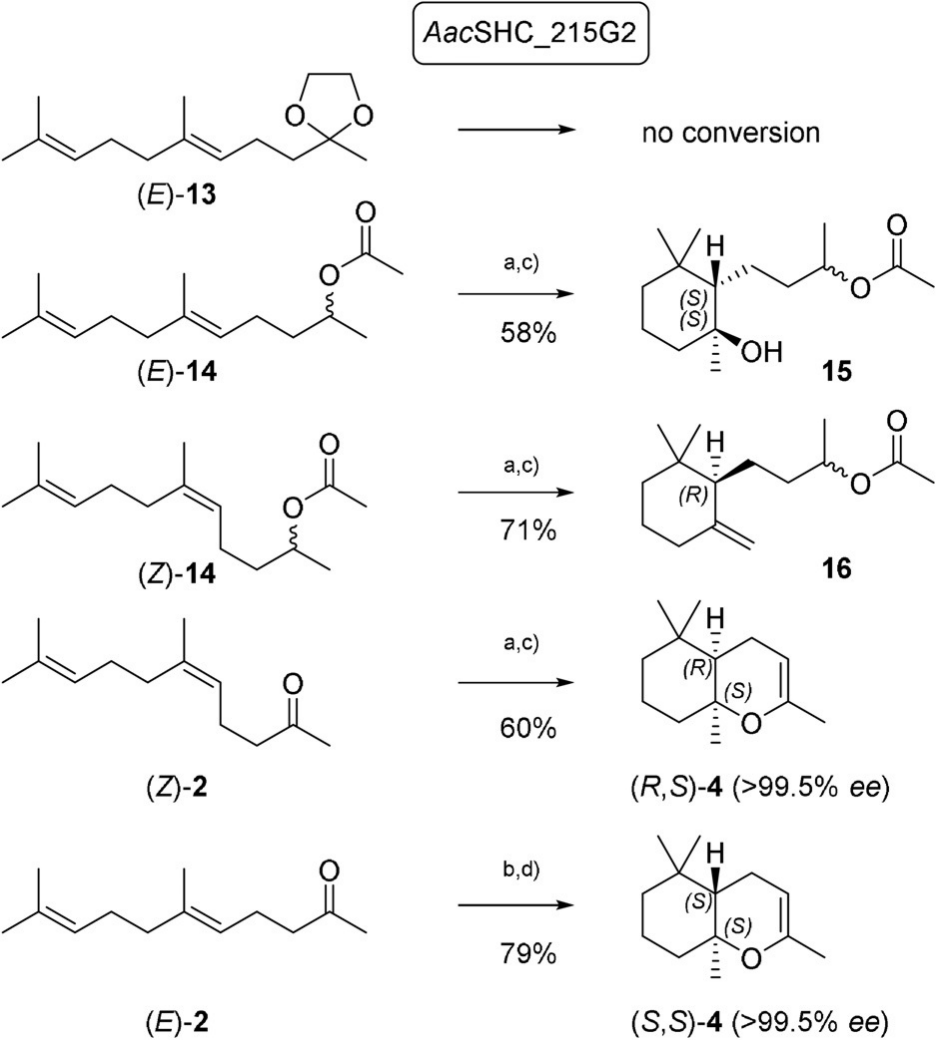
SHC transformations of (*E*)*‐* and (*Z*)‐**14** and **2** with *Aac*SHC_215G2. a) SHC biocatalyst 250 g/l cells, substrate 1.5 g/l (500 mg scale) b) SHC biocatalyst 250 g/l cells, substrate 7 g/l (2 g scale) c) isolated yields after column chromatography d) GC‐yield (isolated yields lower due to partial decomposition of products on SiO_2_).

Going forward, we targeted to evaluate the broader synthetic implications of the observed stereodivergent transformation of geometric isomers by the *Aci*SHC variants. Thus, we set out to transfer our insights to an additional enzyme with the goal to synthesize the (*S*)‐enantiomer of γ‐dihydroionone ((*S*)‐**5**), a key intermediate in the synthesis of (−)‐α‐ambrinol (**6**).^[31]^ Building on our previous results, we hypothesized that for the synthesis of the natural (*S*)‐enantiomer of γ‐dihydroionone ((*S*)‐**5**), we would require a suitable geranylacetone ((*E*)‐**2**) substrate with a masked carbonyl group to prevent the formation of the bicyclic enolether **4**. To that end, the industrially‐proven *Aac*SHC variant *Aac*SHC_215G2 was employed for substrate screening in whole‐cell biotransformations. Whereas no conversion was observed with dioxolane (*E*)‐**13**, we detected the formation of a monocyclic product with intact acetate group from (*E*)‐**14** (Scheme 5). To our surprise, the product was not the expected exo‐methylene derivative **16**, but its hydrated derivative **15**, formed with perfect enantio‐ and diastereocontrol. Intrigued by this observation, we repeated the biotransformation with *Aac*SHC_215G2 and (*Z*)‐**14**, yielding, as expected, the γ‐dihydroionone derivative **16**, again with opposite absolute configuration compared to **15**. These observations prove that the sense of asymmetric induction is determined solely by the geometry of the double bond in the substrate and is not influenced by the presence of the racemic acetate‐bearing chiral center. It is also worth mentioning that no deacetylated product was observed despite the use of a whole cell biocatalyst, where hydrolase‐mediated ester hydrolysis could have been expected.

**Scheme 6:**
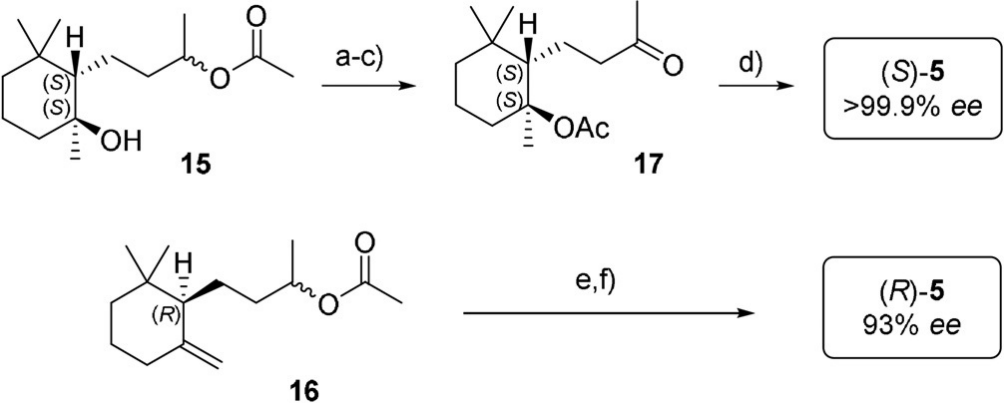
Synthesis of (*S*)‐ and (*R*)‐**5** from cyclotangerinols **15** and **16**. Isolated yields after purification are given. a) acetyl chloride, *N*,*N*‐diethylaniline, chloroform, 82 %; b) MeOH, Mg(MeO)_2_, >95 %; c) PCC, 54 %; d) NaHCO_3_, DMSO, 140 °C, 33 % (γ:α:*β*=80:9:11); e) K_2_CO_3_, MeOH, 97 % f) PCC, 61 %.

To further explore the scope of different SHC/substrate combinations, we performed whole‐cell biotransformations of pure (*E*)‐ and (*Z*)‐**2** with *Aac*SHC_215G2. To our surprise and complementary to the earlier described SHC variants, *Aac*SHC_215G2 converted (*Z*)‐**2** to (*R*,*S*)‐**4** with perfect enantioselectivity, demonstrating that SHCs can fold a (*Z*)‐substrate in such a manner as to form a *cis*‐fused bicycle, in line with the *Stork*‐*Eschenmoser* hypothesis.^[32]^ Finally, (*E*)‐**2** was converted to (*S*,*S*)‐**4** with perfect enantioselectivity and high yield on gram scale by *Aac*SHC_215G2. The chemical transformation of **15** to (*S*)‐**5** (Scheme 6) proved the absolute configuration of **15** and provided the first access to the natural (+)‐enantiomer of γ‐dihydroionone (*S*)‐**5** via asymmetric cation‐olefin cyclization. The same optical purity of (*S*)‐**5** was obtained when tangerinol (**14**; *E*/*Z* 3:2) of commercial quality was used. Similarly, **16** was transformed in two steps to (*R*)‐**5**.^[33]^

**Figure 4:**
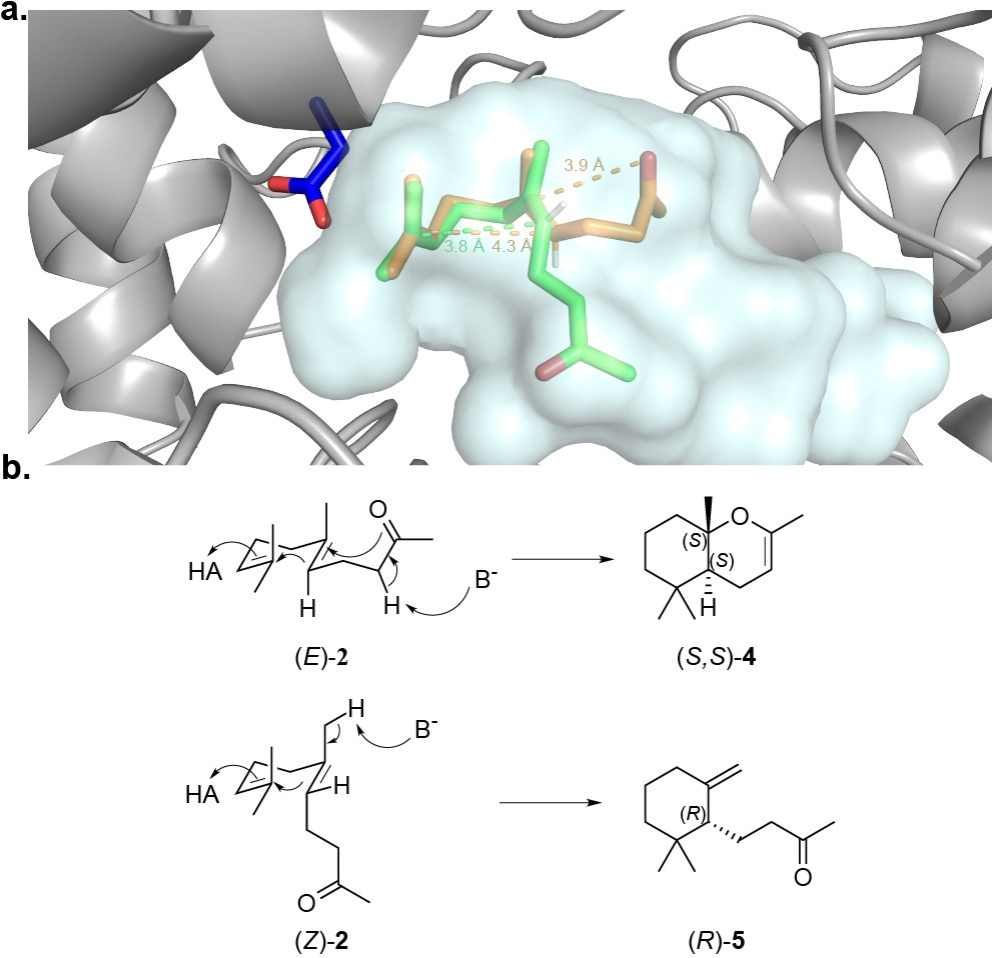
a) Docking of geranylacetone ((*E*)‐**2**) (orange) and nerylacetone ((*Z*)‐**2**) (green) via AutoDock Vina into a homology model of variant AciSHC_R2.3 prepared by SWISS‐MODEL. The corresponding distances for the cyclization reaction are shown. The catalytic aspartate D380 is shown in blue. b) Reaction mechanism for the cyclization of (*E*)‐**2** to the bicyclic product (*S*,*S*)‐**4** and (*Z*)‐**2** to the monocyclic product (*R*)‐**5**.

To better understand the mechanistic basis of these stereodivergent and enantioselective reactions, we generated homology models of the engineered *Aci*SHC_R2.3 and *Aac*SHC_215G2 variants using SWISS‐MODEL^[27]^ followed by molecular docking of (*Z*)‐**2** and (*E*)‐**2** as well as (*Z*)‐**14** and (*E*)‐**14**, respectively, using Autodock Vina.^[28]^ In the homology model of *Aci*SHC_R2.3, (*E*)‐**2** showed a reactive all *pre*‐chair conformer for generation of a bicyclic product, while for (*Z*)‐**2** the second chair was unfolded and the carbonyl‐group too distant for an intramolecular nucleophilic attack (Figure 4 a). Accordingly, the polycyclization cascade is expected to be interrupted by deprotonation, leading to monocyclic products **5** or **10** (Figure 4 b). The pre‐folding of the initial chair for (*Z*)‐**2** and (*E*)‐**2** was nearly identical. Accordingly, the absolute configuration of the newly generated stereocenter resulting from the first cyclization is expected to be defined by the configuration of the double bond (Figure 4). Reflecting our findings for *Aci*SHC, the docking study on *Aac*SHC_215G2 revealed a nearly identical prefolding of the initial *pre*‐chairs for (*Z*)‐**14** and (*E*)‐**14**, suggesting that the enantioselectivity of the cyclization reaction is again determined by the configuration of the double bond of the substrate (Figure S8).

## Conclusion

By screening a comprehensive SHC enzyme library, which expands the current SHC toolbox by ten active enzymes, we identified the novel *Aci*SHC capable to cyclize nerylacetone ((*Z*)‐**2**) into the monocyclic (*R*)‐γ‐dihydroionone ((*R*)‐**5**). To the best of our knowledge, the recent study by the Hauer group^[23]^ and this work are the first examples of SHCs accepting oxygenated isoprenoids with a (*Z*)‐configurated internal double bond, as well as affording a (4a*R*)‐stereocenter after the first cyclization. Interestingly, both studies identified similar hotspots in the enzyme active site influencing the monocyclization reaction, albeit in two different enzyme scaffolds with only 51.6 % identity (Figure S10 and S11). Notably, through our combinatorial enzyme engineering approach, we identified several highly active *Aci*SHC variants comprising divergent active site geometries, which can afford the necessary pre‐folding of **2** to obtain monocyclization products. Our findings therefore indicate that, depending on the enzyme starting scaffold, cyclization cascades cannot only be controlled through the introduction of anchoring hydrogen bonds as shown by Hauer et al.^[23]^ but also through the appropriate choice of the geometric substrate isomer. Transferring this knowledge to the industrially applied *Aac*SHC_215G2 variant, we could highlight that stereodivergent and enantioselective transformations of geometric isomers could indeed prove to be a general principle in SHC catalysis. Through appropriate substrate engineering and downstream processing, we can obtain access to both enantiomers of a target product via SHC biocatalysis, including the industrially highly relevant chiral building block (*S*)‐γ‐dihydroionone ((*S*)‐**5**). Overall, this work provides an exciting opportunity of tuning the absolute configuration of the cyclized products using substrates with defined double bond stereochemistry and highlights the possibility to control the polycyclization cascade through substrate engineering.

## Conflict of interest

The authors declare no conflict of interest.

## Supporting information

As a service to our authors and readers, this journal provides supporting information supplied by the authors. Such materials are peer reviewed and may be re‐organized for online delivery, but are not copy‐edited or typeset. Technical support issues arising from supporting information (other than missing files) should be addressed to the authors.

Supporting Information
