# Supplementary material for: Asymmetric Cation‐Olefin Monocyclization by Engineered Squalene–Hopene Cyclases
Source: Angew Chem Int Ed Engl. 2021 Sep 17;60(50):26080–6. doi: 10.1002/anie.202108037 (PMC9290348; doi:10.1002/anie.202108037)
Supplement: Supplementary file 1 — Supporting Information [file ANIE-60-26080-s001.pdf]

Angewandte Chemie
Eine Zeitschrift der Gesellschaft Deutscher Chemiker

Supporting Information

Asymmetric Cation-Olefin Monocyclization by Engineered Squalene–Hopene Cyclases

*Michael Eichenberger+, Sean Hüppi+, David Patsch+, Natalie Aeberli, Raphael Berweger, Sandro Dossenbach, Eric Eichhorn, Felix Flachsmann, Lucas Hortencio, Francis Voirol, Sabine Vollenweider, Uwe T. Bornscheuer, and Rebecca Buller**

anie_202108037_sm_miscellaneous_information.pdf

Table of Contents

A. Supplementary Methods 1
B. Supplementary Figures 10
C. Supplementary Tables 21
D. NMR Data 25
E. Chiral GC-analysis of SHC-cyclization products 36
F. Sequences 38
G. References 50

A. Supplementary Methods

1. Materials

Sigma, VWR or Carl Roth were the suppliers for all used chemicals. Molecular genetics were carried out with Phusion polymerase, T4-DNA ligase and restriction enzymes from New England Biolabs. Genes and plasmids were ordered from Twist Bioscience and all oligonucleotides from Microsynth AG.

2. Physical measurements

Analytical GC was performed using an Agilent 8890 GC system with an FID and a single quadrupole MSD (5977B MSD) system (Agilent Technologies, California, USA).

3. Selection of SHC variants

We found that all previously characterized squalene-hopene cyclases (SHCs) contain two defining PFAM domains (PF13249, PF13243). In order to access additional natural diversity of SHCs, we extracted all 5633 protein sequences containing these two domains from Uniprot (accessed on 05.03.2018). We aligned the sequences using ClustalOmega [1] and constructed an approximate maximum likelihood tree using FastTree 2.1.10 [2] with default settings. The tree was divided into two major groups, as previously reported [3] – the oxidosqualene cyclases (OSC) of mainly eukaryotic origin and the SHCs of mainly prokaryotic origin.
We then selected a total of 31 enzymes for our SHC library, chosen to span all major parts of the SHC group of the phylogenetic tree (Table S2). Beyond the heavily studied *Aac*SHC and ZmoSHC1, we included a range of biochemically characterized enzymes, six bacterial SHCs with classical SHC activity on squalene [4–8], as well as three fern [9,10] and two *Bacillus* [11,12] terpene cyclases from the SHC family, catalyzing unusual cyclizations of squalene. We also included a total of 18 not previously characterized homologs. Ten putative SHCs originated from thermophilic bacteria, as well as six SHC homologs from the phylogenetic clade containing ZmoSHC1, in order to explore the sequence space around this enzyme with unusually high activity and promiscuity for small C_{10}-C_{20} substrates [5]. Two final variants were from *Bacillus thuringiensis* B 4219, a strain available in our laboratories and *Aspergillus fumigatus* A1163, which derives from a branch of the phylogenetic tree containing fungal SHCs, which had never been experimentally characterized before.

4. Plasmid construction and enzyme expression in 96-well plates

Genes encoding SHCs in pET28b(+) were purchased from Twist Bioscience. Each plasmid was transformed into *E. coli* BL21(DE3). To obtain single colonies, the cells were plated on LB agar plates containing 50 µg/ml kanamycin. Single colonies were picked into 96 well plates to inoculate 1 ml of LB medium containing 50 µg/ml kanamycin for overnight cultures. From these cultures, a glycerol stock containing 100 µl of culture and 100 µl of 50% Glycerol in water was created. For the expression, 500 µl of Zym-5052 auto-induction medium was inoculated with 50 µl from the overnight culture. Expression was carried out at 20 °C for 24 hours with 300 rpm (5 cm shaking diameter) in a Duetz system for plates. Subsequent, the cells were pelleted by centrifugation (4000g) at 4 °C for 15 minutes. The pellet was washed twice with potassium phosphate buffer (100 mM, pH 7). The cell pellet was immediately used for biotransformation reactions or frozen at – 80°C for later usage.

5. Ligand docking and homology modelling

Homology models of SHCs were created with the webserver SWISS-MODEL[13] and default parameters. The crystal structure of *Aac*SHC (PDB ID: 2SQC) or homology models were used for substrate docking simulations. The ligands were prepared in Chemdraw and energy minimized in Chem3D before saving as mol2 files. The docking was carried out with the AutoDock Vina[14] plugin for UCSF Chimera while using the default parameters. The docking results were visually inspected in Pymol.

6. *Aci*SHC single site saturation libraries, biotransformation, and GC-MS analysis

NNK libraries were created by overlap extension PCR using pSHC8 as a template, with primers listed in Table S1. For each library, two fragements were generated in a first round of PCRs. Specifically, the first fragment was created using the universal forward primer T7_fw and the reverse primer designed for the respective library (e.g. L35X_rv). The second fragment was generated using the library forward primer (e.g. L35X_fw) and the universal reverse primer T7_rv. In all cases, the library forward primer contained the

reverse complement of the library reverse primer (overlap), the desired mutation and a silent mutation directly after the desired mutation. All PCRs were carried out with 30 seconds of initial denaturation at 98 °C followed by 29 cycles of 98 °C for 10 seconds, 55°C for 20 seconds and 72 °C for 60 seconds. The final extension was done at 72°C for 10 minutes, and the reactions mixtures were stored at 10 °C until purification. The obtained PCR products were purified by gel extraction, and the DNA concentration was measured via a nanodrop device. Following this step, the two fragments from the first PCRs were used in equimolar amounts as template for a second PCR reaction using primers T7_fw and T7_rv, where they were assembled through the overlap of 15-26bp. The product of the second PCR was purified by gel extraction and afterwards digested with *Xho*I and *Xba*I at 37 °C for 1.5 hours. The digested secondary PCR reactions were ligated into a pET28b(+) vector, which was digested with the same restriction enzymes, using T4 DNA ligase according to the manufacturer's protocols.The DNA sequence of library variants was confirmed by the DNA sequencing service provided by Microsynth AG.
The plasmids were expressed in BL21(DE3) in 96-well plates, as described in 4. The obtained cell pellets were resuspended in half the culture volume of (250 µl) sodium citrate buffer (50 mM, pH 6) with 0.2% Triton-X100 and substrate (10 mM). The screening of native SHCs as well as variants of *Aci*SHC towards (*E*/*Z*)-geranylacetone and (*E*/*Z*)-pseudoionone was carried out in 96-well plates, which were sealed with a heat sealer (Thermo Scientific Alps30) with an alumina seal (Axygen, pierceable sealing film). The seals were chosen to minimize loss of substrates through evaporation. The further characterization of *Aci*SHC variants as well as the screening of native SHCs towards squalene was preformed in glass vials with crimped lids to avoid evaporation. The biotransformations were incubated at 30°C with 300 RPM in the Duetz system for 24 hours. The reactions were stopped with the addition of 800 µl ethyl acetate and a mixing time of 20 minutes at 300 RPM in the Duetz system. Afterwards, they were centrifuged at 4000g, 4 °C for 10 minutes.
Each biotransformation sample was analyzed by GC-MS. The upper layer of ethyl acetate was injected into an Agilent 8890 GC-MS system equipped with a single quadrupole MSD and FID over a J&W DB-5ms GC column (30 m x 0.25 mm x 0.25 µm). Helium was used as the carrier gas. For biotransformations of (*E*/*Z*)-geranylacetone, the GC oven was kept isothermal at 130 °C for 10.5 minutes. The flow was set to be constant at 1.5 ml/min. The MSD was scanning from 30-300 m/z at a speed of 6250. For biotransformations with squalene, the oven was adjusted to 130 °C to 220 °C with 40 °C/min followed by an increase to 310 °C with ten °C/min and the MSD was scanning from 30-500 m/z at a speed of 6250 The MSD was used for the identification of substrate and products. The area detected by the FID was used for the calculation of the conversions (area of product peak divided by the summed area over all product and substrate peaks). The total recovery was calculated using an external calibration curve with authentic geranylacetone reference material.

7. Combination of beneficial mutations

We created a library of *Aci*SHC by recombining the best variants at positions:

A169(A,G,P)
P263(P,W)
A310(A,F,M,L)
G606(G,T,C)
I613(I,V,A,L)

The primers were designed to minimize redundancy (each amino acid is only represented by one codon). Primer sequences can be found in Table S1. The variants were created by overlap extension PCR. In a first round of PCRs, the gene was amplified in five fragments using following primer pairs:

fragment 1: T7_fw, A169_rv,
fragment 2: 1:1:1 mix of A169W_fw:A169G_fw:169P_fw and P263_rv,
fragment 3: 1:1 mix of P263WT_fw:P263W_fw and A310_rv,
fragment 4: 1:1:1:1 mix of A310WT_fw:A310F_fw:A310L_fw:A310M_fw and G606_I613_rv,
fragment 5: 3:1:6:2 mix of G606ACC_I613VTT_fw: G606ACC_I613GCA_fw: G606KGC_I613VTT_fw: G606KGC_I613GCA_fw and T7_rv.

These five fragments were mixed in equimolar amounts for assembly in a second PCR using T7_fw and T7_rv primers and cloned into pET28b(+) as described in 6. The total theoretical library size was 288. Overall, we screened 720 variants for 92% library coverage at 10 mM (*E*/*Z*)-geranylacetone substrate load as described in 6.

8. Preparative scale biotransformation with *Aci*SHC variants

For biotransformation on a preparative scale, cells were cultured overnight at 37 °C at 300 rpm in LB-medium containing 50 µg/ml kanamycin. This pre-culture was then used to inoculate Zymo5052 auto-induction medium in a 1:9 ratio. Protein expression was carried out in baffled shake flasks at 20 °C for 24 hours with 180 rpm. Afterwards, cells were centrifuged (4000g) for 15 minutes and

washed over two steps with potassium phosphate buffer (100 mM, pH 7). The cell pellet was resuspended to OD 20 in sodium citrate buffer (50 mM, pH 6) with 0.2% Triton X100 before 40 mM of the substrate was added. The reaction mixture was then incubated at 20 °C for 24 hours at 180 rpm. To increase product concentration, the reaction was quenched and extracted with a much lower ratio of ethyl acetate: reaction volume (1:8) compared to plate screening.

9. Bioconversion reactions with *Aac*SHC variant 215G2

9.1. 215G2 SHC biocatalyst production

The biocatalyst of the *Aac*SHC variant 215G2 was produced in fermentations as described elsewhere.[15]

9.2. Small scale cyclization of tangerinol isomers

Cyclization reactions (1 ml total volume) with 1 g/l *E*- and *Z*- tangerinol (*E*- and *Z*-14) were run with cells that had produced *Aac*SHC 215G2 at OD650nm of 40 and 0.28% SDS in 0.1 M succinic acid/NaOH buffer pH 5.4. The reactions were incubated at 35°C, and under constant agitation (900 rpm, Heidolph Synthesis 1 Liquid 24). After 24h reaction time, the biotransformations were extracted with 1 ml tert-butylmethylether (MTBE) for GC-MS analysis.

9.3. Preparative scale biotransformations with tangerinol and geranylacetone

Bioconversions were run in 350 ml total volume (750 ml InforsHT reactors) with 250 g/l (wet weight) of cells that had produced *Aac*SHC 215G2 in 0.1 M succinic acid/NaOH buffer at pH 5.4, and 35 °C under constant agitation (700 rpm). They contained 1.30% SDS (w/v), 1.5 g/l nerylacetone (*Z*-**2**), 7 g/l geranylacetone (*E*-**2**), 1.5 g/l racemic *E*-tangerinol (*E*-**14**), or 1.5 g/l racemic *Z*-tangerinol (*Z*-**14**). Cell suspensions were prepared by suspending frozen cell pellets in reaction buffer. The cell concentration was determined by centrifuging an aliquot of cell suspension (17210 *g*, 10 min, 4°C), and the required amount of cell suspension calculated for 250 g/l wet weight of cells in the reaction. SDS was added from a 31% SDS stock solution in deionized water. The required volume of succinic acid buffer for a total reaction volume of 350 ml was deduced. To the reactor was added in the following order: substrate, SDS, buffer, cell suspension. The pH of the reactions was carefully set to 5.4 dropwise with 85% H_3PO_4. pH in the reaction was monitored *in situ* and controlled with a calibrated external electrode. pH adjustment was done with 10% H_3PO_4 if required. Conversion was determined by GC-analysis calculating the ratio of product and peak surface areas (details see below). The bioconversion reactions were finally extracted three times with *tert*-butylmethylether (MTBE): 150, 100, and 100 ml. The fractions were analyzed for their product and substrate content by GC-analysis after dilution if required and finally pooled for further processing.

9.4. Sample preparation and gas chromatography analysis

Bioconversion reactions were sampled over time (200 µl samples), and the samples extracted with 200 µl MTBE. After centrifugation (Eppendorf centrifuge 5415C, 14000 rpm), the solvent phase was analyzed by GC-FID. 1 µl solvent phase was injected (split ratio 10, split flow 40 ml/min) onto a 30 m x 0.32 mm x 0.25 µm DB-5 column (Trace 1310 gas chromatograph, Thermo). The column was developed at 4 ml/min H_2 constant flow: 100 °C, 15 °C/min to 200 °C, 120 °C/min to 240 °C, 3 min at 240 °C; inlet and detector temperature: 250 °C.

10. Synthesis of substrates and synthetic transformation of SHC products to γ-dihydroionone

All reagents and reaction solvents were analytical grade, purchased from commercial suppliers and used without further purification. Reactions were monitored by GC-FID (Zebron ZB-5 GC capillary column, 12 m x , 0.32 mm x 0.25 μm, or Zebron ZB-wax, 15 m x , 0.32 mm x 0.25 μm). Flash column chromatography was performed on Biotage silica gel prepacked columns (particle size 20 μm) with the eluent indicated eluents, flow 50 ml/min. All reported yields, unless otherwise specified, refer to spectroscopically and chromatographically pure isolated compounds; isomeric ratios are indicated if appropriate. Routine NMR spectra were recorded on Bruker Avance III HD, 2D NMR spectra were recorded on Bruker Avance-III 600 MHz with 1.7 mm TCI-microcryoprobe). Proton chemical shifts are reported in ppm (δ) relative to tetramethylsilane (TMS), with the solvent resonance employed as the internal standard ($CDCl_3$ δ 7.27 ppm). Data are reported as follows: chemical shift, multiplicity (s = singlet, d = doublet, t = triplet, q = quartet, p = pentet, sext = sextet, h = heptet, m = multiplet, br = broad), coupling constants (Hz) and integration. ^{13}C chemical shifts are reported in ppm from tetramethylsilane (TMS) with the solvent resonance as the internal standard ($CDCl_3$ δ 77.0 ppm;). Mass spectra were recorded with GC-MS (Agilent Technologies 7890A / 5975C) equipped with an SGE BPX5 capillary column (12 m, 0.22 mm i.d. 0.25 µm film) operated at a constant He flow of 1 ml/min. The temperature program started at 50 °C (for 2 min), then with 20 °C/min to 240 °C followed by 35 °C/min to 270 °C (for 3 min). The MS transfer line and ion source temperatures were 250 °C and 230 °C, respectively. The quadrupole MS was equipped with an EI ion source at 70 eV. For high-resolution mass spectra, samples were diluted in methanol and directly introduced in the MS (Thermo Scientific, Q Exactive Orbitrap) by a syringe pump (Chemyx Inc., Fusion 100 T) at a flowrate of 5-10 µl/min. Data was recorded with 70.000 mass resolution using Xcalibur (4.1.50) and analyzed with Xcalibur (4.2.28.14). Optical rotations were determined with Anton Paar MCP 200 Polarimeter at 589 nm and 25 °C. Data are reported as follows: [α]λ temp, concentration (c; g/100 ml), and solvents. Enantiomeric excesses (% e.e.) were determined by GC employing a chiral stationary phase column specified in the individual experiment. The required racemic standards were synthesized in separate experiments (*vide infra*), and the chiral GC methods were optimized to ensure baseline separation of the two enantiomers.

*10.1. Nerylacetone (Z-**2**)*

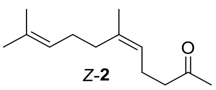

Z-**2**

Commercial geranylacetone (*E*/*Z* 3:2, 767 g) was fractionally distilled over a 1 m Sulzer column (0.08 mbar, bath temperature 125-138 °C). Fractions collected at 82-85 °C contained >92% (*Z*)-isomer (302.5 g). This product (150 g) was further distilled over a 30 cm Sulzer column (0.08 mbar, bath temperature 148 °C), yielding (*Z*)-**2** (100.5 g) as a clear, colourless liquid with an isomeric purity of 97%.

^{1}H-NMR ($CDCl_3$, 400 MHz): 5.07 - 5.14 (m, 2 H), 2.43 - 2.47 (m, 2 H), 2.23 - 2.30 (m, 2 H), 2.14 (s, 3 H), 2.03 - 2.07 (m, 4 H), 1.68 - 1.70 (m, 6 H), 1.62 (s, 3 H). ^{13}C-NMR ($CDCl_3$, 101 MHz): 208.7 (s), 136.5 (s), 131.6 (s), 124.2 (d), 123.3 (d), 44.0 (t), 31.9 (t), 29.9 (q), 26.5 (t), 25.7 (q), 23.3 (q), 22.3 (t), 17.6 (q). MS (EI, 70eV): 194 (M^+, <1), 151 (39), 136 (15), 125 (7), 107 (12), 93 (11), 69 (56), 43 (100).

*10.2. Geranylacetone (E-**2**)*

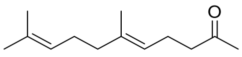

E-**2**

The residue of the above distillation (51 g) contained mainly E-isomer and was distilled over a 10 cm Vigreux column (0.12 mbar, bath temperature 121-122 °C, head temperature 65-73 °C) to yield (*E*)-**2** (35.5 g) as a clear, colourless liquid with an isomeric purity of >99.9%.

^{1}H-NMR ($CDCl_3$, 400 MHz): 5.03 - 5.12 (m, 2 H), 2.43 - 2.49 (m, 2 H), 2.22 - 2.31 (m, 2 H), 2.13 (br. s, 3 H), 1.94 - 2.10 (m, 4 H), 1.67 (br. s, 3 H), 1.62 (br. s, 3 H), 1.60 (br. s, 3 H). ^{13}C-NMR ($CDCl_3$, 101 MHz): 208.9 (s), 136.4 (s), 131.4 (s), 124.2 (d), 122.5 (d), 43.7 (t), 39.6 (t), 29.9 (q), 26.6 (t), 25.7 (q), 22.5 (t), 17.7 (q), 16.0 (q). MS (EI, 70 eV): 194 (M^+, <1), 151 (11), 136 (10), 125 (6), 107 (11), 93 (6), 69 (35), 43 (100).

*10.3. rac. E-Tangerinol (E-**14**)*

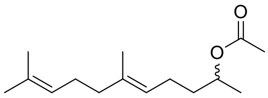

E-**14**

Sodium borohydride (0.77 g, 20.4 mmol, 0.8 equiv.) was added to the solution of geranylacetone (*E*-**2**) (4.96 g, 25.5 mmol, 1 equiv.) in methanol (80 ml) at 0°C. The mixture was stirred for 3 h at room temperature, then poured on 2N aq. HCl-solution (200 ml). The aqueous layer was extracted with MTBE (2x 100 ml), the combined organic layers were washed with water and brine (pH 6), then dried over $MgSO_4$ and concentrated. Crude rac. (*E*)-6,10-dimethylundeca-5,9-dien-2-ol was obtained as a yellow oil (4.90 g, 98%), which was further acetylated (4.0 g, 20.4 mmol, 1 equiv.) in cyclohexane (50 ml) with acetic anhydride (4.2 g, 40.7 mmol, 2 equiv.) and 1 drop of conc. phosphoric acid at room temperature for 18 h. The mixture was poured on 2N aq. NaOH-solution (50 ml), then worked up as described above to yield 4.93 g of a yellow oil which was purified by flash column chromatography with heptane / MTBE 30:1 to yield (*E*)-**14** (*E*-Tangerinol, 4.45 g, 92%) as a clear, colourless oil.
^{1}H-NMR ($CDCl_3$, 100 MHz): 5.05 - 5.14 (m, 2 H), 4.83 - 4.94 (m, 1 H), 2.03 - 2.10 (m, 2 H), 2.03 (br. s, 3 H), 1.95 - 2.02 (m, 3 H), 1.61 - 1.70 (m, 4 H), 1.60 (br. s, 3 H), 1.59 (br. s, 3 H), 1.45 - 1.55 (m, 1 H), 1.25 - 1.31 (m, 1 H), 1.21 (d, *J*=6.1 Hz, 3 H). ^{13}C-NMR ($CDCl_3$, 101 MHz): 170.7 (s), 135.7 (s), 131.3 (s), 124.2 (d), 123.3 (d), 70.7 (d), 39.7 (t), 35.9 (t), 26.7 (t), 25.7 (q), 23.9 (t), 21.4 (q), 20.0 (q), 17.7 (q), 15.9 (q). MS (EI, 70 eV): 178 ([M-AcOH]$^+$, 8), 163 (6), 135 (21), 123 (10), 109 (100), 93 (17), 81 (21), 69 (78), 43 (72).

*10.4. rac. Z-Tangerinol (Z-**14**)*

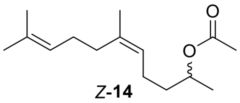

Z-**14**

The procedure described above for the preparation of (*E*)-**14** was repeated with nerylacetone (Z-2) to furnish (*Z*)-Tangerinol (*Z*-**14**, 82% over 2 steps) as a clear, colourless oil.
^{1}H-NMR ($CDCl_3$, 100 MHz): 5.06 - 5.15 (m, 2 H), 4.84 - 4.94 (m, 1 H), 1.96 - 2.09 (m, 6 H), 2.02 (s, 3 H), 1.57 - 1.71 (m, 9 H), 1.46 - 1.54 (m, 1 H), 1.24-1.33 (m, 1 H), 1.21 (d, *J*=6.1 Hz, 3 H). ^{13}C-NMR (CDCl3, 101 MHz): 170.7 (s), 135.8 (s), 131.6 (s), 124.2 (d), 124.2 (d), 70.7 (d), 36.2 (t), 31.9 (t), 26.5 (t), 25.7 (q), 23.8 (t), 23.4 (q), 21.3 (q), 20.0 (q), 17.6 (q). MS (EI, 70 eV): 178 ([M-AcOH]$^+$, 10), 163 (9), 135 (18), 109 (72), 93 (22), 81 (20), 69 (100), 43 (89).

10.5. 4-((1S,2S)-2-hydroxy-2,6,6-trimethylcyclohexyl)butan-2-yl acetate (**15**) *with AacSHC 215G2*

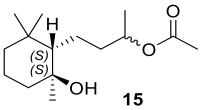

15

The MTBE-extract of the biocatalytic conversion of racemic *E*-Tangerinol (*E*-**14**, 525 mg, 2.2 mmol) with *Aac*SHC 215G2 (250 g/l, 350 ml, cf. chapter 9.3.) was concentrated in a rotary evaporator and the residue was purified via column chromatography (heptane/MTBE 3:2) to yield **15** as a colourless oil (330 mg, 58%, purity by GC/MS > 99.9%, mixture of 2 diastereomers 2:1 according to NMR, not resolved by GC). $[\alpha]_D$ = +2.6 (c = 0.98, $CHCl_3$).
^{1}H-NMR ($CDCl_3$, 100 MHz, mixture of 2 diastereomers): 4.84 - 4.95 (m, 1 H), 2.04 (s, 2 H, main isomer), 2.03 (s, 1 H, minor isomer), 1.21-1.89 (m, 10 H), 1.22 (d, J = 6.2 Hz, 1H, minor isomer), 1.22 (d, J = 6.2 Hz, 2H, major isomer), 1.17 (br. s, 1H, minor isomer), 1.16 (br. s, 2H, major isomer), - 1.07-1.13 (m, 1 H), 0.94 (s, 2 H, major isomer), 0.92 (s, 1 H, minor isomer.), 0.81 (s, 3 H). ^{13}C-NMR ($CDCl_3$, 101 MHz): (main isomer) 171.0 (s), 74.1 (s), 71.6 (d), 57.0 (d), 43.7 (t), 41.5 (t), 39.0 (t), 35.5 (s), 32.8 (q), 23.2 (q), 21.9 (t), 21.4 (q), 21.2 (q), 20.5 (t), 20.0 (q). (minor isomer) 170.9 (s), 74.2 (s), 71.5 (d), 56.7 (d), 43.6 (t), 41.5 (t), 38.7 (t), 35.5 (s), 32.7 (q), 23.3 (q), 21.8 (t), 21.4 (q), 21.2 (q), 20.4 (t), 19.9 (q). MS (EI, 70 eV, mixture of diastereomers): 256 (M$^+$, <1), 196 (4), 181 (7), 163 (8), 153 (13), 138 (15), 127 (15), 109 (56), 96 (35), 81 (20), 69 (38), 55 (25), 43 (100). HR-MS (ESI, positive mode): $C_{15}H_{28}O_3$ calcd. for [M+Na]$^+$ 279.1931, found 279.1931.

10.6. (1S,2S)-(+)-1,3,3-trimethyl-2-(3-oxobutyl)cyclohexyl acetate (**17**)

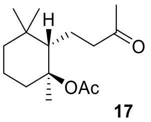

17

The solution of **15** (250 mg, 0.98 mmol), acetyl chloride (1.1 g, 15 equiv.) and N,N-diethylaniline (1.41 g, 9.8 mmol, 10 equiv.) in chloroform (20 ml) was refluxed for 3 days unders stirring. The mixture was added to aq. HCl solution (2 M, 30 ml) and extracted MTBE (2x 30 ml). The organic layers were washed with 30 ml of each, 2 M aq. NaOH solution, water and brine. The combined organic layers were dried over $MgSO_4$, filtered and evaporated to yield 4-((1S,2S)-2-acetoxy-2,6,6-trimethylcyclohexyl)butan-2-yl acetate (240 mg, 82%) as a yellow liquid. Selective hydrolysis of the secondary acetate group was effected by stirring the solution of this product in MeOH (20 ml) in the presence of magnesium methanolate (6.8 ml of a 10% solution in MeOH, 8 equiv.) at room temperature for 30 h. Water (20 ml) was added and the mixture was extracted and worked up as described above to yield a yellow liquid (470 mg >100%). The product was dissolved in CH_2Cl_2 (15 ml) and oxidation of the secondary alcohol was effected by stirring with pyridinium chlorochromate (PCC, 553 mg, 2.6 mmol, 1.4 equiv) for 1.5 h. The mixture was filtered over silica gel and the product was purified by flash column chromatography with heptane / MTBE 3:1 to yield (1S,2S)-1,3,3-trimethyl-2-(3-oxobutyl)cyclohexyl acetate (**17**) as a clear, yellow liquid (110 mg, 44% over 3 steps, purity >99.9% according to GC-MS). $[\alpha]_D$ = +24.6 (c = 1.46, $CHCl_3$) ^{1}H-NMR ($CDCl_3$, 400 MHz): 2.42 - 2.67 (m, 3 H), 2.12 (s, 3 H), 1.92 (s, 3 H), 1.51 - 1.68 (m, 5 H), 1.47 (s, 3 H), 1.31 - 1.43 (m, 2 H), 1.16 - 1.25 (m, 1 H), 0.93 (s, 3 H), 0.83 (s, 3 H). ^{13}C-NMR ($CDCl_3$, 101 MHz): 209.0 (s), 169.9 (s), 87.5 (s), 52.8 (d), 46.0 (t), 40.4 (t), 37.3 (t), 35.5 (s), 32.4 (q), 29.8 (q), 22.9 (q), 22.0 (br., q), 20.4 (t), 20.3 (br., q), 19.6 (t). MS (EI, 70 eV): 254 (M^+, <1), 212 ([M-ketene]$^+$, <1), 194 ([M-AcOH]$^+$, 2), 176 (9), 161 (14), 136 (32), 121 (35), 109 (19), 95 (22), 81 (15), 69 (16), 55 (12), 43 (100). HR-MS (ESI, positive mode): $C_{15}H_{26}O_3$ calcd. for [M+Na]$^+$ 277.1774, found 277.1773.

*10.7. (S)-(+)-γ-dihydroionone ((S)-**5**) from* **17**

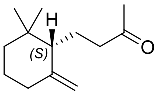

(*S*)-**5**

To the solution of **17** (60 mg, 0.24 mmol) in DMSO (5 ml) was added $NaHCO_3$ (40 mg, 0.47 mmol, 2 equiv.) and the mixture was heated to 140 °C for 30 h. The mixture was poured into water (30 ml) and extracted with heptane. After standard workup and flash column chromatography (heptane / MTBE 10:1), (*S*)-**5** was isolated as a colourless oil (15 mg, 33%). The product contains 9% (*S*)-α-dihydroionone and 11% β-dihydroionone. NMR and MS spectra were in accordance with published data [*C. Fuganti*, *S. Serra*, *A. Zenoni, Helv. Chim. Acta* **2000**, *83*, 2761.]. Chiral GC-analysis (Astec Chiraldex G-DP): 36.34 min, >99.9% e.e.

10.8. 4-((R)-2,2-dimethyl-6-methylenecyclohexyl)butan-2-yl acetate (**16**) *with AacSHC 215G2*

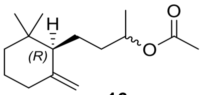

16

The MTBE-extract of a biocatalytic conversion of racemic (*Z*)-**14** (525 mg, 2.2 mmol) with *Aac*SHC 215G2 (250 g/l, 350 ml) as described in chapter 9.3. was concentrated in a rotary evaporator, and the residue was purified via column chromatography (heptane/MTBE 95:5) to yield **16** as a colourless oil (370 mg, 71%). For spectral analysis, a sample was further purified by semipreparative HPLC (normal phase silica, Lichrospher 5 μm, 4 ml/min hexane/ 2.5% MTBE).

^{1}H-NMR (C_6D_6, 600 MHz): 4.99 - 5.06 (m, 1 H), 4.80 - 4.83 (m, 1 H), 4.59 - 4.62 (m, 1 H), 1.91 - 1.95 (m, 2 H), 1.72 (s, 3 H), 1.58 - 1.62 (m, 1 H), 1.37 - 1.51 (m, 5 H), 1.21 - 1.36 (m, 2 H), 1.12 (d, *J*=6.0 Hz, 3 H), 1.06 - 1.11 (m, 1 H), 0.91 (s, 3 H), 0.80 (s, 3 H). ^{13}C-NMR (C_6D_6, 600 MHz, from HSQC): 169. 8 (s), 149.2 (s), 109.6 (t), 71.1 (d), 54.2 (d), 36.0 (t), 35.1 (t), 34.8 (s), 32.3 (t), 28.4 (q), 26.6 (q), 23.9 (t), 22.5 (t), 20.9 (q), 20.0 (q).
MS (EI, 70 eV): 178 ([M-AcOH]$^+$, 5), 136 (55), 122 (29), 109 (58), 93 (61), 81 (31), 69 (49), 55 (25), 43 (100). HR-MS (ESI, positive mode): $C_{15}H_{26}O_2$, calcd. for [M+Na]$^+$ 261.1825, found 261.1825.

10.9. Conversion of **16** *to (R)-***5**

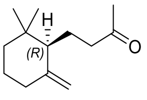

(*R*)-**5**

The above product (300 mg, 1.26 mmol) was saponified with K_2CO_3 (348 mg, 2.52 mmol, 2 equiv.) in MeOH (50 ml) for 3 h under reflux and stirring. The mixture was cooled to RT, poured on water (40 ml) and extracted with MTBE. The organic layers were washed with water and brine and dried over $MgSO_4$. After filtration and removal of the solvent, a colourless oil was obtained (240 mg), which was dissolved in CH_2Cl_2 (15 ml), pyridinium chlorochromate was added (369 mg, 1.71 mmol, 1.4 equiv.), and the mixture was stirred for 2 h at RT. The mixture was filtered over silica gel, the filter cake was rinsed with MTBE, and the filtrate was dried. The residue was purified by FC (heptane/MTBE 93:7) to yield (*R*)-**5** as a colourless oil (145 mg, 61%). The product contained 11% (R)-(+)-α-dihydroionone. NMR and MS spectra were in accordance with published data.[16] Chiral GC-analysis (Astec Chiraldex G-DP): 36.62 min, 93% e.e.

*10.10. Synthesis of rac.-α-dihydroionone (rac.-***10***)*

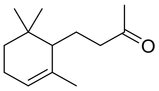

rac.-**10**

Commercial α-ionone (3.0 g, 15.6 mmol) was hydrogenated in THF (40 ml) in the presence of Raney nickel (400 mg) and H_2 (1 atm) for 5 h. The mixture was filtered by suction, and the filtrate was concentrated i. RV to yield rac. α-dihydroionone (2.94 g, 97%). The product was used for subsequent steps without further purification. A sample was purified by flash chromatography on silica gel as racemic reference for chiral GC-analysis (purity according to GC-MS 91%, NMR data were in accordance with published data,[16] MS data matched with MS-library hit. Chiral GC-analysis (Hydrodex-beta-3P): (S)-(-)-10 (43.55 min), (R)-(+)-10 (44.64 min).

*10.11. Synthesis of (R)-(+)-α-dihydroionone ((R)-***10***)*

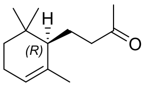

(*R*)-**10**

The procedure described in chapter 10.10. was repeated with a commercial sample of (R)-(+)-ionone (200 mg, 1.04 mmol) to yield (*R*)-**10** as a colourless oil (202 mg, >99%, purity according to GC-MS 98%). $[\alpha]_D$ = +136.9 (c = 1.26, EtOH); (Lit. +138.4 (c = 0.65, EtOH),[17]). Chiral GC-analysis (Hydrodex-beta-3P): 44.61 min, >99.9% e.e.

*10.12. Synthesis of rac.γ-dihydroionone (rac.-***5***)*

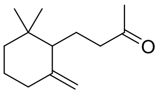

rac.-**5**

To the solution of racemic α-dihydroionone (2.94 g, 15.1 mmol, prepared in chapter 10.10.) in MeOH (40 ml) was added $NaBH_4$ (0.46 g, 12.1 mmol, 0.8 equiv.) at 0°C. The solution was stirred for 15 min; then the cooling bath was removed and stirring continued for 90 min. The solution was poured in 2M aq. HCl-solution (100 ml) and extracted with MTBE. The organic layers were washed with water and diluted NaCl solution, then dried over $MgSO_4$, filtered and concentrated to yield α-dihydroionol (2.78 g, 94%), which was dissolved in CH_2Cl_2 (20 ml). To the solution was added $Ca(OCl)_2$ (2.7 g, 18.8 mmol, 1.3 equiv.) and the mixture was cooled to 5°C, then KH_2PO_4 (6.24 g) in water (30 ml) was added dropwise. The mixture was stirred for 30 h at RT, then poured in water (100 ml), and workup was effected as described above to yield a clear, colourless liquid (2.95 g) which was purified by FC (heptane/MTBE 30:1) to yield 4-(5-chloro-2,2-dimethyl-6-methylenecyclohexyl)butan-2-ol (0.90 g, 28%). This product (0.60 g) was dissolved in THF (20 ml) and AcOH (2.3 g) followed by zinc powder (2.60 g) were added. The mixture was stirred at RT for 21 h, then poured in 2M aq. NaOH-solution (100 ml) and further worked up as described above to yield 4-(2,2-dimethyl-6-methylenecyclohexyl)butan-2-ol (0.6 g)

as a colourless oil. This product was dissolved in CH_2Cl_2 (50 ml) and pyridinium chlorochromate (PCC, 0.92 g, 4.3 mmol, 1.2 equiv.) was added. The mixture was stirred stirred for 3 h at RT. The mixture was filtered over silica gel, the filter cake was rinsed with MTBE, and the filtrate was dried. The residue was purified by FC (heptane/MTBE 95:5) to yield rac.-**5** as a colourless oil (300 mg, 46%, purity according to GC-MS 91%, the remainder being 7% of α-dihydroionone and 2% of β-dihydroionone). NMR and MS spectra were in accordance with published data.[18] Chiral GC-analysis (Astec Chiraldex G-DP): (S)-5 36.34 min, (R)-5 36.62 min.

*10.13. Preparation of (R)-**5** and (S)-**5** by preparative chiral HPLC*

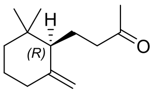

(*R*)-**5**

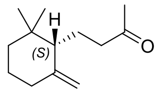

(*S*)-**5**

In order to provide an independent reference of both enantiomers of **5**, racemic **5** as prepared in chapter 10.12. was subjected to preparative chiral HPLC (Daicel, Chiralpak IG amylose-based, 5 µm 10*250mm, flow: 5.6 ml/min, column Temp: 15 °C, isocratic n-Hexane/MTBE 95:5, 20 min, injection of 10 µl of 100 mg/ml sample in n-hexane, (R)-(-)-**5** 11.87 min, (S)-(+)-**5** 13.71 min). From the cumulated fractions of 30 runs, the following samples were isolated after removal of the solvents.

(*R*)-(-)-**5** (16 mg, purity 85% by GC-MS) $[\alpha]_D$ = -17.7 (c = 0.68, $CHCl_3$). Chiral GC-analysis (Astec Chiraldex G-DP): 36.68 min, 98% e.e.
(*S*)-(+)-**5**, (12 mg, purity 98% by GC-MS) $[\alpha]_D$ = +16.4 (c = 0.59, $CHCl_3$). Chiral GC-analysis (Astec Chiraldex G-DP): 36.39 min, 95% e.e.

*10.14. (4aS,8aS)-2,5,5,8a-tetramethyl-4a,5,6,7,8,8a-hexahydro-4H-chromene (S,S)-**4** with AacSHC 215G2*

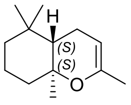

(*S*,*S*)-**4**

The MTBE-extract of the biocatalytic conversion of geranylacetone (*E*-**2**, 2.1 g, 10.8 mmol) with *Aac*SHC 215G2 (250 g/l, 300 ml, cf. chapter 9.3.) contained 79% of product according to GC-FID with external calibration using a racemic product standard. The extract was concentrated in a rotary evaporator, and the residue was purified via column chromatography (pentane/MTBE 60:1; the product is unstable on silica gel and partially decomposes). The combined fractions were concentrated, yielding the product (*S*,*S*)-**4** as a colourless oil (250 mg, 12%, purity by GC/MS 99.4%). $[\alpha]_D$ = -19.0 (c = 1.35, $CHCl_3$). Chiral GC analysis (Hydrodex-beta-3P): 37.70 min, >99.9% e.e.

^{1}H-NMR ($CDCl_3$, 400 MHz): 4.46 (br. dd, *J*=5.3, 0.9 Hz, 1 H), 1.88 - 1.97 (m, 1 H), 1.80 - 1.86 (m, 1 H), 1.72 - 1.80 (m, 1 H), 1.68 - 1.71 (m, 3 H), 1.51 - 1.64 (m, 2 H), 1.39 - 1.51 (m, 3 H), 1.25 - 1.33 (m, 1 H), 1.18 (s, 3 H), 0.92 (s, 3 H), 0.82 (s, 3 H). ^{13}C-NMR ($CDCl_3$, 101 MHz): 148.0 (s), 95.0 (d), 76.4 (s), 48.4 (d), 41.6 (t), 40.0 (t), 33.2 (s), 32.2 (q), 20.8 (q), 20.5 (q), 19.8 (t), 19.2 (t), 19.1 (q). GC-MS (EI, 70 eV): 194 (17), 179 (6), 161 (14), 151 (6), 136 (12), 123 (27), 109 (100), 95 (21), 81 (25), 71 (19), 55 (21), 43 (61). HR-MS (ESI, positive mode): $C_{13}H_{22}O$ calcd. for $[M+H]^+$ 195.1743, found 195.1744.

*10.15. (4aS,8aS)-2,5,5,8a-tetramethyl-4a,5,6,7,8,8a-hexahydro-4H-chromene (S,S)-**4** with AciSHC_R2.1*

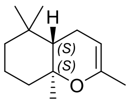

(*S*,*S*)-**4**

The EtOAc-extract of the biocatalytic conversion of geranylacetone (*E*-**2**, 2.4 g, 12.3 mmol) with *Aci*SHC_R2.1 (400 ml reaction, 40 mM substrate, 72 hours at 30 °C) contained 58% of product according to GC-FID. The product was purified as described above in chapter 10.14. to yield (*S*,*S*)-**4** (100 mg, 13%, purity by GC-MS 98%). NMR and MS data were identical to the product isolated in chapter 10.14 Chiral GC analysis (Hydrodex-beta-3P): 37.70 min, >99.9% e.e.

*10.16. Rac.-(4aS,8aS)-2,5,5,8a-tetramethyl-4a,5,6,7,8,8a-hexahydro-4H-chromene (rac. (S,S)-**4**)*

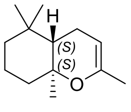

rac.-(*S*,*S*)-**4**

The solution of (*E*)-**2** (2.00 g, 10.3 mmol) in CH_2Cl_2 (40 ml) was cooled to -78 °C, then fluorosulfonic acid (0.65 ml, 11.3 mmol, 1.1 equiv) was added dropwise under stirring. Stirring was continued at -78 °C for 45 min, then at -50 °C for 30 min. Additional fluorosulfonic acid ((0.65 ml, 11.3 mmol, total 2.2 equiv) were added and stirring was continued at -50 °C for 30 min. The solution was poured in 2M aq. NaOH-solution (100 ml) and extracted with MTBE (100 ml). The organic layer was washed with water and brine and dried over $MgSO_4$. The product was purified as described in chapter 10.13 to yield rac.-(S,S)-**4** as a colourless oil (290 mg, 15%, purity according to GC-MS 96%). NMR and MS data were identical to the product in chapter 10.13. Chiral GC-analysis (Hydrodex-beta-3P): (*S*,*S*)-(-)-**4** (37.72 min), (*R*,*R*)-(+)-**4** (38.27 min).

*10.17. (4aR,8aS)-2,5,5,8a-tetramethyl-4a,5,6,7,8,8a-hexahydro-4H-chromene (R,S)-**4** with AacSHC 215G2*

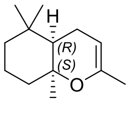

(4a*R*,8a*S*)-**4**

The MTBE-extract of the biocatalytic conversion of nerylacetone (*Z*-**2**, 525 mg, 2.2 mmol) with *Aac*SHC 215G2 (250 g/l, 350 ml, cf. chapter 9.3.) contained 60% of product according to GC-FID with external calibration using a racemic product standard. The extract was concentrated in a rotary evaporator and the residue was purified via column chromatography (pentane/MTBE 60:1; the product is unstable on silica gel and partially decomposes. After careful removal of the solvent the product (4a*R*,8a*S*)-**4** was obtained as a colourless oil (80 mg, 15%, purity by GC/MS 99.5%).
$[\alpha]_D$ = -37.8 (c = 1.02, $CHCl_3$). Chiral GC (Hydrodex-beta-3P): 30.76 min, >99.9% e.e. The absolute configuration was assigned tentatively based on the observed trend of *Aac*SHC 215G2 to yield 4a*S*-configurated products from *Z*-substrates.

1H-NMR (CDCl3, 400 MHz): 4.38 (ddt, J=5.1, 2.1, 1.0, 1.0 Hz, 1 H), 2.15 - 2.24 (m, 1 H), 1.72-1.99 (m, 3 H), 1.67 (dt, J=2.2, 1.3 Hz, 3 H), 1.15 - 1.44 (m, 5 H), 1.17 (s, 3 H), 0.89 (s, 3 H), 0.86 (s, 3 H). 13C-NMR (CDCl3, 101 MHz): 148.7 (s), 94.5 (d), 74.7 (s), 44.0 (d), 42.0 (t), 39.6 (t), 33.7 (s), 32.5 (q), 26.5 (q), 21.2 (q), 20.5 (q), 19.8 (t), 18.1 (t). GC-MS (EI, 70 eV): 194 (7), 179 (6), 151 (6), 136 (6), 124 (14), 109 (100), 95 (8), 81 (11), 71 (14), 55 (12), 43 (27). HR-MS (ESI, positive mode): C13H22O calcd. for [M+H]+ 195.1743, found 195.1743.

10.18. Rac.-(4aR,8aS)-2,5,5,8a-tetramethyl-4a,5,6,7,8,8a-hexahydro-4H-chromene (rac. (4aR,8aS)-4)

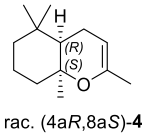

rac. (4a*R*,8a*S*)-**4**

The procedure described in chapter 10.16. was repeated with (*Z*)-**2** (4.0 g) to yield rac.-(4a*R*,8a*S*)-**4** as a colourless oil (750 mg, 19%, purity according to GC-MS 97%). NMR and MS data were identical to the product in chapter 10.17. Chiral GC-analysis (Hydrodex-beta-3P): (4a*R*,8a*S*)-(-)-**4** (29.56 min), (4a*S*,8a*R*)-(+)-**4** (30.77 min) (attribution of absolute configuration cf. chapter 10.17.).

B. Supplementary Figures

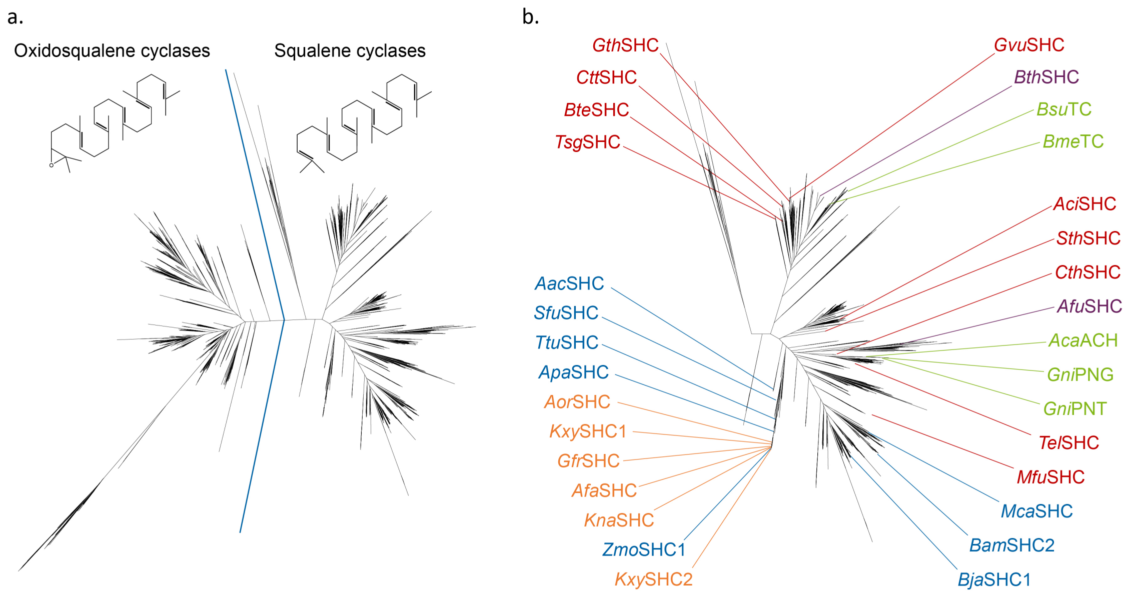

Figure S1: a) An approximate maximum likelihood tree of all 5628 sequences in Uniprot (database accessed on 05.03.2018) containing the two PFAM domains PFAM13249 PFAM13243 found in all SHCs. The tree is split into oxidosqualene cyclases (OSCs) of mainly eukaryotic origin and squalene-hopene cyclases (SHCs) of mainly prokaryotic origin. Also shown are the two standard substrates for enzymes of the respective families **b)** Close-up of the part of the phylogenetic tree containing SHCs and the location of the 31 SHCs investigated in this study. These span most major branches of the phylogenetic tree. The SHCs are colour coded. Blue: Bacterial SHCs converting squalene into hopene and hopanol. Green: Plant and *Bacillus* SHCs catalyzing unusual cyclization of squalene. Red: Novel SHC homologs from thermophilic bacteria. Orange: Novel SHC homologs from the clade, which includes the promiscuous ZmoSHC1 variant. Purple: Novel uncharacterized SHC homologs.

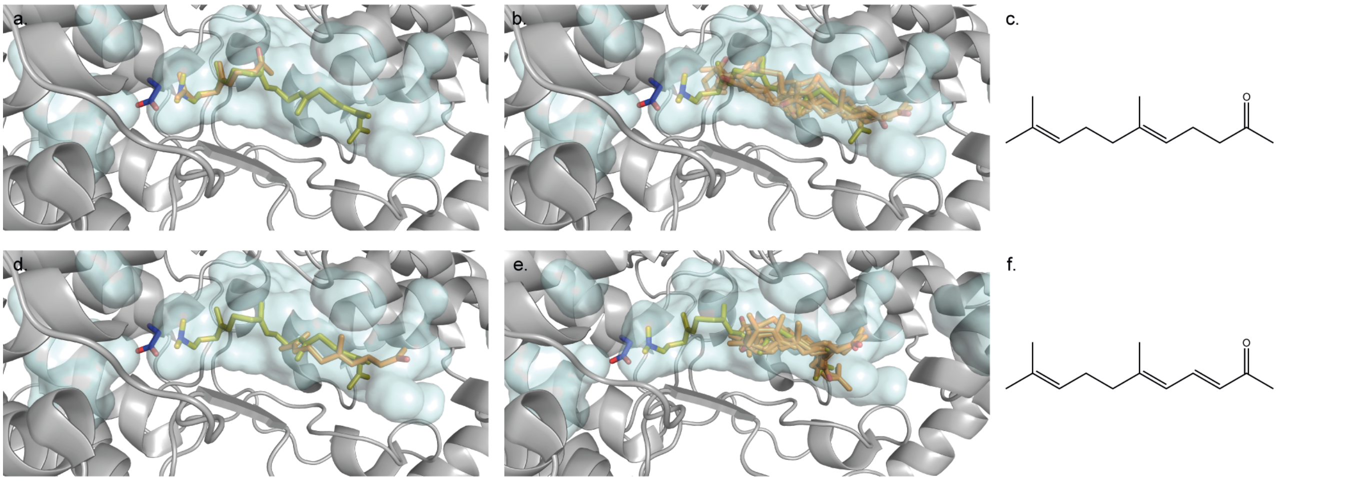

Figure S2: Docking of geranylacetone ((*E*)-**2**) **a)**: best pose, **b)**: 9 best poses and (*E*)-pseudoionone ((*E*)-**1**) **d)** best pose, **e)**: 9 best poses into the active pocket of *Aac*SHC (1UMP). The docked molecules are shown in orange, the cocrystallized 2-azasqualene in yellow and the catalytic acid D376 in dark blue. For geranylacetone ((*E*)-**2**), the best docking mode has a very similar pre folding to 2-azasqualene, and the terminal double bond is at an optimal distance of 2.7 Å from D376 for protonation. For (*E*)-pseudoionone ((*E*)-**1**) all nine docking modes are at a distance of more than 8 Å from D376. Therefore, the higher rigidity of **3** due to the additional conjugated γ,δ-double bond might render a productive pre-folding impossible. **c)** Structure of geranylacetone and **f**) (*E*)-pseudoionone.

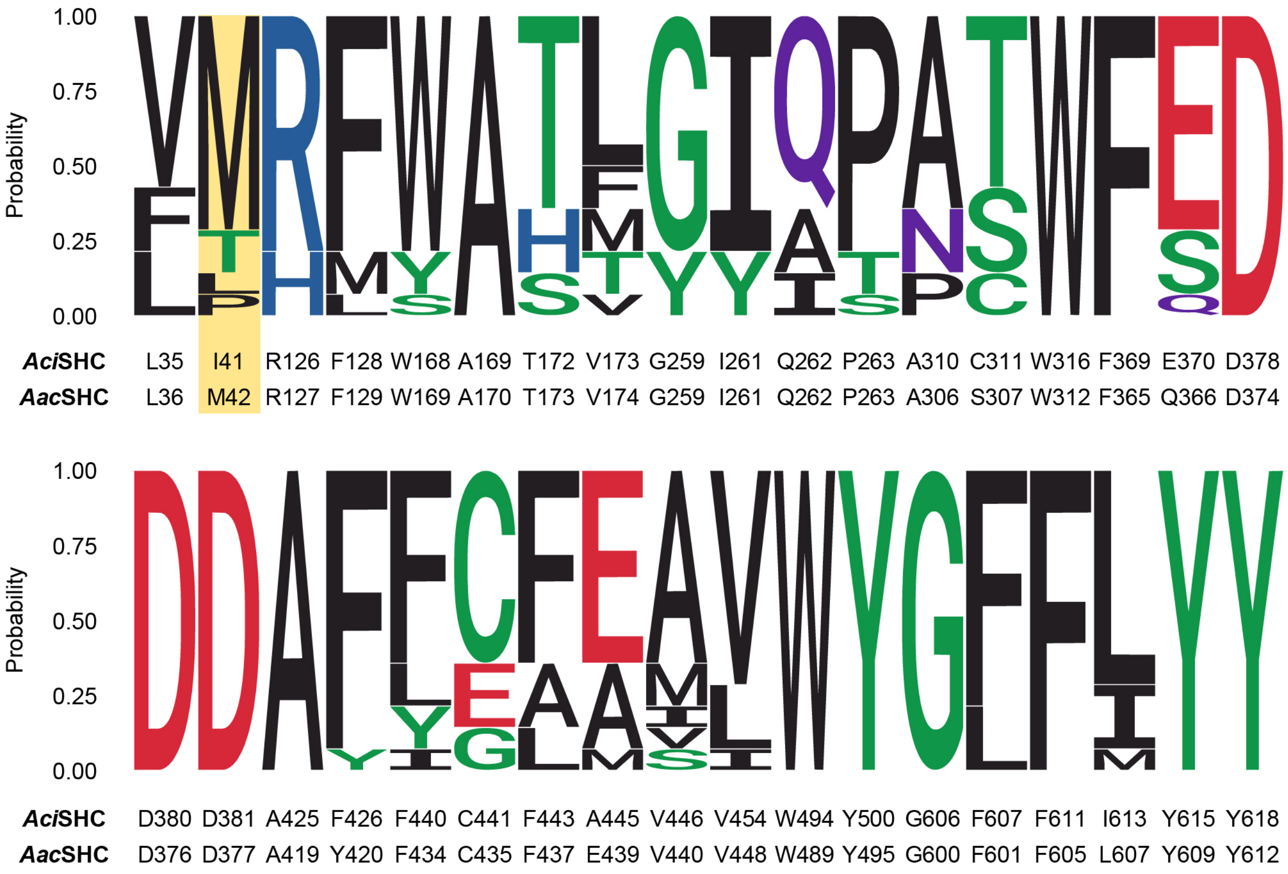

Figure S3: A sequence logo of the active pocket residues [19] of the 14 SHCs converting (*E*/*Z*)-geranylacetone (**2**) into products **4** and **12**. Aligned below are the sequences of *Aci*SHC, which additionally catalyzes the formation of monocyclic products **5** and **10** and *Aac*SHC, as a reference for the usual amino acid numbering of SHCs. Highlighted in yellow is the site for which *Aci*SHC displayse an unique amino acid residue compared to all other SHCs with activity towards **2**.

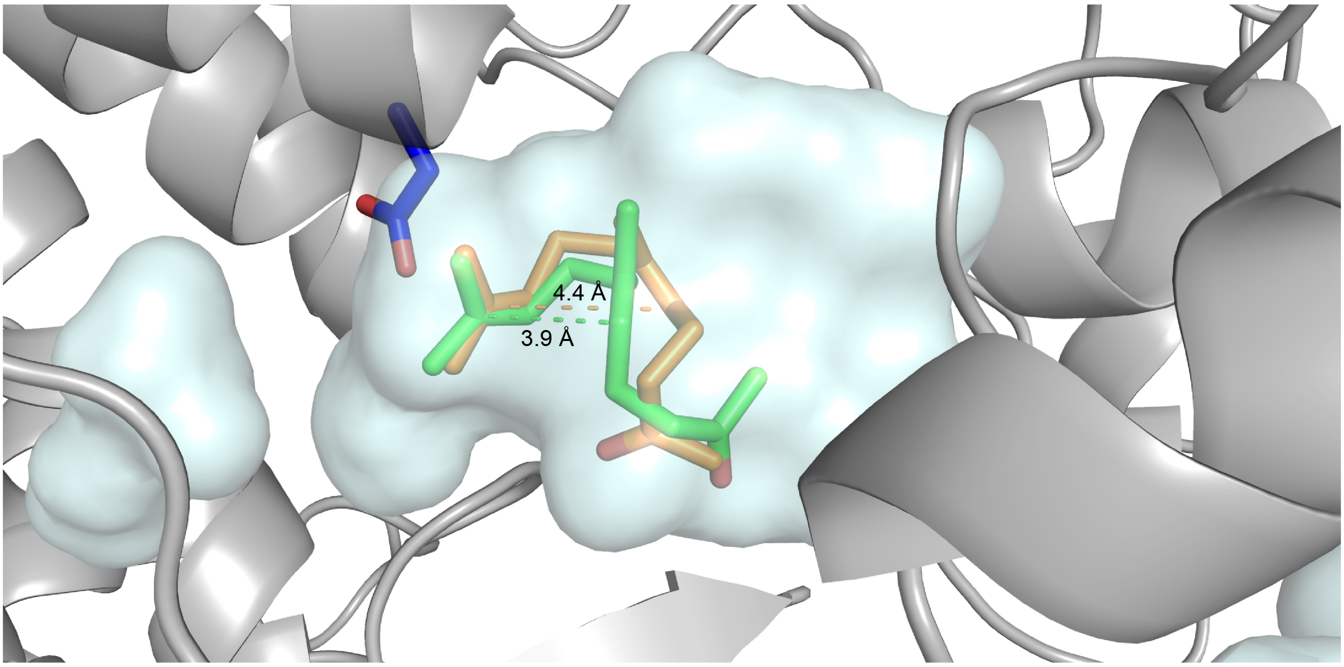

Figure S4: Docking of geranylacetone ((*E*)-**2**) (orange) and nerylacetone ((*Z*)-**2**) (green) via AutoDock Vina into a homology model of *Aci*SHC which was prepared by Swiss model. The corresponding distances for the cyclization reaction are shown. The catalytic aspartate D380 is shown in blue.

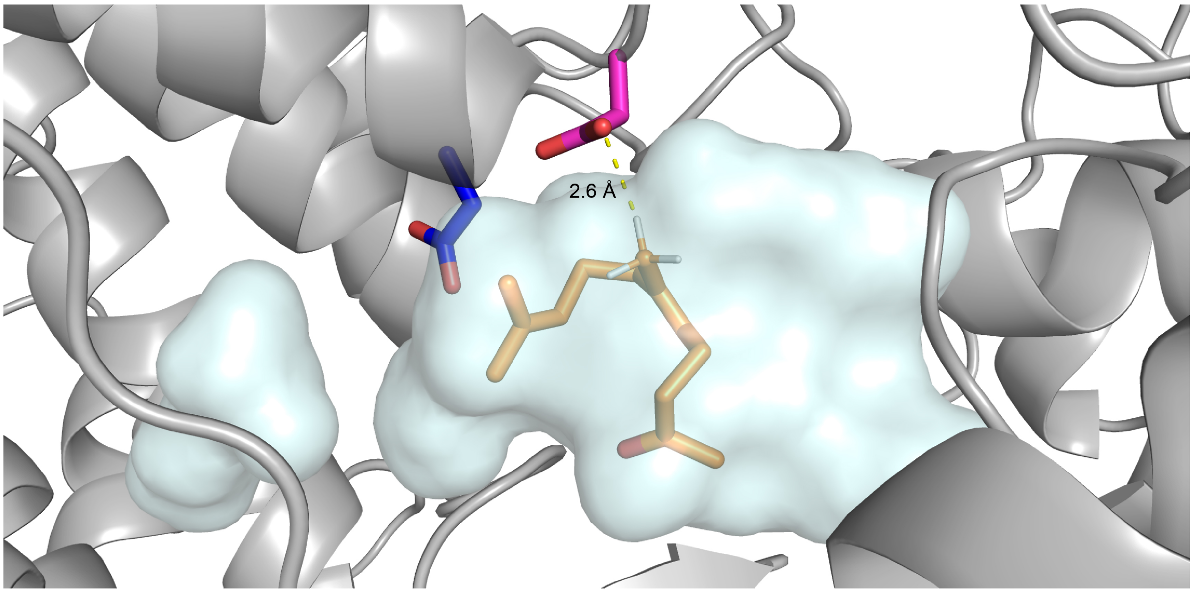

Figure S5: Docking of geranylacetone ((*E*)-**2**) (orange) into a homology model of *Aci*SHC. The distance from the hydrogen of the exocyclic methyl-group of (*E*)-**2** to D378 is shown. The distance of 2.6 Å is within the van der Waals distance between hydrogen and carbon (<2.9 Å) and therefore allows for proton transfer.[20] Therefore, D378 (purple) might be responsible for deprotonation of the monocyclic carbocation intermediate, resulting in the exomethylene containing γ-dihydroionone (**5**). The catalytic aspartate D380 is shown in blue.

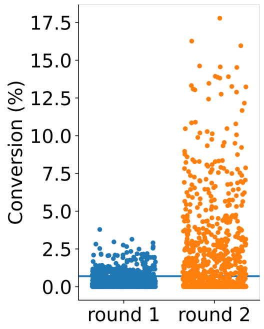

Figure S6: Conversion of (*E*/*Z*)-geranylacetone (**2**) to γ-dihydroionone (**5**) (%FID) by the SHC variants generated in the first and second round of evolution.

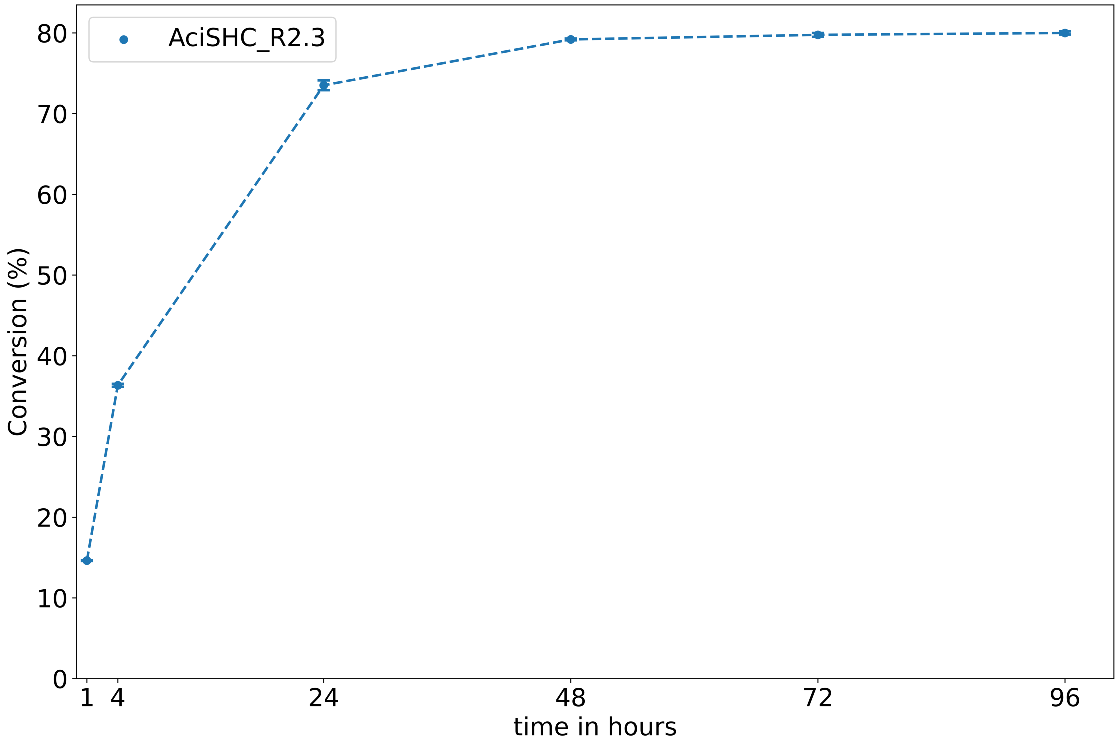

Figure S7: Time course of *Aci*SHC_R2.3. The experiments were conducted at 40 °C with an OD of 120 for 1,4,24,48,72,96 hours in triplicates. We employed 10 mM (*Z*)-**2** as substrate and measured the conversion in % FID to γ-dihydroionone ((*R*)-**5**). The average total recovery was 102±5%.

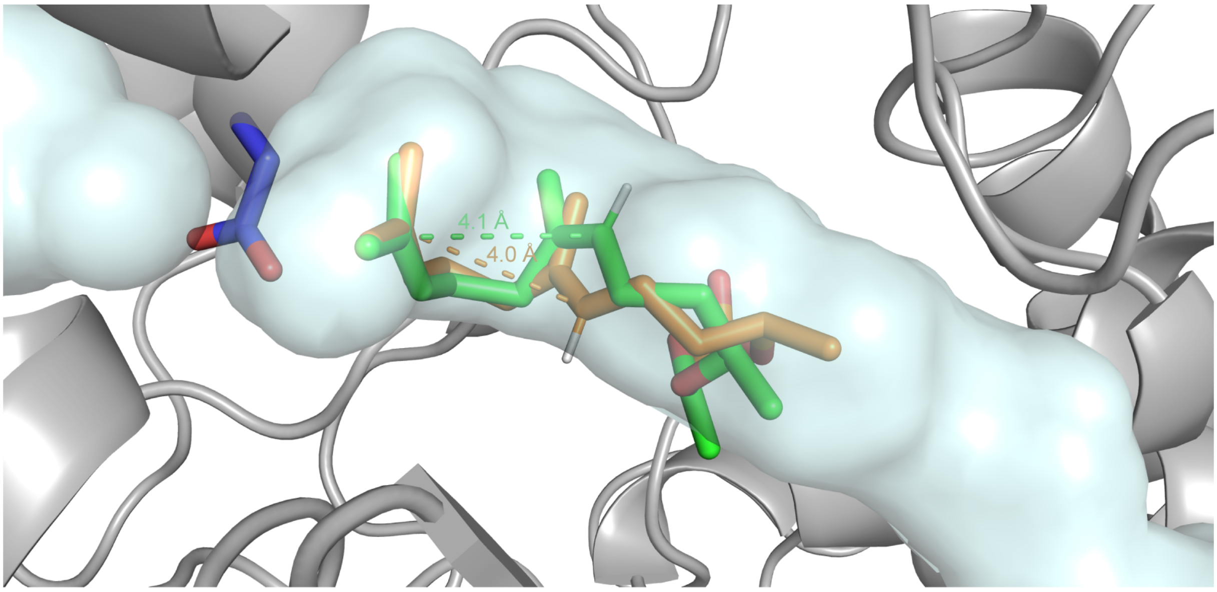

Figure S8: Docking of *(E)*-tangerinol ((*E*)-**14**) (orange) and (*Z*)-tangerinol ((*Z*)-**14**) (green) via AutoDock Vina into a homology model of *Aac*SHC 215G2 prepared by Swiss model. The corresponding distances for the cyclization reaction are shown. The catalytic aspartate D376 is shown in blue.

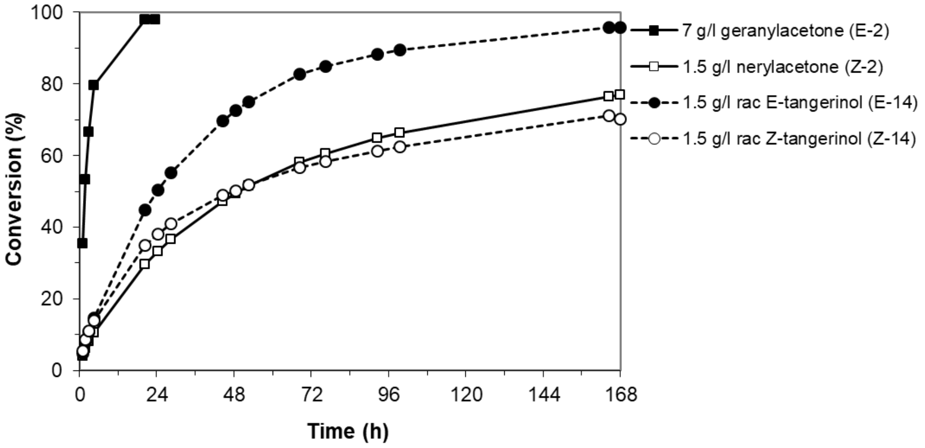

Figure S9: Preparative scale cyclization of geranylacetone (**2**) and tangerinol (**14**) with *Aac*SHC 215G2 (250 g/l cells wet weight). Geranylacetone (**2**): plain lines, racemic tangerinol (**14**): dotted lines. Almost full conversion (95.6%) was obtained after 168 h with racemic *E*-tangerinol (*E*-**14**). Conversion with racemic *Z*-tangerinol (*Z*-**14**) was only 71.0% in the same time. Geranylacetone (*E*-**2**) was fully converted in 24 h. Conversion of nerylacetone (*Z*-**2**) was 76% after 168h.

```
                                                    X     *
AciSHC_wt      1 MTQ-ASVREDAKAALDRAVDYLLSLQDEKGFWKGELETNVTIEAEDLLLREFLGIRTPDI
AciSHC_R2.3    1 MTQ-ASVREDAKAALDRAVDYLLSLQDEKGFWKGELETNVTIEAEDLLLREFLGIRTPDI
AacSHC_wt      1 MAEQLVEAPAYARTLDRAVEYLLSCQKDEGYWWGPLLSNVTMEAEYVLLCHILDRVDRDR
AacSHC_V       1 MAEQLVEAPAYARTLDRAVEYLLSCQKDEGYWWGPLLSNVTMEAEYVLLCHILDRVDRDR

AciSHC_wt     60 TAETARWIRAKQRSDGTWATFYDGPPDLSTSVEAYVALKLAGDDPAAPHMEKAAAYIRGA
AciSHC_R2.3   60 TAETARWIRAKQRSDGTWATFYDGPPDLSTSVEAYVALKLAGDDPAAPHMEKAAAYIRGA
AacSHC_wt     61 MEKIRRYLLHEQREDGTWALYPGGPPDLDTTIEAYVALKYIGMSRDEEPMQKALRFIQSQ
AacSHC_V      61 MEKIRRYLLHEQREDGTWALYPGGPPDLDTTIEAYVALKYIGMSRDEEPMQKALRFIQSQ

                       * *                                       *B  B*
AciSHC_wt    120 GGVERTRVFTRLWLALFGLWPWDDLPTLPPEMIFLPSWFPLNIYDWGCWARQTVVPLTIV
AciSHC_R2.3  120 GGVERTRVFTRLWLALFGLWPWDDLPTLPPEMIFLPSWFPLNIYDWGCWPRQTVVPLTIV
AacSHC_wt    121 GGIESSRVFTRMWLALVGEYPWEKVPMVPPEIMFLGKRMPLNIYEFGSWARATVVALSIV
AacSHC_V     121 GGIESSRVFTRMWLALVGEYPWEKVPMVPPEIMFLGKRMPLNIYEFGSWARATVVALSIV

AciSHC_wt    180 SALRPVRPIPLSID--EIR-TGAPPPPRDPAWTIRGFFQRLDDLLRGYRRVADHGPARLF
AciSHC_R2.3  180 SALRPVRPIPLSID--EIR-TGAPPPPRDPAWTIRGFFQRLDDLLRGYRRVADHGPARLF
AacSHC_wt    181 MSRQPVFPLPERARVPELYETDVPPRRRGAKGGGGWIFDALDRALHGYQKLSVH----PF
AacSHC_V     181 MSRQPVFPLPERARVPELYETDVPPRRRGAKGGGGWIFDALDRALHGYQKLSVH----PF

                                       * X*B
AciSHC_wt    237 RRLAMRRAAEWIIARQEADGSWGGIQPPWVYSLIALHLLGYPLDHPVLRRGLDGLNGFTI
AciSHC_R2.3  237 RRLAMRRAAEWIIARQEADGSWGGIQWPWVYSLIALHLLGYPLDHPVLRRGLDGLNGFTI
AacSHC_wt    237 RRAAEIRALDWLLERQAGDGSWGGIQPPWFYALIALKILDMT-QHPAFIKGWEGLELYGV
AacSHC_V     237 RRAAEIRALDWLLERQAGDGSWGGIQPPWFYALIALKILDMT-QHPAFIKGWEGLELYGV

                              XX    *
AciSHC_wt    297 REETADGAVRRLEACQSPVWDTALAVTALRDAGLPADHPRVQAAARWLVGEEVRVAGDWA
AciSHC_R2.3  297 REETADGAVRRLELCQSPVWDTALAVTALRDAGLPADHPRVQAAARWLVGEEVRVAGDWA
AacSHC_wt    296 EL---DYGGWMFQASISPVWDTGLAVLALRAAGLPADHDRLVKAGEWLLDRQITVPGDWA
AacSHC_V     296 EL---DYGGWMFQVSISPVWDTGLAVLALRAAGLPADHDRLVKAGEWLLDRQITVPGDWA

                             B*       * **
AciSHC_wt    357 VRRPGLPPGGWAFEFANDNYPDTDDTAEVVLALRRVRLEDADQQALEAAVRRATTWVIGM
AciSHC_R2.3  357 VRRPGLPPGGWAFEFANDNYPDTDDTAEVVLALRRVRLEDADQQALEAAVRRATTWVIGM
AacSHC_wt    353 VKRPNLKPGGFAFQFDNVYYPDVDDTAVVVWALNTLRLPDERR--RRDAMTKGFRWIVGM
AacSHC_V     353 VKRPNLKPGGFAFQFDNVYYPDVDDTAVVVWALNTLRLPDERR-RRDAMTKGFRWIVGM

                         BX             ** * **       *
AciSHC_wt    417 QSTDGGWGAFDADNTRELVLRLPFCDFGAVIDPPSADVTAHIVEMLAALGMRDHPAT-VA
AciSHC_R2.3  417 QSTDGGWGAFDADNTRELVLRLPFCDFGAVIDPPSADVTAHIVEMLAALGMRDHPAT-VA
AacSHC_wt    411 QSSNGGWGAYDVDNTSDLPNHIPFCDFGEVTDPPSEDVTAHVLECFGSFGYDDAWKVIRR
AacSHC_V     411 QSSNGGWGAFDVDNTSDLPNHIPFCDFGEVTDPPSEDVTAHVLECFGSFGYDDAWKVIRR

                                   *     B
AciSHC_wt    476 GVRWLLAHQEPDGSWFGRWGANHIYGTGAVVPALIAAGVSPDTPPIRRAIRWLEEHQNPD
AciSHC_R2.3  476 GVRWLLAHQEPDGSWFGRWGANHIYGTGAVVPALIAAGVSPDTPPIRRAIRWLEEHQNPD
AacSHC_wt    471 AVEYLKREQKPDGSWFGRWGVNYLYGTGAVVSALKAVGIDTREPYIQKALDWVEQHQNPD
AacSHC_V     471 AVEYLKREQKPDGSWFGRWGVNYLYGTGAVVSALKAVGIDTREPYIQKALDWVEQHQNPD

AciSHC_wt    536 GGWGEDLRSYTDPALWVGRGVSTASQTAWALLALLAAGEEASPAVDRGVRWLVTTQQPDG
AciSHC_R2.3  536 GGWGEDLRSYTDPALWVGRGVSTASQTAWALLALLAAGEEASPAVDRGVRWLVTTQQPDG
AacSHC_wt    531 GGWGEDCRSYEDPA-YAGKGASTPSQTAWALMALIAGGRAESEAARRGVQYLVETQRPDG
AacSHC_V     531 GGWGEDCRSYEDPA-YAGKGASTPSQTAWALMALIAGGRAESEAARRGVQYLVETQRPDG

                           X*   * X X  *
AciSHC_wt    596 GWDEPHYTGTGFPGDFYINYHLYRLVFPISALGRYVNR----
AciSHC_R2.3  596 GWDEPHYTGTGFPGDFYVNYHLYRLVFPISALGRYVNR----
AacSHC_wt    590 GWDEPYYTGTGFPGDFYLGYTMYRHVFPTLALGRYKQAIERR
AacSHC V     590 GWDEPYYTGTTFPGDFYAGYTMYRHVFPTLALGRYKQAIERR
```

Figure S10: Alignment of *Aci*SHC, *Aci*SHC_R2.3, the best variant for conversion of nerylacetone ((*Z*)-**2**) into γ-dihydroionone ((*R*)-**5**) described in this work, *Aac*SHC and *Aac*SHC_V, the best variant for the same reaction from the recent work by the group of Bernhard Hauer.[21] Active pocket residues[19] are annotated: X: residues that were targeted in both protein engineering efforts, B: residues that were only targeted in this work, *: residues that were not targeted by either work. black background: positions mutated in *Aci*SHC_2.3 and/or *Aac*SHC_V.

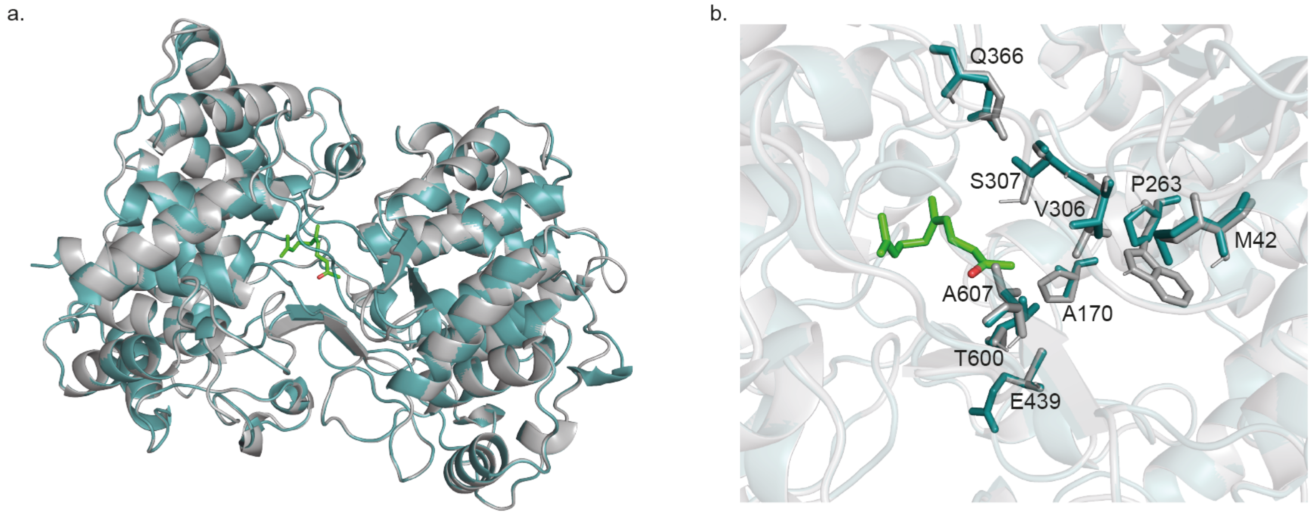

Figure S11: Alignment of homology models of *Aci*SHC_R2.3 (grey), the best variant for conversion of nerylacetone ((*Z*)-**2**) into γ-dihydroionone ((*R*)-**5**) described in this work with *Aac*SHC_V (cyan), the best variant for the same reaction from the recent work by the group of Bernhard Hauer[21]. **a)** Full homology model. **b)** Active site. Position with different amino acid residues between the two engineered enzymes are shown as sticks. Residues are labelled for *Aac*SHC_V. The docking results of (*Z*)-**2** into the homology model of *Aci*SHC_R2.3 using AutoDock Vina is shown in green. As expected, we found that the two homology models are very similar (all atom RMSD: 1.57 Å) because they are both based on the crystal structure of *Aac*SHC (PDB: 1SQC).

C. Supplementary Tables

Table S1: List of all primers used in this study.

Primer Name	Sequence
T7_fw	TAATACGACTCACTATAGGG
T7_rv	GCTAGTTATTGCTCAGCGG
L35X_rv	TTCACCTTTCCAAAAACCTTTTTC
L35X_fw	GAAAAAGGTTTTTGGAAAGGTGAANNKGAGACCAACGTTACCATTGAAG
A169X_rv	CCAACAACCCCAATCATAAATG
A169X_fw	TTTATGATTGGGGTTGTTGGNNKCGCCAGACCGTTGTGC
T172X_rv	CTGACGTGCCCAACAAC
T172X_fw	GTTGTTGGGCACGTCAGNNKGTCGTGCCGCTGACCATTG
I261X_rv	ACCACCCCAGCTACCATC
I261X_fw	GATGGTAGCTGGGGTGGTNNKCAACCTCCGTGGGTTTATAG
P263X_rv	CTGAATACCACCCCAGCTAC
P263X_fw	GTAGCTGGGGTGGTATTCAGNNKCCATGGGTTTATAGCCTGATTG
A310X_rv	TTCCAGGCGACGAACTG
A310X_fw	CAGTTCGTCGCCTGGAANNKTGCCAGAGTCCGGTTTGGG
C311X_rv	TGCTTCCAGGCGACG
C311X_fw	CGTCGCCTGGAAGCANNKCAAAGTCCGGTTTGGGATAC
F369X_rv	TGCCCAACCACCAGG
F369X_fw	CCTGGTGGTTGGGCANNKGAGTTTGCCAATGATAATTACCCG
A425X_rv	ACCCCAACCGCCATC
A425X_fw	GATGGCGGTTGGGGTNNKTTCGATGCAGATAATACCCG
F426X_rv	TGCACCCCAACCGCC
F426X_fw	GGCGGTTGGGGTGCANNKGACGCAGATAATACCCGTGAAC
Y500X_rv	AATATGATTTGCTCCCCAACG
Y500X_fw	CGTTGGGGAGCAAATCATATTNNKGGCACGGGTGCAGTTGTTC
G606X_rv	TGTACCGGTGTAATGCG
G606X_fw	CGCATTACACCGGTACANNKTTCCCGGGTGATTTCTATATTAACTATC
I613X_rv	ATAGAAATCACCCGGAAAACC
I613X_fw	GGTTTTCCGGGTGATTTCTATNNKAATTATCATCTGTATCGCCTGG
Y615X_rv	GTTAATATAGAAATCACCCGGAAAAC
Y615X_fw	GTTTTCCGGGTGATTTCTATATTAACNNKCACCTGTATCGCCTGGTGTTTC
A169_rv	CCAACAACCCCAATCATAAATG
A169WT_fw	CATTTATGATTGGGGTTGTTGGGCGCGCCAGACCGTTGTGCCGCTG
A169G_fw	CATTTATGATTGGGGTTGTTGGGGCCGCCAGACCGTTGTGCCGCTG
A169P_fw	CATTTATGATTGGGGTTGTTGGCCGCGCCAGACCGTTGTGCCGCTG
P263_rv	CTGAATACCACCCCAGCTACC
P263WT_fw	GGTAGCTGGGGTGGTATTCAGCCTCCATGGGTTTATAGCCTGATTG
P263W_fw	GGTAGCTGGGGTGGTATTCAGTGGCCATGGGTTTATAGCCTGATTG
A310_rv	TTCCAGGCGACGAACTGC
A310WT_fw	CAGTTCGTCGCCTGGAAGCATGCCAGAGTCCGGTTTGGGATAC
A310F_fw	CAGTTCGTCGCCTGGAATTTTGCCAGAGTCCGGTTTGGGATAC
A310L_fw	CAGTTCGTCGCCTGGAACTGTGCCAGAGTCCGGTTTGGGATAC
A310M_fw	CAGTTCGTCGCCTGGAAATGTGCCAGAGTCCGGTTTGGGATAC
G606_I613_rv	TGTACCGGTGTAATGCGG
G606ACC_I613VTT_fw	CCGCATTACACCGGTACAACCTTCCCGGGTGATTTCTATVTTAATTATCATCTGTATCGCCTGG
G606ACC_I613GCA_fw	CCGCATTACACCGGTACAACCTTCCCGGGTGATTTCTATGCAAATTATCATCTGTATCGCCTGG
G606KGC_I613VTT_fw	CCGCATTACACCGGTACAKGCTTCCCGGGTGATTTCTATVTTAATTATCATCTGTATCGCCTGG
G606KGC_I613GCA_fw	CCGCATTACACCGGTACAKGCTTCCCGGGTGATTTCTATGCAAATTATCATCTGTATCGCCTGG

Table S2: List of all SHCs used in this study and the plasmids, from which they are expressed. The enzymes are ordered based on their phylogenetic relationship. References on initial characterization of the enzymes are given and previously not characterized SHCs are marked with an asterisk.

SHC	Plasmid	Backbone	Source organism	NCBI Acc. Nr.	Reference
-	pET28b(+)	pET28b(+)	-	-	-
*Kna*SHC	pSHC30	pET28b(+)	*Komagataeibacter nataicola*	AQU88860.1	*
*Kxy*SHC*2*	pSHC32	pET28b(+)	*Komagataeibacter xylinus* E25	AHI26287.1	*
*Afa*SHC	pSHC27	pET28b(+)	*Acetobacter fabarum*	PAK78064.1	*
*Aor*SHC	pSHC28	pET28b(+)	*Acetobacter orleanensis* JCM 7639	GAN69910.1	*
*Kxy*SHC*1*	pSHC31	pET28b(+)	*Gluconacetobacter xylinus*	CUW48332.1	*
*Apa*SHC	pSHC13	pET28b(+)	*Acetobacter pasteurianus*	WP_012812952.1	[6]
*Gfr*SHC	pSHC29	pET28b(+)	*Gluconobacter frateurii* NBRC 103465	GAD08844.1	*
*Zmo*SHC*1*	pSHC5	pET28b(+)	*Zymomonas mobilis*	WP_011241313.1	[5]
*Sfu*SHC	pSHC16	pET28b(+)	*Syntrophobacter fumaroxidans*	WP_011698842.1	[6]
*Ttu*SHC	pSHC17	pET28b(+)	*Teredinibacter turnerae*	WP_015819476.1	[6]
*Aac*SHC	pSHC3	pET28b(+)	*Alicyclobacillus acidocaldarius*	WP_012811690.1	[4]
*Aci*SHC	pSHC8	pET28b(+)	*Acidothermus cellulolyticus*	WP_011720532.1	*
*Sth*SHC	pSHC25	pET28b(+)	*Sphaerobacter thermophilus*	WP_012872483.1	*
*Afu*SHC	pSHC12	pET28b(+)	*Aspergillus fumigatus* A1163	EDP50814.1	*
*Cth*SHC	pSHC22	pET28b(+)	*Chloracidobacterium thermophilum*	WP_014100779.1	*
*Tel*SHC	pSHC11	pET28b(+)	*Thermosynechococcus elongatus*	WP_011058142.1	*
AcaACH	pSHC18	pET28b(+)	*Adiantum capillus-veneris*	BAF93209.1	[9]
GniPNT	pSHC7	pET28b(+)	*Goniophlebium niponicum*	BAI48070.1	[10]
GniPNG	pSHC20	pET28b(+)	*Goniophlebium niponicum*	BAI48071.1	[10]
*Mfu*SHC	pSHC10	pET28b(+)	*Methylacidiphilum fumariolicum*	WP_009061034.1	*
*Bja*SHC*1*	pSHC4	pET28b(+)	*Bradyrhizobium japonicus*	CAA60250.1	[7]
*Bam*SHC*2*	pSHC14	pET28b(+)	*Burkholderia ambifaria*	ABI91648.1	[6]
*Mca*SHC	pSHC15	pET28b(+)	*Methylococcus capsulatus*	WP_010960137.1	[8]
BmeTC	pSHC6	pET28b(+)	*Bacillus megaterium*	WP_013083001.1	[12]
BsuTC	pSHC19	pET28b(+)	*Bacillus subtilis*	WP_004399534.1	[11]
*Bth*SHC	pSHC1	pET28b(+)	*Bacillus thuringiensis* B 4219	AJI35613.1	*
*Tsg*SHC	pSHC26	pET28b(+)	*Thermoactinomyces sp.* Gus2-1	KFZ40906.1	*
*Bte*SHC	pSHC21	pET28b(+)	*Brevibacillus thermoruber*	WP_029099368.1	*
*Ctt*SHC	pSHC23	pET28b(+)	*Cohnella thermotolerans*	WP_027091823.1	*
*Gvu*SHC	pSHC9	pET28b(+)	*Geobacillus vulcani*	WP_031409036	*
*Gth*SHC	pSHC24	pET28b(+)	*Geobacillus thermodenitrificans*	WP_029761705.1	*

Table S3: Sequence of the ten best variants of the second round of directed evolution of *Aci*SHC for the conversion of (*E*/*Z*)-geranylacetone (**2**) towards γ-dihydroionone (**5**). Wild type residues are shown in italics, while novel residues are shown in bold. Biocatalytic reactions in screening conditions in deep-well plates were performed with 10mM (*Z*)-**2** towards **5** in triplicates.

Variant	A169	P263	A310	G606	I613	Conversion (%)
*Aci*SHC_wt	*A*	*P*	*A*	*G*	*I*	0.71 ± 0.02
*Aci*SHC_R1.1	*A*	*P*	**F**	*G*	*I*	4.7 ± 0.1
*Aci*SHC_R2.1	**P**	*P*	**M**	**C**	**V**	21.4 ± 1.0
*Aci*SHC_R2.2	**P**	**W**	**M**	*G*	**L**	19.9 ± 0.3
*Aci*SHC_R2.3	**P**	**W**	**L**	*G*	**V**	18.7 ± 0.3
*Aci*SHC_R2.4	**P**	**W**	**M**	*G*	**I**	18.53 ± 0.14
*Aci*SHC_R2.5	**P**	**W**	*A*	**C**	**V**	18.0 ± 0.3
*Aci*SHC_R2.6	**P**	*P*	**L**	**C**	**V**	17.55 ± 0.02
*Aci*SHC_R2.7	**P**	**W**	*A*	**C**	**V**	17.5 ± 0.3
*Aci*SHC_R2.8	**G**	*P*	**L**	**C**	**V**	17.13 ± 0.08
*Aci*SHC_R2.9	**P**	**W**	*A*	*G*	**V**	16.9 ± 1.9
*Aci*SHC_R2.10	**G**	*P*	**F**	**C**	**V**	14.4 ± 1.7

Table S4: Conversion and optical purity of biocatalytic products from (*E*/*Z*)-geranylacetone (**2**) with different variants of *Aci*SHC. Biocatalytic reactions in glass vials were performed with 10mM (*E*/*Z*)-**2** in triplicates.

Variant\Product	(*R*)-γ-dihydroionone ((R)-**5**)		(*S*,*S*)-bicyclic enol ether ((S,S)-**4**)		(*R*)-α-dihydroionone ((R)-**10**)	
	Conversion (%)	ee (%)	Conversion (%)	ee (%)	Conversion (%)	ee (%)
*Aci*SHC_wt	0.70 ± 0.04	>90%	2.34 ± 0.08	>90%	0.08 ± 0.01	n.d.
*Aci*SHC_R1.1	5.4 ± 1.1	>99%	13.0 ± 1.8	>95%	0.4 ± 0.1	>90%
*Aci*SHC_R2.1	14.5 ± 0.3	>99%	2.93 ± 0.03	>95%	1.32 ± 0.01	>90%
*Aci*SHC_R2.2	13.6 ± 1.0	99.7%	17.0 ± 1.4	>95%	0.87 ± 0.08	>90%
*Aci*SHC_R2.3	12.15 ± 0.15	> 99%	9.43 ± 0.23	>95%	1.06 ± 0.02	>90%

SUPPORTING INFORMATION

D. NMR Data

1. Nerylacetone (*Z*-2)

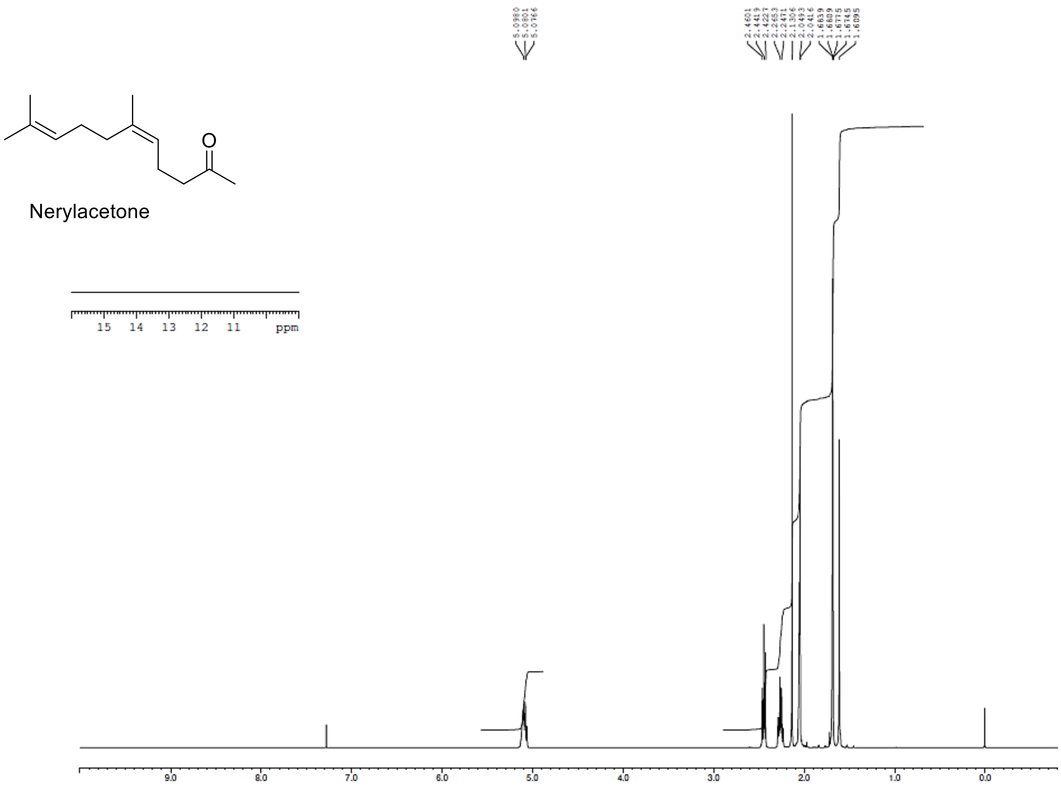

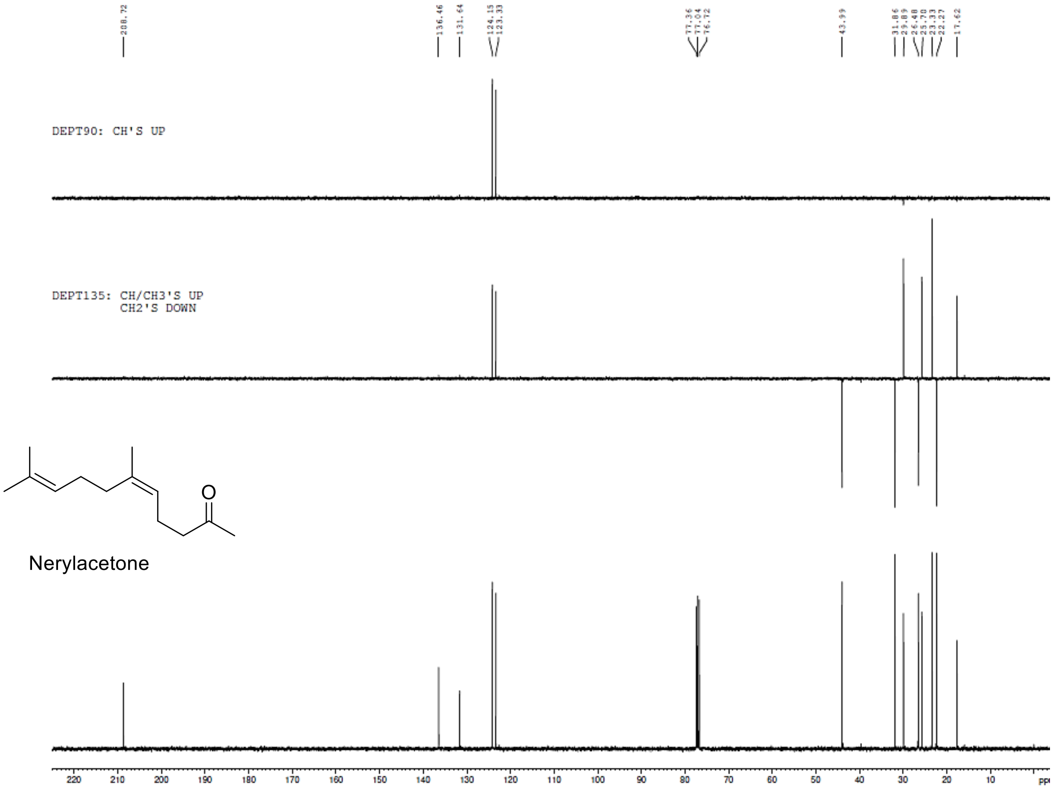

SUPPORTING INFORMATION

2. Geranylacetone (*E*-2)

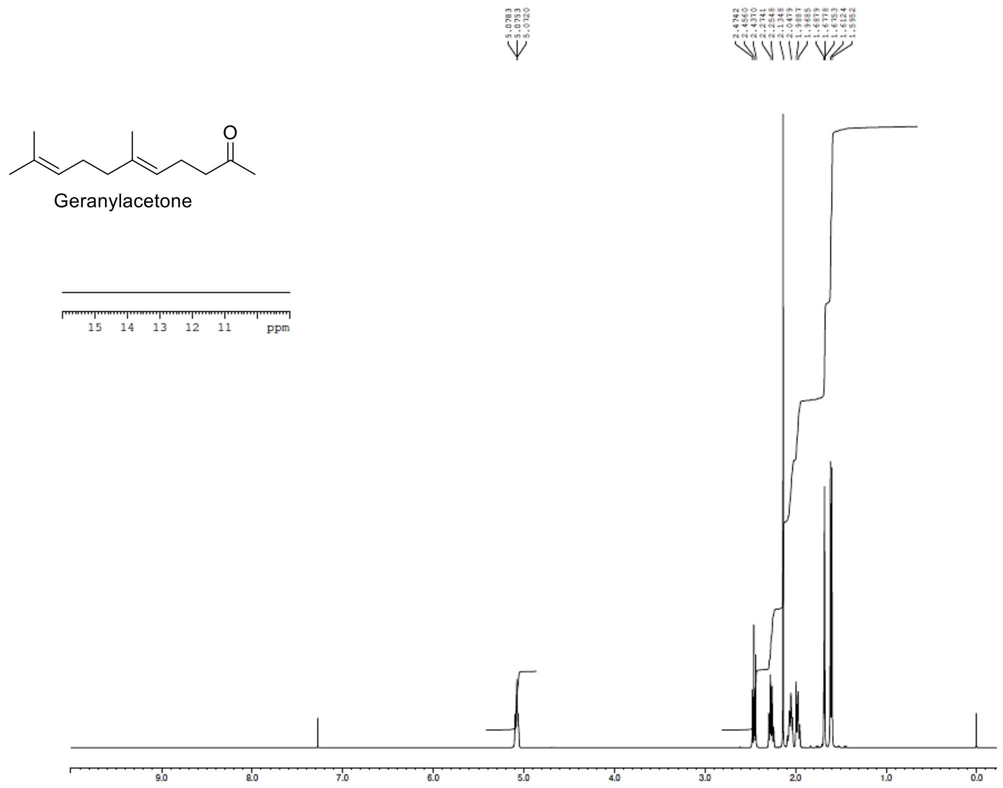

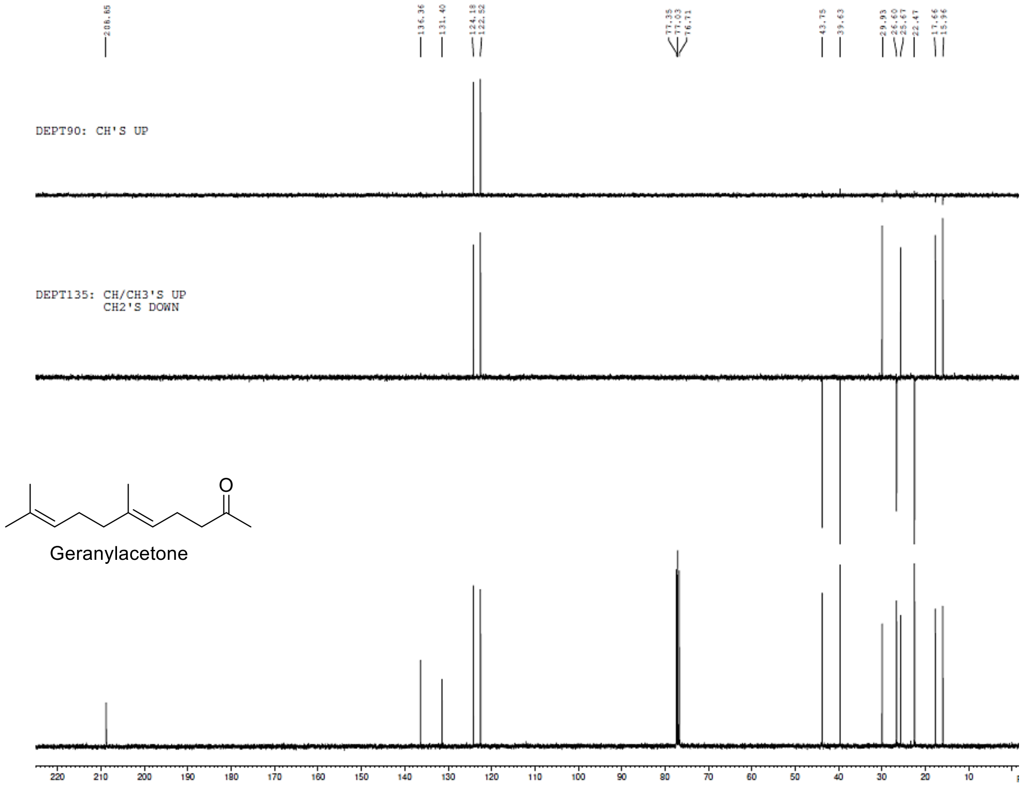

3. *E*-Tangerinol (*E*-14)

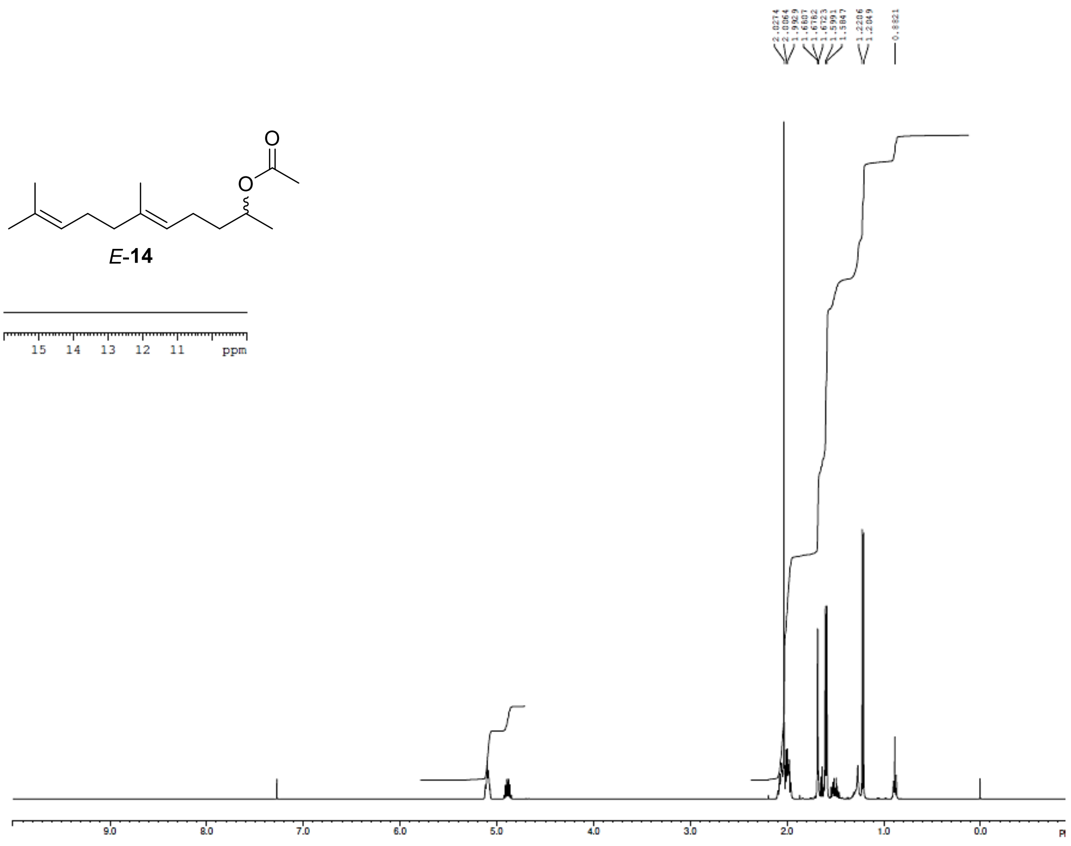

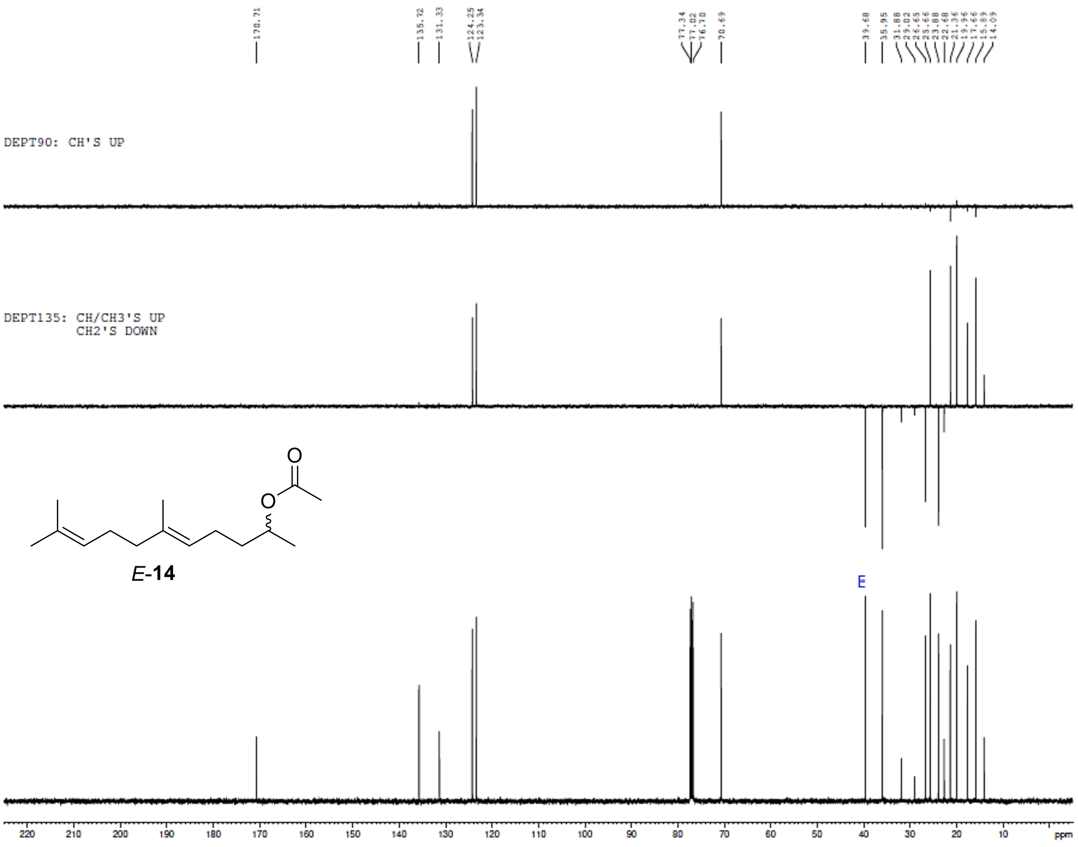

4. ***Z*-Tangerinol (*Z*-14)**

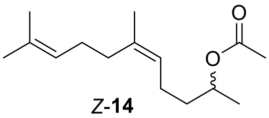

Z-14

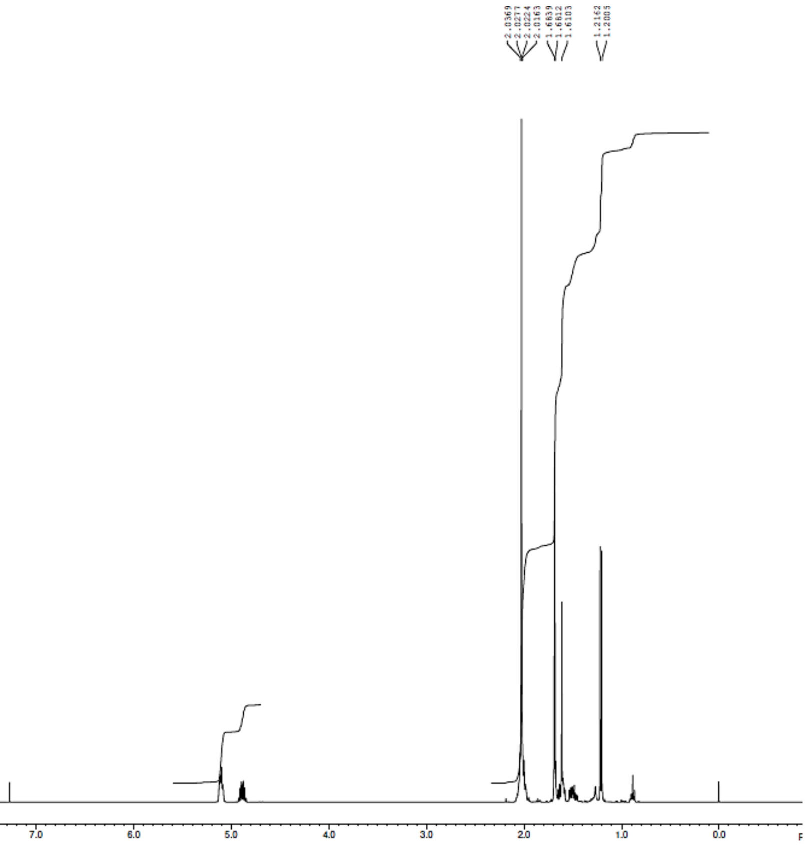

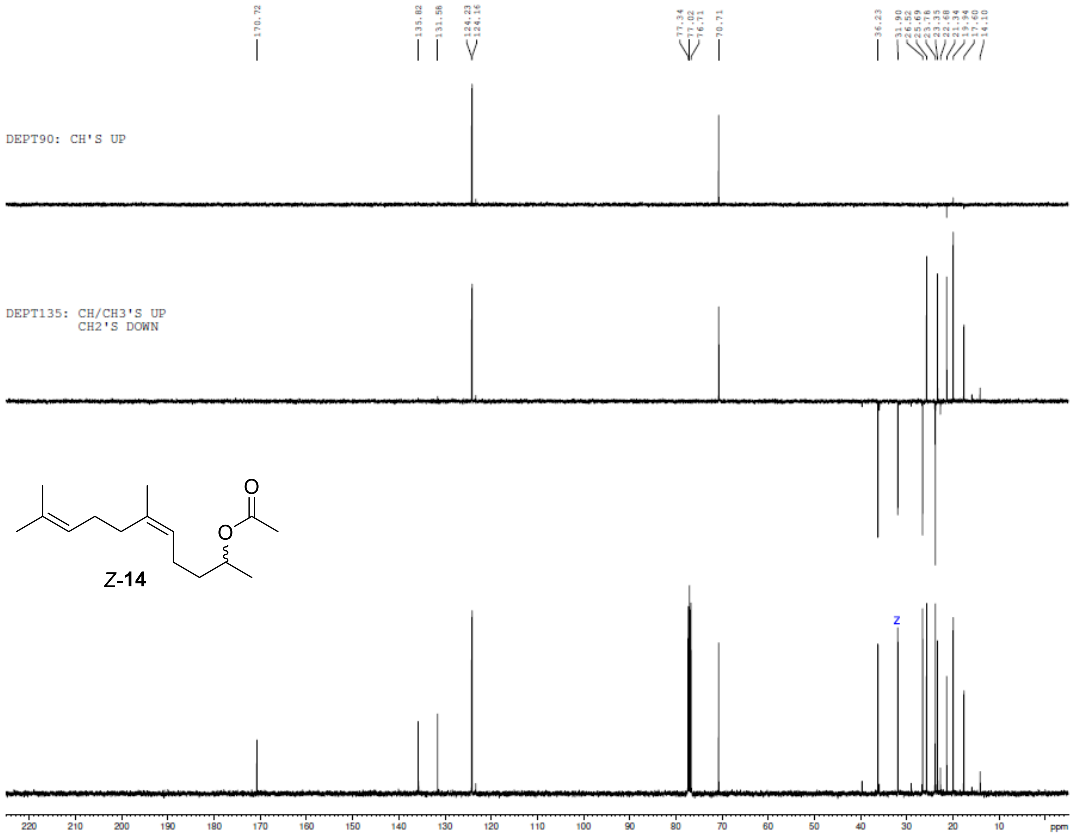

5. 4-((1S,2S)-2-hydroxy-2,6,6-trimethylcyclohexyl)butan-2-yl acetate (15)

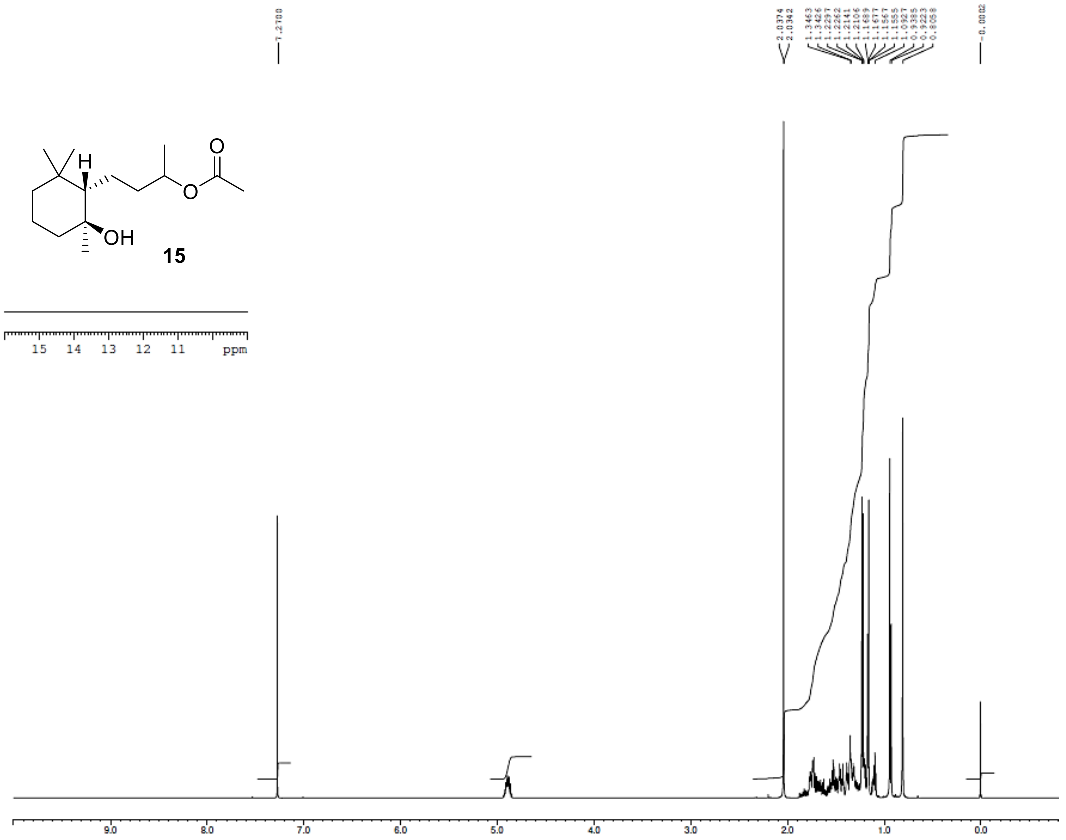

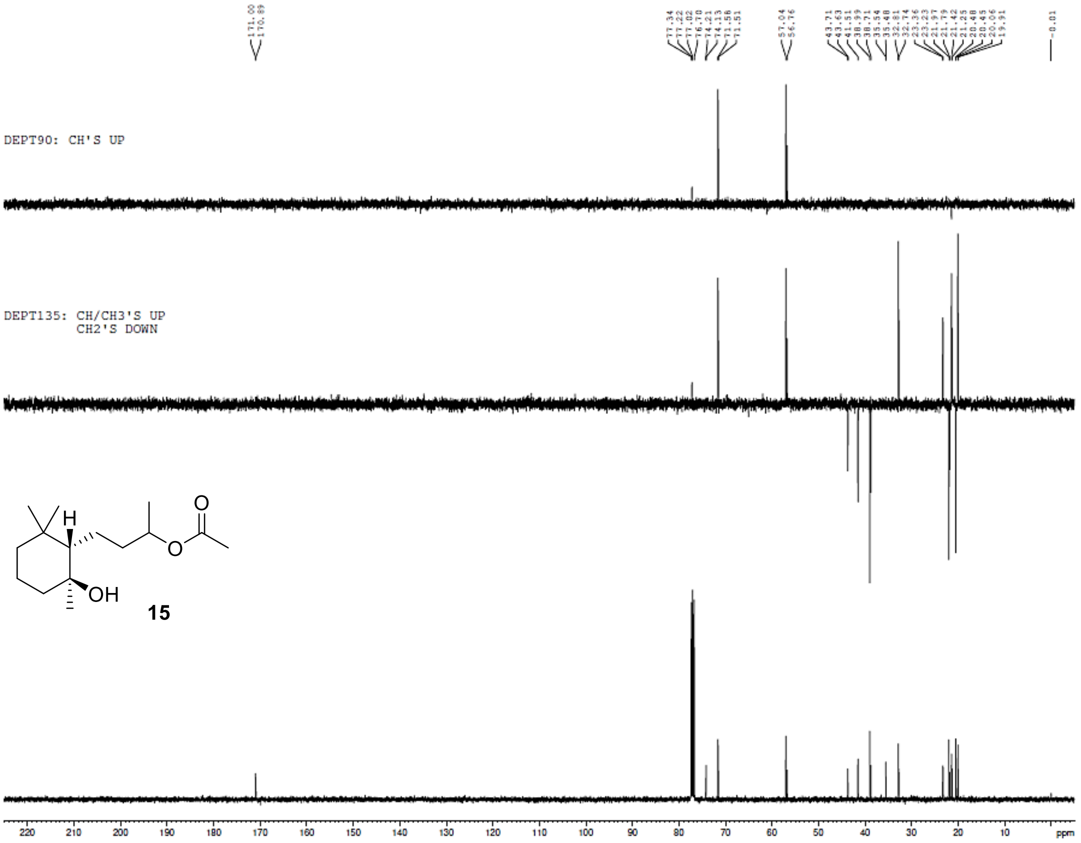

5.1. *HSQC of 4-((1S,2S)-2-hydroxy-2,6,6-trimethylcyclohexyl)butan-2-yl acetate* (**15**)

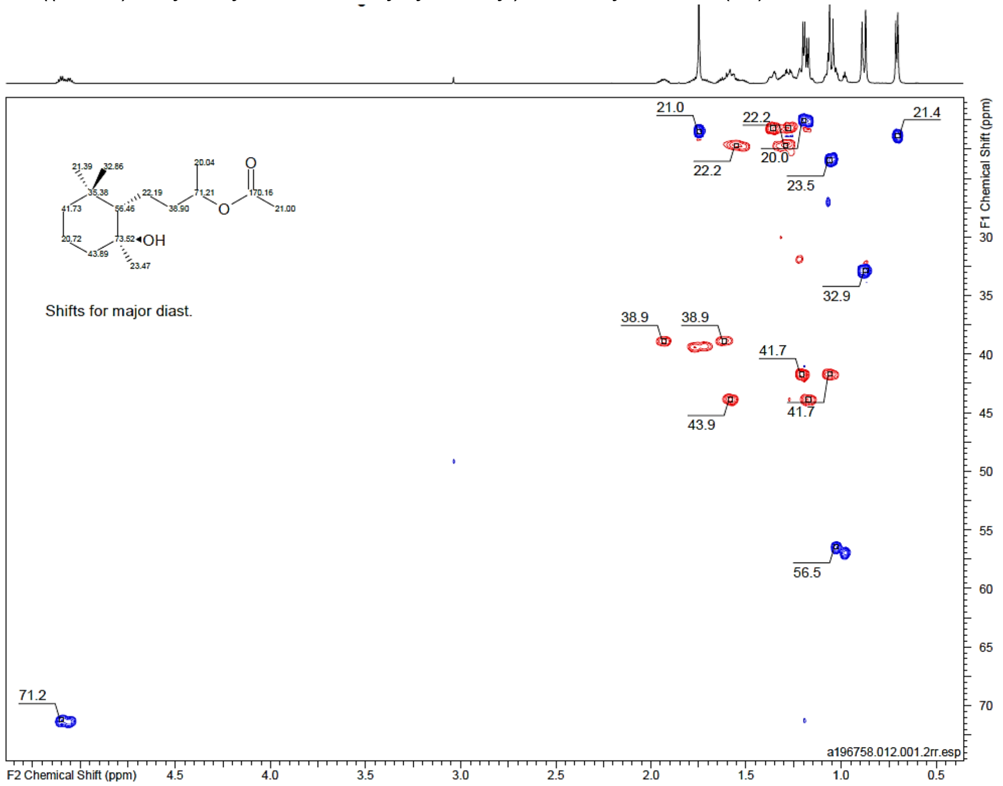

5.2. *NOESY of 4-((1S,2S)-2-hydroxy-2,6,6-trimethylcyclohexyl)butan-2-yl acetate* (**15**)

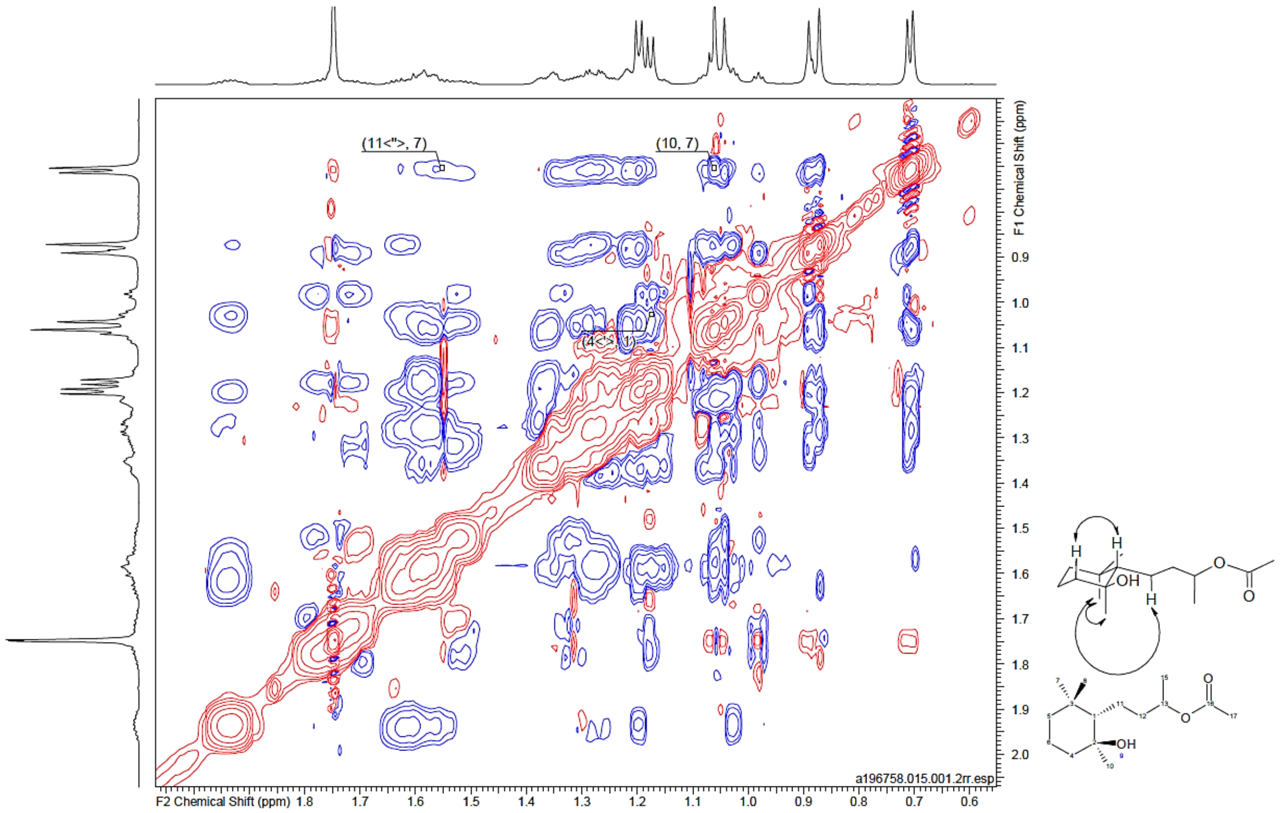

6. (1S,2S)-(+)-1,3,3-trimethyl-2-(3-oxobutyl)cyclohexyl acetate (17)

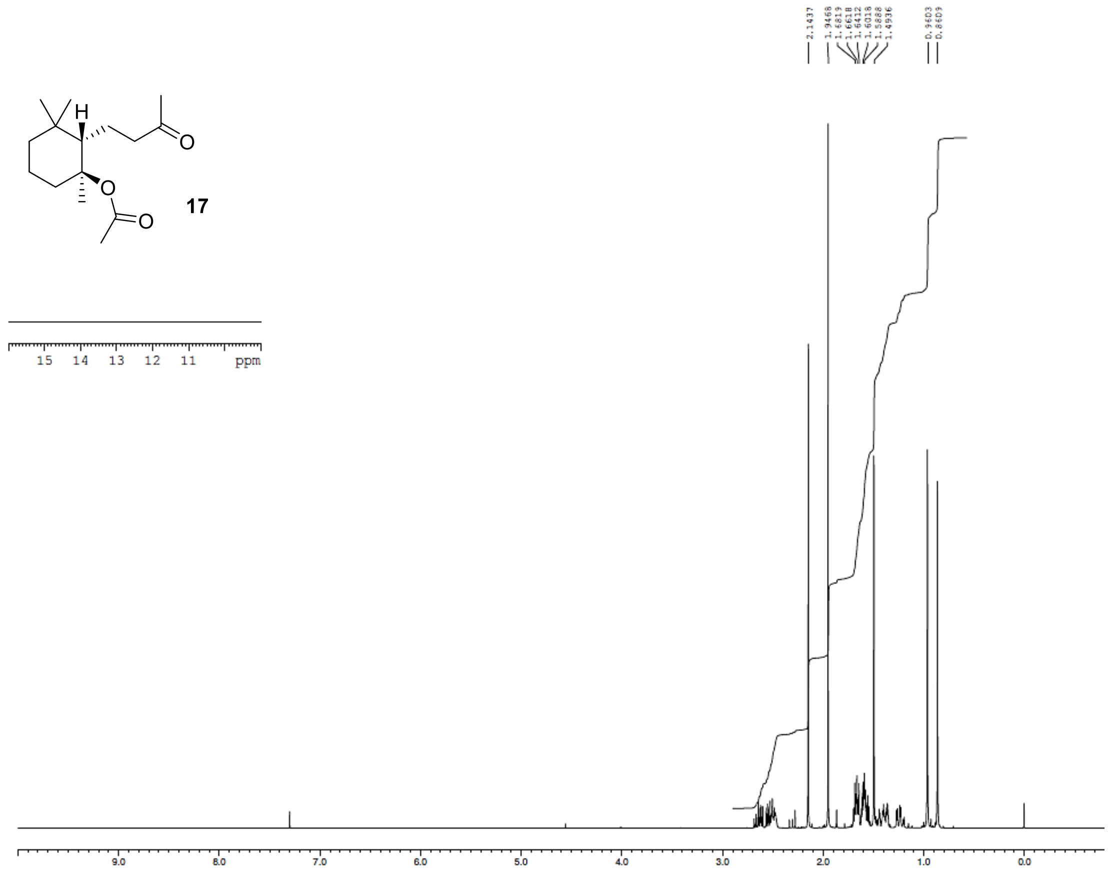

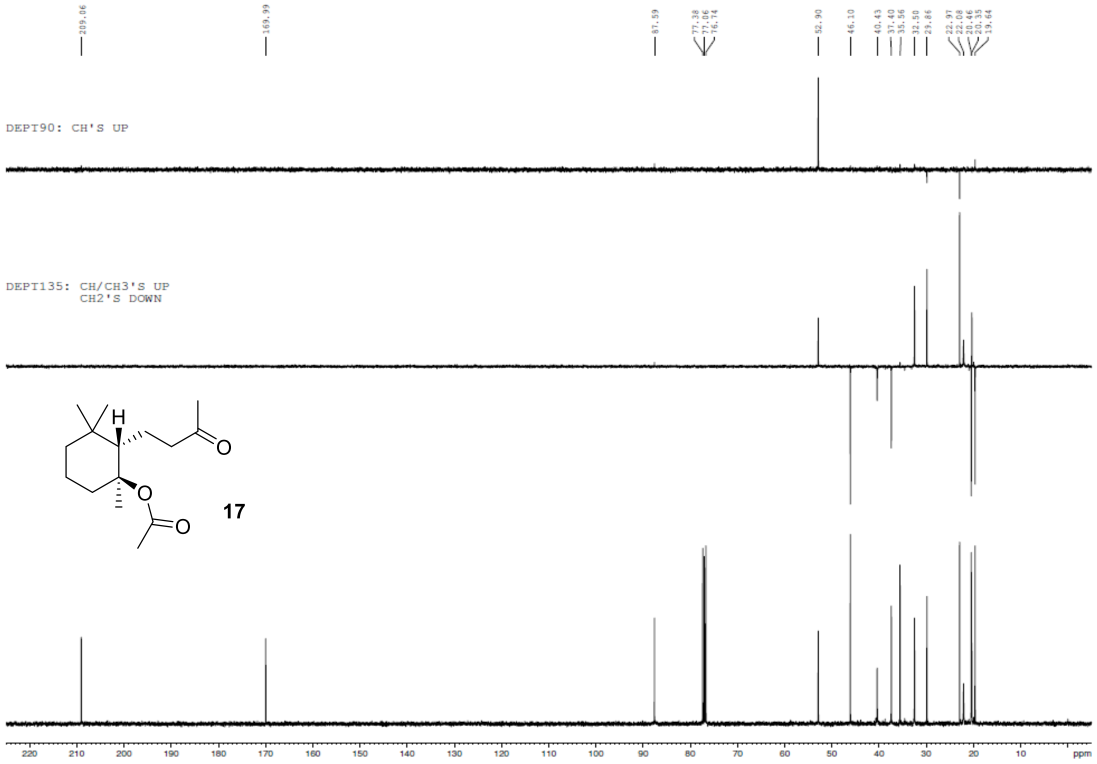

7. (*S*)-(+)-γ-dihydroionone ((S)-5) ex *E*-Tangerinol (γ:α:β 80:9:11)

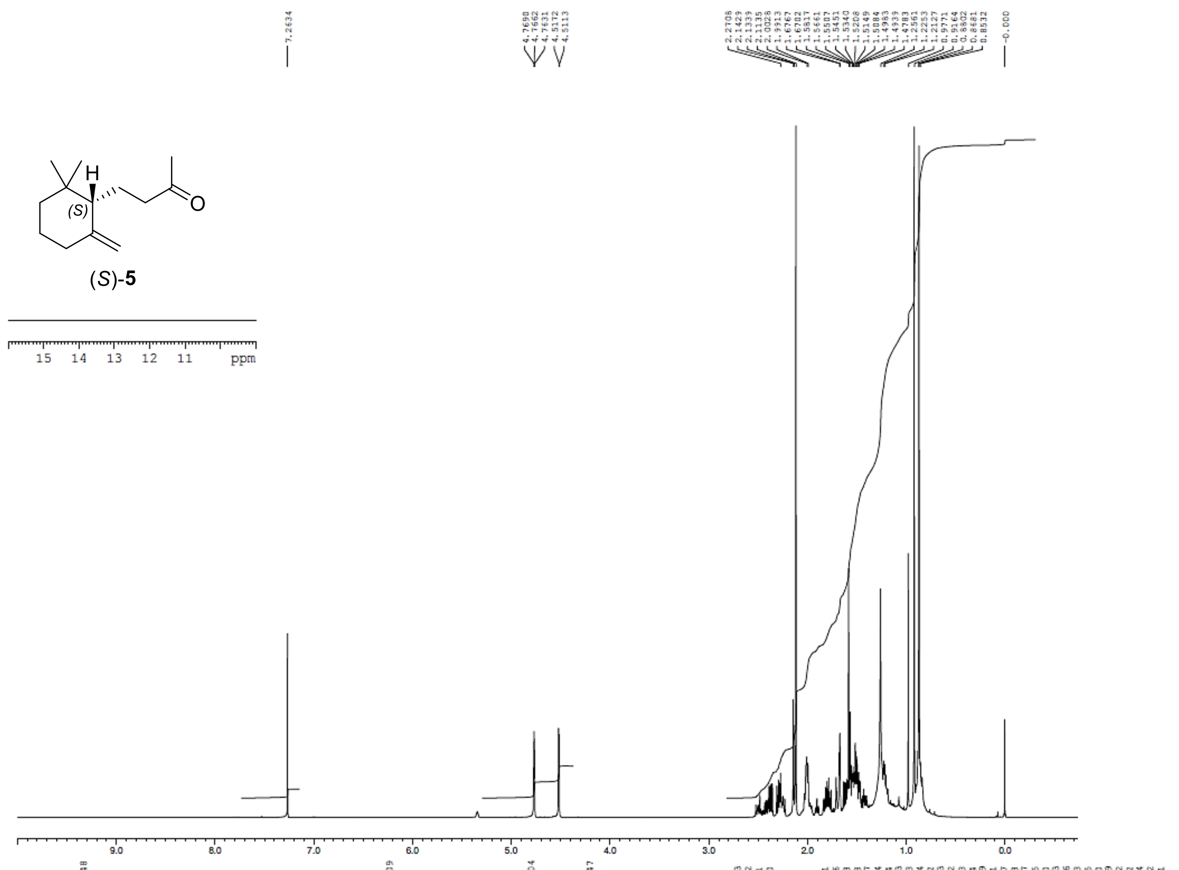

(*S*)-**5**

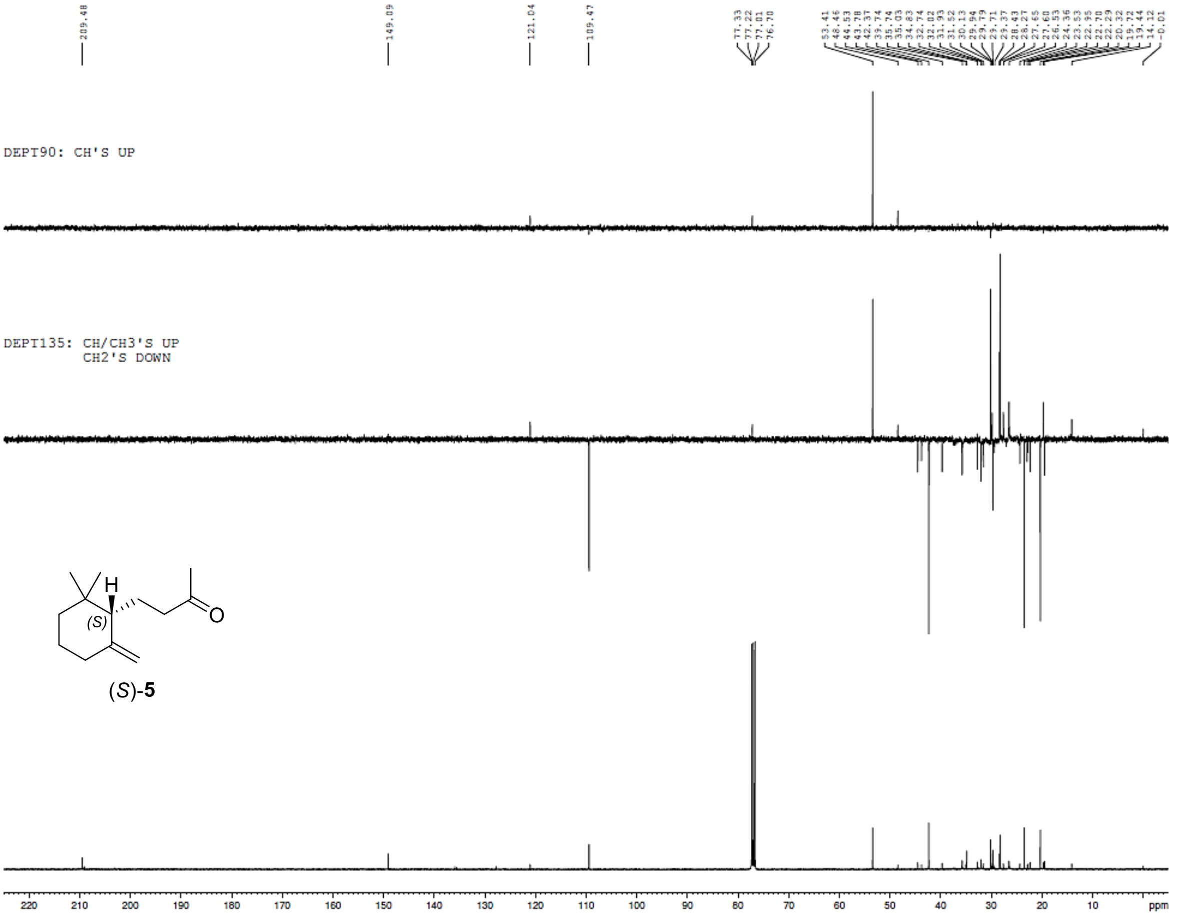

(*S*)-**5**

8. **HSQC of 4-((*R*)-2,2-dimethyl-6-methylenecyclohexyl)butan-2-yl acetate (16)**

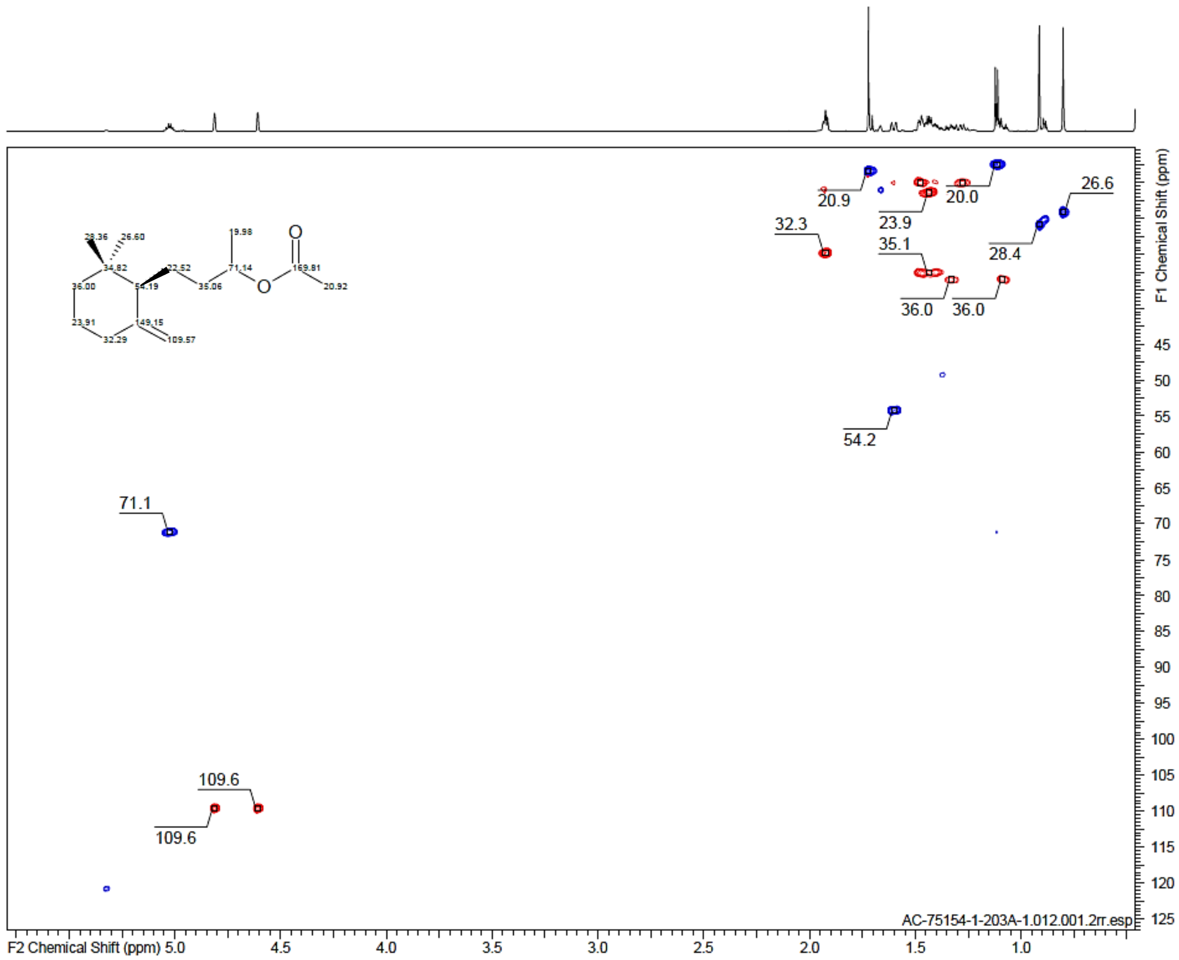

9. (4a*S*,8a*S*)-2,5,5,8a-tetramethyl-4a,5,6,7,8,8a-hexahydro-4H-chromene (*S*,*S*)-4

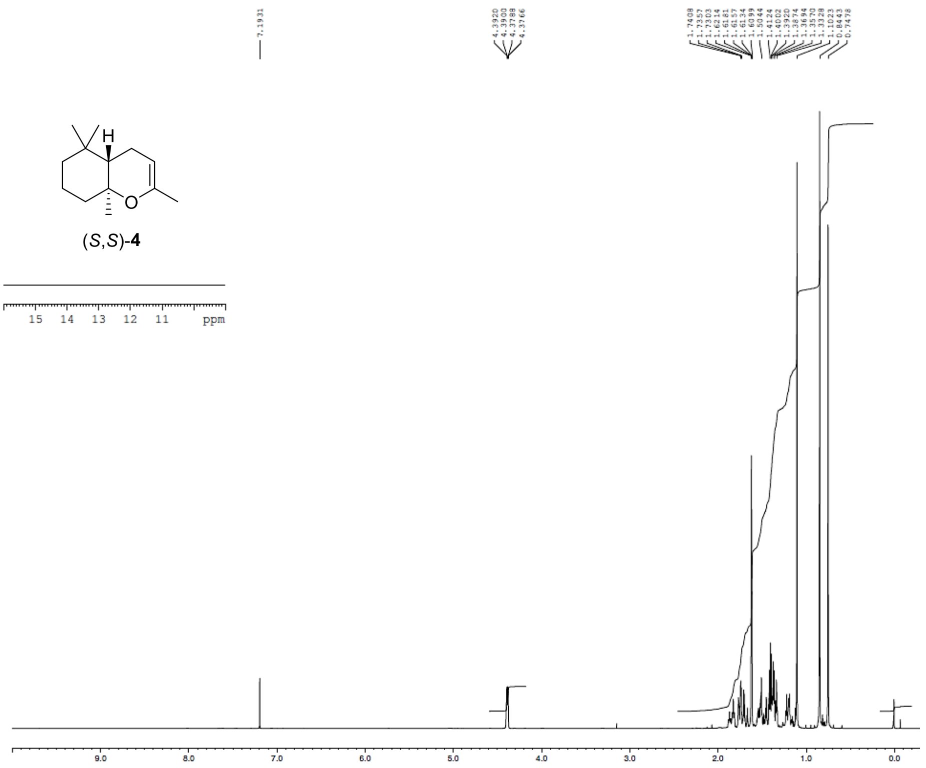

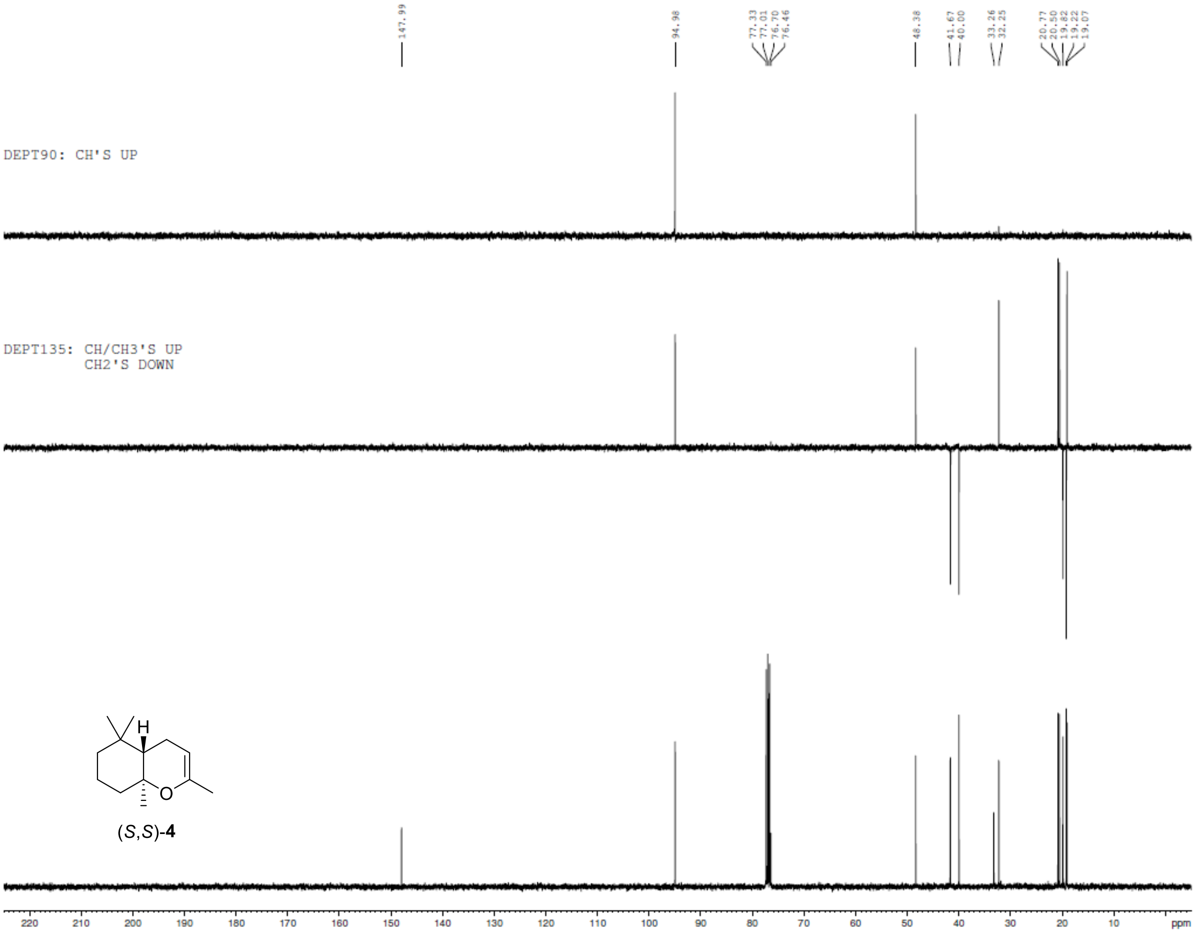

10. (4a*R*,8a*S*)-2,5,5,8a-tetramethyl-4a,5,6,7,8,8a-hexahydro-4H-chromene (*R*,*S*)-4

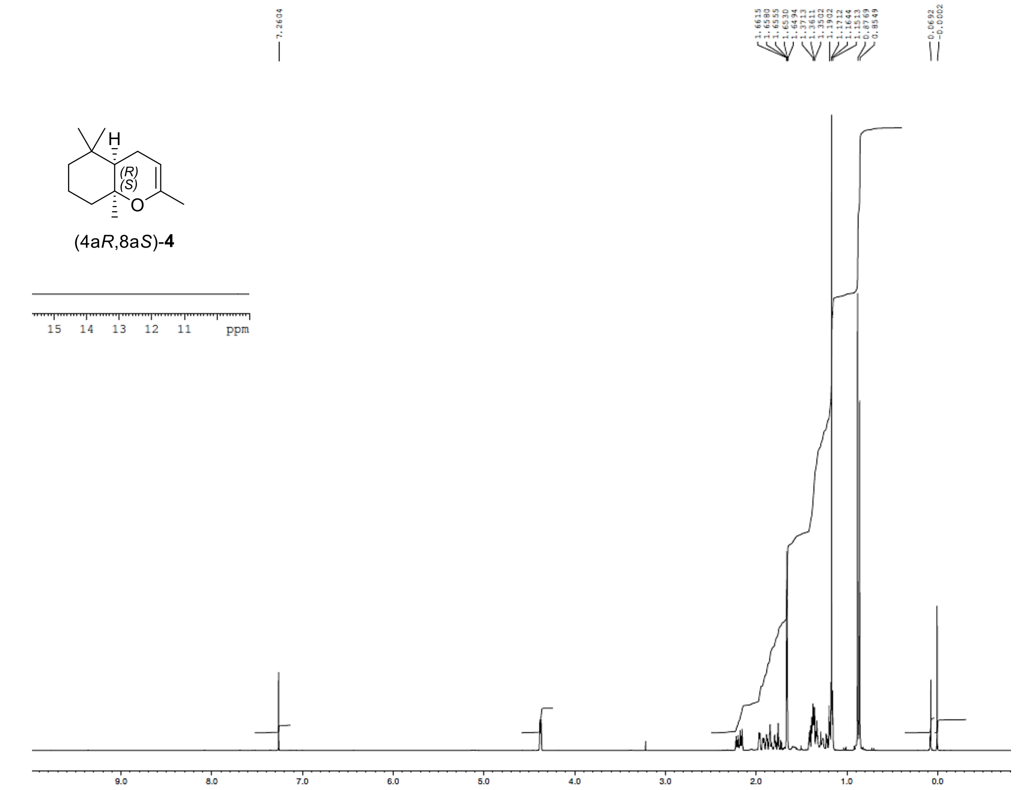

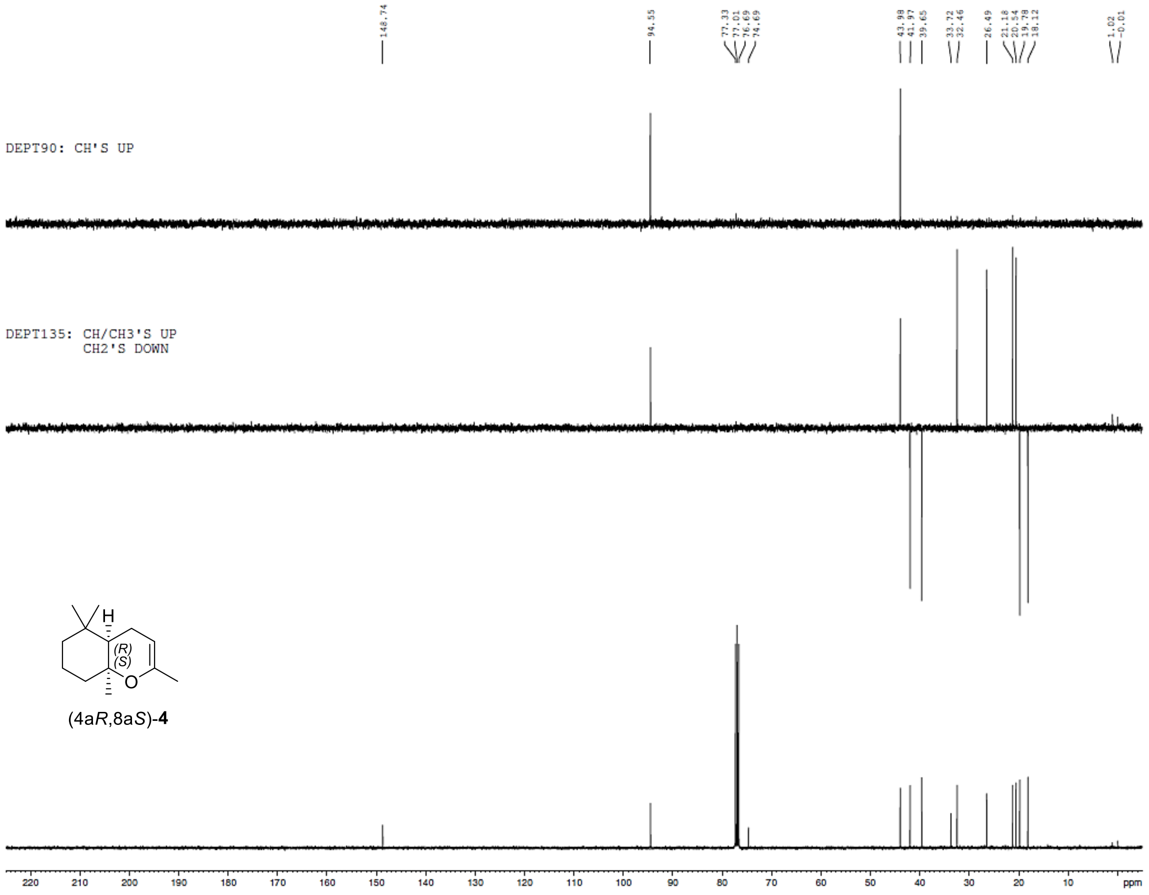

E. Chiral GC-analysis of SHC-cyclization products

1. (*R*)- and (*S*)- γ-dihydroionone (5)

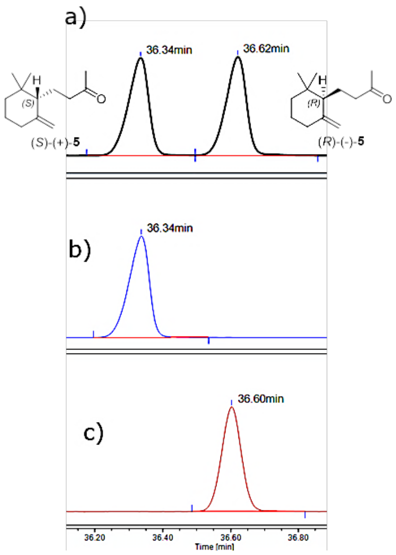

a) racemic γ-dihydroionone b) (*S*)-**5** ex *E*-tangerinol with *Aac*SHC 215G2, >99.9% e.e.   c) (*R*)-**5** ex (*E/Z*)-geranylacetone (**2**) with AciSHC_R2.3, >99 % e.e.

2. trans bicyclic enolether (*S,S)*-4

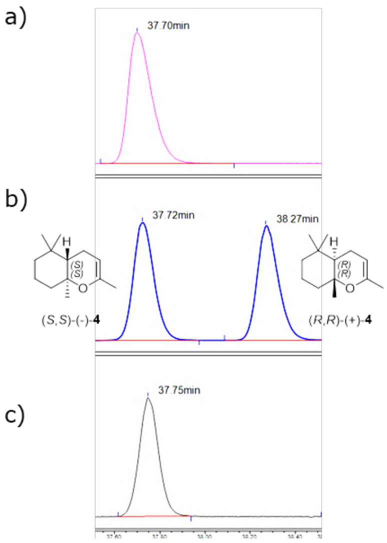

a) (*S*,*S*)-**4** ex *E*-**2** by *Aac*SHC 215G2, >99.9% e.e. b) racemic (*S*,*S*)-**4**   c) (*S*,*S*)-**4** ex (*E/Z*)-geranylacetone (**2**) by *Aci*SHC_R2.3, > 95% e.e.

3. cis bicyclic enolether (4a*R*,8a*S*-4)

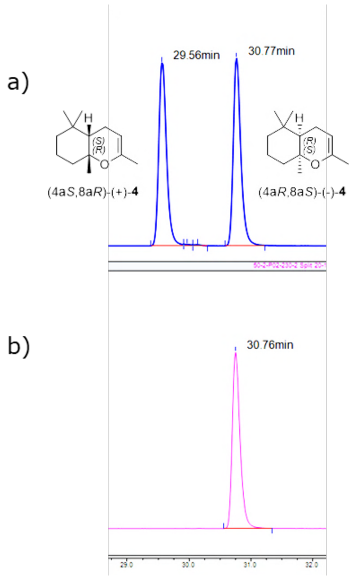

a) racemic (4a*S*,8a*R*)-**4** b) (4a*R*,8a*S*)-(-)-**4** ex *Z*-**2** by *Aac*SHC 215G2, >99.9% e.e. (absolute configuration assigned tentatively)

F. Sequences

1. pET28b(+)

ctcgagcaccaccaccaccaccactgagatccggctgctaacaaagcccgaaaggaagctgagttggctgctgccaccgctgagcaataactagcataac
cccttggggcctctaaacgggtcttgaggggttttttgctgaaaggaggaactatatccggattggcgaatgggacgcgccctgtagcggcgcattaagcgcgg
cgggtgtggtggttacgcgcagcgtgaccgctacacttgccagcgccctagcgcccgctcctttcgctttcttcccttcctttctcgccacgttcgccggctttccccgt
caagctctaaatcgggggctccctttagggttccgatttagtgctttacggcacctcgaccccaaaaaacttgattagggtgatggttcacgtagtgggccatcgcc
ctgatagacggtttttcgccctttgacgttggagtccacgttctttaatagtggactcttgttccaaactggaacaacactcaaccctatctcggtctattcttttgatttat
aagggattttgccgatttcggcctattggttaaaaaatgagctgatttaacaaaaatttaacgcgaattttaacaaaatattaacgtttacaatttcaggtggcactttt
cggggaaatgtgcgcggaacccctatttgtttatttttctaaatacattcaaatatgtatccgctcatgaattaattcttagaaaaactcatcgagcatcaaatgaaac
tgcaatttattcatatcaggattatcaataccatatttttgaaaaagccgtttctgtaatgaaggagaaaactcaccgaggcagttccataggatggcaagatcctg
gtatcggtctgcgattccgactcgtccaacatcaatacaacctattaatttcccctcgtcaaaaataaggttatcaagtgagaaatcaccatgagtgacgactgaa
tccggtgagaatggcaaaagtttatgcatttctttccagacttgttcaacaggccagccattacgctcgtcatcaaaatcactcgcatcaaccaaaccgttattcatt
cgtgattgcgcctgagcgagacgaaatacgcgatcgctgttaaaaggacaattacaaacaggaatcgaatgcaaccggcgcaggaacactgccagcgcat
caacaatattttcacctgaatcaggatattcttctaatacctggaatgctgttttcccggggatcgcagtggtgagtaaccatgcatcatcaggagtacggataaaa
tgcttgatggtcggaagaggcataaattccgtcagccagtttagtctgaccatctcatctgtaacatcattggcaacgctacctttgccatgtttcagaaacaactct
ggcgcatcgggcttcccatacaatcgatagattgtcgcacctgattgcccgacattatcgcgagcccatttatacccatataaatcagcatccatgttggaatttaat
cgcggcctagagcaagacgtttcccgttgaatatggctcataacaccccttgtattactgtttatgtaagcagacagttttattgttcatgaccaaaatcccttaacgt
gagttttcgttccactgagcgtcagaccccgtagaaaagatcaaaggatcttcttgagatcctttttttctgcgcgtaatctgctgcttgcaaacaaaaaaaccacc
gctaccagcggtggtttgtttgccggatcaagagctaccaactctttttccgaaggtaactggcttcagcagagcgcagataccaaatactgtccttctagtgtagc
cgtagttaggccaccacttcaagaactctgtagcaccgcctacatacctcgctctgctaatcctgttaccagtggctgctgccagtggcgataagtcgtgtcttacc
gggttggactcaagacgatagttaccggataaggcgcagcggtcgggctgaacggggggttcgtgcacacagcccagcttggagcgaacgacctacaccg
aactgagatacctacagcgtgagctatgagaaagcgccacgcttcccgaagggagaaaggcggacaggtatccggtaagcggcagggtcggaacagga
gagcgcacgagggagcttccagggggaaacgcctggtatctttatagtcctgtcgggtttcgccacctctgacttgagcgtcgatttttgtgatgctcgtcagggg
ggcggagcctatggaaaaacgccagcaacgcggcctttttacggttcctggccttttgctggccttttgctcacatgttctttcctgcgttatcccctgattctgtggataa
ccgtattaccgcctttgagtgagctgataccgctcgccgcagccgaacgaccgagcgcagcgagtcagtgagcgaggaagcggaagagcgcctgatgcgg
tattttctccttacgcatctgtgcggtatttcacaccgcatatatggtgcactctcagtacaatctgctctgatgccgcatagttaagccagtatacactccgctatcgct
acgtgactgggtcatggctgcgccccgacacccgccaacacccgctgacgcgccctgacgggcttgtctgctcccggcatccgcttacagacaagctgtgac
cgtctccgggagctgcatgtgtcagaggttttcaccgtcatcaccgaaacgcgcgaggcagctgcggtaaagctcatcagcgtggtcgtgaagcgattcacag
atgtctgcctgttcatccgcgtccagctcgttgagtttctccagaagcgttaatgtctggcttctgataaagcgggccatgttaagggcggttttttcctgtttggtcactg
atgcctccgtgtaagggggatttctgttcatgggggtaatgataccgatgaaacgagagaggatgctcacgatacgggttactgatgatgaacatgcccggttac
tggaacgttgtgagggtaaacaactggcggtatggatgcggcgggaccagagaaaaatcactcagggtcaatgccagcgcttcgttaatacagatgtaggtgt
tccacagggtagccagcagcatcctgcgatgcagatccggaacataatggtgcagggcgctgacttccgcgtttccagactttacgaaacacggaaaccgaa
gaccattcatgttgttgctcaggtcgcagacgttttgcagcagcagtcgcttcacgttcgctcgcgtatcggtgattcattctgctaaccagtaaggcaaccccgcc
agcctagccgggtcctcaacgacaggagcacgatcatgcgcacccgtggggccgccatgccggcgataatggcctgcttctcgccgaaacgtttggtggcgg
gaccagtgacgaaggcttgagcgagggcgtgcaagattccgaataccgcaagcgacaggccgatcatcgtcgcgctccagcgaaagcggtcctcgccga
aaatgacccagagcgctgccggcacctgtcctacgagttgcatgataaagaagacagtcataagtgcggcgacgatagtcatgccccgcgcccaccggaa
ggagctgactgggttgaaggctctcaagggcatcggtcgagatcccggtgcctaatgagtgagctaacttacattaattgcgttgcgctcactgcccgctttccag
tcgggaaacctgtcgtgccagctgcattaatgaatcggccaacgcgcggggagaggcggtttgcgtattgggcgccagggtggtttttcttttcaccagtgagac
gggcaacagctgattgcccttcaccgcctggccctgagagagttgcagcaagcggtccacgctggtttgccccagcaggcgaaaatcctgtttgatggtggtta
acggcgggatataacatgagctgtcttcggtatcgtcgtatcccactaccgagatatccgcaccaacgcgcagcccggactcggtaatggcgcgcattgcgcc
cagcgccatctgatcgttggcaaccagcatcgcagtgggaacgatgccctcattcagcatttgcatggtttgttgaaaaccggacatggcactccagtcgccttc
ccgttccgctatcggctgaatttgattgcgagtgagatatttatgccagccagccagacgcagacgcgccgagacagaacttaatgggcccgctaacagcgcg
atttgctggtgacccaatgcgaccagatgctccacgcccagtcgcgtaccgtcttcatgggagaaaataatactgttgatgggtgtctggtcagagacatcaaga
aataacgccggaacattagtgcaggcagcttccacagcaatggcatcctggtcatccagcggatagttaatgatcagcccactgacgcgttgcgcgagaaga
ttgtgcaccgccgctttacaggcttcgacgccgcttcgttctaccatcgacaccaccacgctggcacccagttgatcggcgcgagatttaatcgccgcgacaattt
gcgacggcgcgtgcagggccagactggaggtggcaacgccaatcagcaacgactgtttgcccgccagttgttgtgccacgcggttgggaatgtaattcagctc
cgccatcgccgcttccactttttcccgcgttttcgcagaaacgtggctggcctggttcaccacgcgggaaacggtctgataagagacaccggcatactctgcgac
atcgtataacgttactggtttcacattcaccaccctgaattgactctcttccgggcgctatcatgccataccgcgaaaggttttgcgccattcgatggtgtccgggatc
tcgacgctctcccttatgcgactcctgcattaggaagcagcccagtagtaggttgaggccgttgagcaccgccgccgcaaggaatggtgcatgcaaggagatg
gcgcccaacagtcccccggccacggggcctgccaccatacccacgccgaaacaagcgctcatgagcccgaagtggcgagcccgatcttccccatcggtg
atgtcggcgatataggcgccagcaaccgcacctgtggcgccggtgatgccggccacgatgcgtccggcgtagaggatcgagatctcgatcccgcgaaatta
atacgactcactataggggaattgtgagcggataacaattcccctctagaaataattttgtttaactttaagaaggagatata

2. *Bth*SHC

atgggcttattatacgaaaaagcgcatgaagaaatagcgagaagaacaactgcacttcaaacaatgcaacggcaagatggtacgtggcagttttgttttgaaggagcgccgctaacagatt
gtcatatgatttttttattaaaattattaggtagagataaagagatagaaccgtttgtaaaaagattagcatcacttcaaacaaatgaaggaacatggaaattgtatgaagatgaaatgggtggta
atttatctgctacaattcaatcttatgctgccttacttgcatcagaaaaatatacaaaagaagatgcgaatatgaagcgagcggaaatgtttataaatgagcgcggtggggtagcgcgtgctcat
tttatgacgaagtttttattagcgattcatggagaatatgaatatccttctctctttcatttgccaacaccaattatgtttctgcagaatgattcccctctcagtatatttgaattgagtagctcagcacgtat
ccatttaattccgatgatgttgtgtttaaataaaagatttcgagtagggaaaaagttattgccaaatttaaatcatattgcaggcgggggcggagaatggtttcgggaggatcggtctccagtttttc
aaacgttagtaagtgacgtgaagaaaattataacgtatccactttctttgcatcataaaggatatgaggaagtagaacgttttatgaaagagcgtattgatgaaaatggaacattatatagttac
gcaactgcctcgttttatatgatttatgctttacttgcattaggacattctattcactcaccaattattcagaaggctataatgggaatcacatcttatatatggaagatggagagagggagccatttg
caaaactctccgtcaactatatgggatacagctttactcagttatgctttgcaagaagctcaagttccgaaagcaagtaaagtgattcaaaatgcatcagcgtatttactaagaaaacagcaaa
caaagaaagtagattggagtgtacatgcaccggatctcttcccaggtggttggggcttttcggatgtgaatacgacgattccagatattgatgatacaactgctgcgttaagagcattggcgcg
aagtagagggaacgaaaatgtagacaatgcttggaagcgagcggttaattgggttaaaggattgcaaaataatgatggtggttgggggcttttgaaaaaggggtaacgagccgtatatta
gcaaatttaccaatcgaaaatgcaagtgatatgattacagatccttctacaccagatattacaggaagagtgttagaatttttcgggacgtatacgcaaaatgaattgcccgagaaacaaaaa
caaagtgcgataaattggttaacgaatgcacaagaggaaaatggatcatggtatgggaaatgggggatttgttatatatatggtacgtgggcggttatgactggtttacggtcactaggaactc
catctagcaacccatcattaaaacgagccgctttatggcttgaacatatacagcatgaagatggtggctggggagaatcttgccacagtagtgtggagaaaaggttcgttactttaccatttagt
acaccatcccaaacagcatgggcgttagatgccctcatttcttactatgataaagaaacgccagtcattcgcaaaggtatttcatatttgctctccaacccttatgtaaatgaaaaatatcctactg
gaacaggcttgccaggtgggttttatattcgttatcatagttatgctcatatatatccgttgcttactttggcacattatttaaaaaaatatagaaaataa

3. *Aac*SHC

atggcagaacagctggttgaagcccctgcctatgcacgtaccctggatcgtgcagttgaatatctgctgagctgtcagaaagatgaaggttattggtggggtccgctgctgagcaatgttacaa
tggaagcagaatatgtgctgctgtgtcatattctggatcgcgttgatcgtgatcgcatggaaaaaattcgtcgttatctgctgcatgaacagcgtgaagatggcacctgggcactgtatcctggtg
gtccgcctgatctggataccaccattgaagcctatgttgcactgaaatatatcggtatgagccgtgatgaagaaccgatgcagaaagcactgcgttttattcagagccaaggtggtattgaaag
cagccgtgtttttacccgtatgtggctggcactggttggtgaatatccgtgggaaaaagttccgatggttccgcctgaaattatgtttctgggtaaacgtatgccgctgaacatttatgaatttggtag
ctgggcacgtgcaaccgttgttgccctgagcattgtgatgagccgtcagccggtttttccgctgccggaacgtgcccgtgtgccggaactgtatgaaaccgatgttcctccgcgtcgtcgtggtg
caaaaggtggtggtggttggatttttgatgcactggaccgtgcactgcatggttatcagaaactgagcgttcatccgtttcgtcgtgcagcagaaattcgtgcactggattggctgctggaacgtc
aagccggtgatggtagttggggtggtattcagcctccgtggttttatgcactgattgccctgaaaattctggatatgacccagcatccggcatttatcaaaggttgggaaggtctggaactgtacg
gtgttgaactggattatggtggctggatgtttcaggcaagcattagtccggtttgggataccggtctggcagttctggcactgcgtgcagccggtctgcctgcagatcatgaccgtctggttaaag
caggcgaatggctgttagatcgtcagattaccgttcctggtgattgggcagttaaacgtccgaatctgaaacctggtggttttgcatttcagttcgacaatgtttattatccggatgtggatgataccg
cagttgttgtttgggcactgaataccctgcgtctgcctgatgaacgtcgtcgccgtgatgcaatgaccaaaggttttcgttggattgttggtatgcagagcagcaatggcggttggggtgcctatga
tgttgataataccagcgatctgccgaaccatattccgttttgtgattttggtgaagttaccgatccgcctagcgaagatgttaccgcacatgttctggaatgttttggcagctttggttatgatgatgcct
ggaaagttattcgtcgcgctgtggaatatctgaaacgtgaacagaaaccggatggttcatggtttggtcgctggggtgttaattatctgtatggtacaggtgcagttgttagcgcactgaaagcag
ttggtattgatacccgtgaaccgtatattcagaaagccctggattgggttgaacagcatcagaatccggacggtggctggggtgaagattgtcgtagctatgaagatcctgcgtatgcaggtaa
aggtgcaagcaccccgagccagaccgcatgggctctgatggccctgattgccggtggtcgtgccgaaagcgaagcagcacgtcgcggtgttcagtatctggttgaaacccagcgtccgga
tggcggatgggatgaaccttattacaccggcaccggttttccgggtgatttttatctgggttataccatgtatcgtcatgtgtttccgacactggccctgggtcgttataaacaggcaattgaacgtc
gctaa

4. *Bja*SHC1

atggatagcgttaatgcaaccgcacgtgaagcaaaagaaagcaaaattagcgaaagcgaaattctggaaagcagcattgcaagcgcaacccagggtgttctgggttttcagcagagtga
tggtcattgggtttttgaactggaagcagattgtaccattccggcagaatatgttctgctgcgtcattatctggcagaaccggttgataccgttctggaagcaaaaattggtaattatctgcgtcgtgt
tcagggtgcacatggtggttggcctctggttcatgatggtgaatttgatatgagcgcaagcgtgaaagcatattttgccctgaaaatgattggcgatagcattgatgctccgcacatggttcgtgcc
cgtgaagccattcatgcacgtggtggtgcaattcatagcaatgtttttacccgttttatgctggccatgtttggtattgttacctggcgtgcagttccggtgctgccgattgaaattatgctgctgccgttt
tggagcccgtttcatattaacaaaatcagctattgggcacgtaccacaatggttccgctgatggttattgcagcactgaaaccgcgtgcaaaaaatccgaaaggtgttggtattgatgaactgttt
ctgcaagatccgcgtagcattggtatgaccgcaaaagcaccgcatcagagcatggcatggtttctgctgtttcgtagcctggatgcaattctgcgtgttattgaaccgctgtttccgaaaagcctg
cgtaaacgtgcaattgataccgcactggcatttagcgaagaacgtctgaatggtgaagatggtatgggtgcaatttatccgcctatggcaaatctggtgatgatgtatgatgcactgggcaaag
atgaaaattatccgccacgtgcagttacccgtcgcggtatcgataaactgctggttattggagatgatgaagcatattgtcagccgtgtgttagtccggtttgggataccacactgaccgcacat
gcactgctggaagccggtggtgataaagcaggtccggcagcaaaacatggtctggattggctgattccgaaacaagagctggaagttaaaggtgattgggcagttaaacgtccggatgttc
gtccaggtggttgggcatttcagtataataacgcatattatccggatctggatgatacagcagttgttgttatgagcatggatcgtatgcgtcgtgaacatggtgttaccggttatgatagcgcaatt
gatcgtggtcgtgaatggattgaaggtatgcagtcagatgatggtggctgggcagcatttgatgttaataatctggaatattacctgaacaacatcccgtttagcgatcatggtgccctgctggac
cctccgaccgaagatgttaccgcacgttgtgttagcatgctggcacagctgggtgaaaccgcaaaaaccagcaaacatgttgcagatggtgttgcatatctgcgtaaaacccagcatccgg
aaggtagctggtatggtcgttggggtatgaattttatctatggcacctggtcagttctgtgtgcactgaatatggcaggcgttcgtcatgatgatccgatgattcgtaaagcagcagattggctggc
aagcattcagaataaagatggcggttggggtgaagataccgttagctatcgtctggattataaaggttgggaagcagcaccgagcaccgcaagccagaccgcatgggctctgctggcact
gatggcagcgggtgaagttgatcatccggcagttgcccgtggtgtggaatatctgattgcaacccagaatgaaaaaggtctgtgggatgaacagcgttataccgcaaccggttttccgcgtgtt
ttttacctgcgttatcatggctatagcaaatttttcccgctgtggggtttagcacgttatcgtaatctgcgcaataccaatagccgtgttgttggtgtgggtatgtaa

5. *Zmo*SHC1

atgggtattgatcgtatgaatagcctgagccgtctgctgatgaaaaaaatctttggtgcagagaaaaccagctataaaccggcaagcgataccattattggtacagataccctgaaacgtccg
aatcgtcgtccggaaccgaccgcaaaagttgataaaaccatctttaaaacgatgggcaacagcctgaataacaccctggttagcgcatgtgattggctgattggtcagcagaaaccggatg
gtcattgggttggtgcagttgaaagcaatgcaagcatggaagcagaatggtgtctggcactgtggtttctgggtttagaagatcatccgctgcgtccgcgtctgggtaatgcactgctggaaatg
cagcgtgaagatggtagctggggtgtttattttggtgccggtaatggtgatattaatgcaaccgttgaagcctatgcagcactgcgtagcctgggttatagcgcagataatccggttctgaaaaa
agcagcagcatggattgcagaaaaaggtggtctgaaaaacattcgtgtgtttacccgttattggctggcactgattggtgaatggccgtgggaaaaaaccccgaatctgcctccggaaattat
ctggtttccggataattttgtgttcagcatctataactttgcacagtgggcacgtgcaacaatggttccgattgcaattctgagcgcacgtcgtccgagccgtccgttacgtccgcaggatcgtctg
gatgaactgtttccggaaggtcgtgcacgttttgattatgaactgccgaaaaaagaaggcatcgatctttggagccagtttttcgtaccaccgatcgtggtctgcattgggttcagagcaatctgc
tgaaacgtaatagcctgcgtgaagcagcaattcgtcatgttctggaatggattattcgtcatcaggatgcagatggtggttggggtggtattcagcctccgtgggtttatggtctgatggccctgca
tggtgaaggttatcagctgtatcatccggttatggcaaaagcactgagtgcactggatgatcctggttggcgtcatgatcgtggtgaaagcagctggattcaggcaaccaatagtccggtttgg
gataccatgctggccctgatggcactgaaagatgcaaaagcagaagatcgttttacaccggaaatggataaagcagccgactggctgctggcacgtcaggttaaagttaaaggtgattgg
agcattaaactgccggatgttgaacctggtggctgggcatttgaatatgccaatgatcgttatcctgataccgatgataccgcagttgcgctgattgcactgagcagctatcgtgataaagaaga
gtggcagaaaaaaggcgttgaagatgcaattacccgtggtgttaattggttaattgcaatgcagagcgaatgtggcggttggggagcatttgataaagataataatcgtagcatcctgagcaa
aatcccgttttgtgattttggcgaaagcattgatccgcctagcgttgatgttaccgctcatgtgctggaagcatttggcaccctgggtctgagccgtgatatgccggttattcagaaagcaattgatt
atgtgcgtagcgaacaagaggcagaaggtgcctggtttggccgttggggtgtgaattatatctatggtacaggtgcagttctgcctgcactggcagcaattggtgaagatatgacccagccgt
atattaccaaagcctgcgattggctggttgcacatcagcaagaggatggcggatggggtgaaagctgtagcagctatatggaaattgatagcattggtaaaggtccgaccacaccgagcca
gaccgcatgggcactgatgggtctgattgcagcaaaccgtccggaagattatgaagcaattgcaaaaggttgccactatctgattgatcgtcaagaacaggatggttcctggaaagaagaa
gaattcaccggcaccggttttccaggttatggtgttggtcagacaattaaactggacgatccggcactgagtaaacgtctgctgcagggtgcagaactgagtcgtgcatttatgctgcgttatgat
tttatcgtcagtttttcccgattatggcactgtcacgtgcagaacgtctgatcgatctgaacaattaa

6. *Bme*TC

atgatcatcctgctgaaagaagtgcagctggaaattcagcgtcgtattgcatatctgcgtccgacacagaaaaatgatggtagctttcgttattgctttgagacaggtgttatgccggatgcatttct
gattatgctgctgcgtacctttgatctggataaagaagttctgattaaacagctgaccgaacgtattgttagcctgcagaatgaagatggtctgtggaccctgtttgatgatgaagaacataatctg
agcgcaaccattcaggcatataccgcactgctgtatagcggttattatcagaaaaacgatcgcattctgcgtaaagccgaacgctatattatcgatagcggtggtattagccgtgcacattttctg
acccgttggatgctgagcgttaatggtctgtatgaatggccgaaactgttttatctgccgctgagcctgctgctggttccgacctatgttccgctgaacttttatgaactgagcacctatgcacgcatt
cattttgttccgatgatggttgcaggcaacaaaaaattcagcctgaccagccgtcataccccgagcctgagccatctggatgttcgtgaacagaaacaagaaagcgaagaaacaacccaa
gaaagccgtgcaagtatttttctggttgatcatctgaaacagctggcaagcctgccgagctatattcataaactgggttatcaggcagcggaacgttatatgctggaacgcattgaaaaagatg
gcaccctgtatagctatgcaaccagcaccttttttatgatttatggtctgctggcactgggctacaaaaaagatagctttgtgattcagaaagccattgatggtatttgtagcctgctgagtacctgta
gcggtcatgttcatgttgaaaatagcaccagcaccgtttgggatacagccctgctgagctatgcactgcaagaagcaggcgttccgcagcaggacccgatgattaaaggtacaacccgttat
ctgaaaaaacgccagcataccaaattaggcgattggcagtttcataatccgaataccgcacctggtggttggggttttagcgatattaacaccaataatccggatctggatgataccagcgca
gcaattcgtgcactgagccgtcgtgcacagaccgataccgattatctggaaagctggcagcgtggtattaattggctgctgtcaatgcagaacaaagatggtggttttgcagcctttgaaaaaa
acaccgatagcatcctgtttacctatctgcctctggaaaatgcaaaagatgcagcaaccgatccggcaaccgcagatctgaccggtcgtgttctggaatgtctgggtaattttgcaggtatgaat
aaaagccatccgagcattaaagcagcagtgaaatggctgtttgatcatcagctggataatggtagctggtatggtcgttggggtgtttgttatatctatggcacctgggcagcaattaccggtctg
cgtgcagttggtgttagcgcaagcgatccgcgtattatcaaagccattaactggctgaaaagcatccagcaagaggatggtggctttggtgaaagctgttatagcgccagcctgaaaaaata
cgtgccgctgtcatttagcaccccgagtcagaccgcatgggcattagatgcactgatgaccatttgtccgctgaaagatcgtagcgttgaaaaaggtatcaagtttctgctgaatccgaatctga
cagaacagcagacccattatccgaccggtattggtctgcctggtcagttttatatccagtatcacagctataacgatatctttccgctgctggccctggcacattatgcaaaaaaacatagcagct
aa

7. *Gni*PNT

atgctgccgtataatcagaacagctataaagaagcactgcacggcggtcatgcagcacataatccgcctacactggaagaagcaattaaacgtagccaagaatttctgctggcacatcag
catccggaaggtttttggtggggtgatctggaatgtaatgttaccagcgcaagtcataccctgatcctgtataaaatcctgggtattgcagatcgttatccgctgcacaaatttgagaaatatctgc
gtcgtatgcagtgtagtcatggtggttgggaaatgagctttggtgatggtggttatctgagcgcaaccattgaagcatatatttgtctgcgtctgctgaatgttccgcagagcgatccggcactgca
gcgtgcactgaaaaacattctggcacgtggtggtgttaccaaagcacgtgtttttaccaaagtttgtctggcactgttaggtggttttgattgggcagcactgccgagcctgcctccgtggctgatg
ctgtttccggcatggtttccgtggaacatttatgaagcagcaagctgggcacgcggttgtgttgttccgctgattgttctgctggaaaaaaaaccggtgtttcaggttaaaccggaagtcagctttg
atgaactgtatgttgaaggtcgtgcacatgcctgtaaagccctgccgtttagcgcacatgattgggttagcaacatttttgttgcagcagatcgtgcctttaaactgatggaacgttttggtgcagttc
cgtttcgtcagtggtcaattaaagaagccaaaaaatgggtgcttgatcgccaagaagaaatgggcgattttattggttataatcctccgatgctgtattttgccgtttgtctgaaactgtggggttatg
aagttaccgatccgctgttacagcgtgccctgctggcccataaaaaactgaccgttgaaaccgaagatgaatgttggctgcagagcagccagagtccggtttgggataccgcactggttattc
cagcactggttgaaagcggtctgcctccggatcatcctgcgctgcagaaagcaggtcagtggctgttagaaaaacaaattctgaaacatggcgactgggctctgaaaacaggtggtggtcg
catgcaggatgacattggtggtggctgggcatttcagtttgttaatagctggtatccggatgtggatgatagcgcagcagttgttattgcactgaactgcattaaaatgccggatgaggatgttaa
aaatggtgcaattgcccgttgcctgaaatggattgcatttatgcagggtcgtaatggcggatgggcagcatttgatcgtgatagcaatcagcgttggatggatgcaaccccgtttagtgatattga
agcaatgctggatgttagcaccgcagatgttaccgcacgtgttctggaaatggttggtctgatgcgtctgaaacacgcagcacagcctgcaaataattcactgggtaaagcacatcgtcatatt
agcaccgaaagcattgcccgtggtgttgattatctgaccaaagaacaagaaaaagaaggctgttggtggggacgttggggtgtgaattatatctatggcacccgtggtgcactgatgggtctg
agccaggttgcagcaaaaacacataaaaaagaaattgcgcgtggtgcagcatggctggttaaagttcagaacaaaaagaacgagaagaaacagggtgcacaggatggcggttgggg
tgaagcatgttttagctatgatgatcctgcaaccaaaggtcagaatagccgtagcaccgccagccagaccggttgggcaatgcagggactgctggcagccggtgaagttctgggtcgtaaa
tatgaaatggaagcagttgaagaaggtgtgcagtttctgttagatacccagcgtaaagatggtagctggtctgaagcagaatttaccggtggtggttttccgaaacactattatctgaaatacca
ctattttgcccagcattttccgctgagcgcactggcacgttatcgtgcccgtctgctgcagctgagccgtccgaaaaatcaggcataa

8. *Aci*SHC

atgacccaggcaagcgttcgtgaagatgcaaaagcagcactggatcgtgcagttgattatctgctgagcctgcaggatgaaaaaggtttttggaaaggtgaactggaaaccaacgttacca
ttgaagccgaagatctgctgctgcgtgaatttctgggtattcgtacaccggatattaccgcagaaaccgcacgttggattcgtgcaaaacagcgtagtgatggcacctgggcaacctttatgat
ggtccgcctgatctgagcaccagcgttgaagcctatgttgcactgaaactggctggtgatgatccggcagcaccgcacatggaaaaagcagccgcatatattcgtggtgccggtggtgttga
acgtacccgtgtttttacccgtctgtggctggcactgtttggtctgtggccgtgggatgatctgccgacactgcctccggaaatgatttttctgccgagctggtttccgctgaacatttatgattggggtt
gttgggcacgtcagaccgttgtgccgctgaccattgttagcgcactgcgtccggttcgtccgattccgctgagtattgatgaaattcgtacaggtgcaccgcctccgcctcgcgatccggcatgg
accattcgtggtttttttcagcgtctggatgatttactgcgtggttatcgtcgtgttgcagatcatggtccggcacgtctgtttcgtcgtctggcaatgcgtcgtgcagcagaatggattattgcacgtca
agaggcagatggtagctggggtggtattcagcctccgtgggtttatagcctgattgcactgcatctgctgggttatccgctggatcatccggttctgcgtcgtggtctggatggtctgaatggttttac
cattcgcgaagaaacagcagatggtgcagttcgtcgcctggaagcatgtcagagtccggtttgggataccgcactggcagttacagcactgcgtgatgcaggtctgcctgccgatcatccgc
gtgttcaggcagcagcccgttggctggttggtgaagaggtgcgtgttgccggtgattgggcagtgcgtcgtccgggtctgcctcctggtggttgggcatttgaatttgccaatgataattacccgg
ataccgatgatacagcagaagttgttctggccctgcgtcgcgttcgtctggaagatgcagatcagcaggcgctggaagcagccgttcgtcgtgcaaccacctgggttattggtatgcagagca
ccgatggcggttggggtgcatttgatgcagataatacccgtgaactggtgctgcgtctgccgttttgtgattttggtgccgttattgatccgcctagcgcagatgttaccgcacatattgttgaaatgc
tggcagccctgggtatgcgtgatcatcctgccaccgttgcgggtgttcgttggctgctggcacatcaagaacctgatggtagttggtttggtcgttggggagcaaatcatatttatggtacgggtgc
agttgttccggcactgattgcagccggtgttagtccggatacaccgcctattcgtcgcgcaattcgctggctggaagaacatcagaatccggatggtggatggggtgaagatttacgtagctat
accgatcctgcactgtgggttggtcgtggtgttagcaccgcaagccagaccgcatgggcactgctggcattactggcagcgggtgaagaagcaagtccggcagttgatcgtggcgttcggtg
gctggttaccacacagcagcctgatggtggctgggatgagccgcattacaccggtacaggttttccgggtgatttctatattaactatcatctgtatcgcctggtgtttccgattagtgcactgggtc
gttatgttaatcgt

9. *Gvu*SHC

atggttgcagatgaacgtagcgttctgattgatgcactgaaacagagccaggcagcagatggtagctggcgttttccgtttgaaaccggtattagcaccgatgcctatatgattattctgctgcgt
accctggaaattaatgatgaaccgctgattcaggcactggttgaacgtattgaaagccgtcaagaggcaaatggtgcatggaaactgtttgccgatgaaggtgatggtaatgttaccgcaac
caccgaagcatactatgcactgctgtatagcggttatcgtaaaaaaaccgatccgcacatgcagaaagcaaaaatgcgtattctggaaatgggtggtctggaaaatgttcacctgtttaccaa
agttatgctggcactgacaggtcagcatccgtggcctcgtcgttttccgctgccgctggcatttttctgctgccaccgagctttccgctgaatatgtatgatctgagcgtttatggtcgtgccaatatg
attccgctgctggttgcagccgaacgtcgttatagccgtaaaaccgcaaaaagtccggatctgagtgatctggcagcaagccgtggtggttggcgtctgccggaaaatcgtgcactgtggtca
tatatcaaacgtgcactgaccggttttccggatgaactgcatgatgcagcaaaacagcgtgcagttcgttatatgtttggtcatattgaaccggatggcaccctgtatagctattttagcagcacct
ttctgtttatctttgccctgctggccctgggttatccgaaagatgatctgcatattgcacgtgccgttcgtggtctgcgtagcctgcgtaccgaaattgatggtcatacccacatgcagtataccaccg
caagcgtttggaataccgcactggcaagctatgccctgcaagaagcaggcgttccgagcaccgatcgtaccattgaaaaagcaaatcgttatctgctgagccgtcagcatattcgttatggtg
attgggcagttcataatccgcatagcctgcctggtggctggggttttagtgatgttaataccatgaatccggatgtggatgataccacagcagcactgcgtgcaattcgtcgtgcagcagcaaa
agaaaccgcatttcgtcatgcatgggatcgtgcgaatcgttggctgtttagcatgcagaataacgatggtggttttgcagcctttgaaaaaaatgtgggtaaacgcttttggcgctacctgctgatt
gaaggtgcagaatttctgctgatggacccgagtaccgcagatctgaccggtcgcaccctggaatatttcggcacctttgcaggtctgaccaaagatcatccggcagttgcccgtgcagttgatt
ggctgctggatcatcaagaggccgatggtagttggtatggtcgttggggtatttgttatgtttatggcacctgggcagcagttaccggtctgagcgcagttggtgttagtgccgatcatcctgcaat
gcaaaaagccgttcactggctgctgagcattcagaatgcagatggcggttggggtgaaagctgtaaaagtgatggtgcaaaaacctatgttccgctgggtgcaagcacaccggttcatacc
gcatgggcattagatgccctgattgcagccgcagaacgtccgacaccggaaatgaaagccggtgttcgtgccctggttcgtatgctgcaccatcctgattggaccgcaagctatccggttggt
caaggtatggcaggcgcattctatattcattatcatagctatcgctacatcttccctctgctggcgctggcacattatgaacagaaattttgtctgatcgccaattaa

10. *Mfu*SHC

atgcatagcggtcgtctgtttctgaaaaaagaaaatgaagtgggcgacaacaaaaaactgcatagcgttccgctgagcctggttgaagaaaccctgaattttccgcagaaagtggaaaaa
accatcaaaaaagcacagcgttatctgctgagcatccagaaagaagatggtcattgggttggtgaactgtttgttgatgttaccctggcatgtgattgtatccatctgatgcattggcgtggcaaa
atcgattacaaaaaacagaaacgcctggtgaaacatattctggatcgtcagctgccggatggtggttggaacatttatcctggtggtccgagcgaagttaatgcaaccgttaaagcatattttgc
cctgaaactggcaggttttagtccggatgaaccgctgatggcaaaagcacgtagcaccattctgcgtttaggtggtattccgaaatgtatgacctataccaaactgggtttagcactgctgggtg
tttatccgtgggatcgtctgccggttattccgcctgaaattattctgtttccgaactggtttccgttcaacctgtatgaaattagcgcatggtcacgcaccatgctggtgccgctgagcattattcatcat
tttaaaccgacacgtatcctgccggaaaaattacagctgcatgaactgttcccgtatggcaccgaacgtggtaaatttagctggctgaaaaagggtgcaaaatacctgagcaaagagggcc
tgtttctggcctgtgataaatttctgcagtattgggataaaaccagcctgaaaccgtttcgtaaaatggcaattgaaaaagccgagaaatggattctggaacgtattgcagcaggtagtgatggt
ctgggtgcaatctttccggcaatgcattatgcaattatggcactgattgcactgggttataccgaagataatccgattctgaagaaagccatcaccgattttgaaagcctggaagttgatgatcag
aaaaacgatgatctgcgtattcagccgtgtctgagtccgctgtgggataccgcaattggtctggttgcactggcagaaagcggttatgaacgtaatgcaccgcagcttaaaaaagcagcaca
ttggattattaaccgcgagattcgtattaaaggcgattggtatgttcgtaatccgcatccggaagcaagcggttgggcatttgaatataacaacatgtattatccggatgtggatgacacactgat
ggttctgctggcactgcgtctgattgatattgatgacaaaatcaaaaaagaagaggtgatgcagcgtgcactgcgttgggttattagctttcagtgtgaaaatggtggctgggcagcatttgata
aaaacgtgtacaaaaagtggctggaagatattccgtttgcagatcataatgcaattctggaccctccgtgtagcgatattaccgcacgtgcactggaactgttcggtaaaatgggtattcgcaa
aaacgaaaagtttgtgcagaaagcaatccgctatctgaaagaaacccaagaaagtgatggtagctggatgggtcgttggggtgtgaattatatctatggcacctggcaggccctgcgtggtc
tgcaggcaattggtgaagatatgaatcaagagtggattctgcgtgcacgtgattggctggaaagctgtcagaatgaggatggcggttggggtgaaacaccggcaagctatgataatccgca
gctgaaaggtaaaggtccgagcaccgcaagccagaccgcatgggcaattagcggtattatggcatgtggtgatatttttcgtccgagcattacccgtggtatcaaatatctgtgtgaacgtcag
ctgagtgatggttcatgggcagaagaatttctgaccggcaccggttttccgggtgtttttatctgaaatatgacatgtatcgcaatgcatggcctctgctggttattggtgaatattatcgtcagtatc
agcaggcaaaagaacgtgcaacctattgggttgatgcaaccctgggttgtatggaaaaacgtctgagcgcagtttaa

11. *Tel*SHC

atgccgaccagcctggcaaccgcaattgatccgaaacagctgcagcaggcaattcgtgcaagccaggattttctgtttagccagcagtatgccgaaggttattggtgggcagaactggaaa
gcaatgttaccatgaccgcagaagttattctgctgcataaaatctggggcaccgaacagcgtctgccgctggcaaaagcagaacagtatctgcgtaatcatcagcgtgatcatggtggttgg
gaactgttttatggtgatggtggtgatctgagcaccagcgttgaagcatatatgggtctgcgtctgctgggtgttccggaaaccgatccggcactggttaaagcacgtcagtttattctggcacgtg
gtggtattagcaaaacccgtatttttaccaaactgcatctggcactgattggttgttatgattggcgtggtattccgagcctgcctccgtggattatgctgctgccggaaggtagcccgtttaccattta
tgaaatgagcagctgggcacgtagcagcaccgttccgctgctgattgttatggatcgtaaaccggtgtatggtatggaccctccgattacactggatgaactgtatagcgaaggtcgtgcaaat
gttgtgtgggaactgcctcgtcaaggtgactggcgtgatgtttttattggtctggatcgtgtgttcaaactgttcgaaaccctgaacattcatccgctgcgtgaacagggtctgaaagcagcagaa
gaatgggttttagaacgtcaagaagcatcaggcgattggggtggcattattccggcaatgctgaatagcctgctggcactgcgtgcactggactatgcagttgatgatccgattgttcagcgtgg
tatggcagcagttgatcgttttgcaattgaaaccgaaaccgaatatcgtgttcagccgtgtgttagtccggtttgggataccgcactggtgatgcgtgcaatggttgatagcggtgttgcaccggat
catcctgcgctggtgaaagccggtgaatggctgctgagcaaacaaattctggattatggcgattggcacatcaaaaacaaaaaaggtcgtcctggtggctgggcatttgaatttgaaaatcgt
ttttatccggatgtggatgataccgcagttgttgttatggcactgcatgcggttaccctgccgaatgaaaatctgaaacgtcgtgccattgaacgtgcagttgcatggattgcaagtatgcagtgtc
gtccaggcggttgggcagcatttgatgttgataatgatcaggattggctgaacggtattccgtatggcgatctgaaagccatgatcgatccgaataccgcagatgtgaccgcacgtgttctgga
aatggttggtcgttgtcagctggcatttgatcgtgttgccctggatcgcgcactggcatatctgcgcaatgaacaagaaccggaaggttgttggtttggtcgctggggtgttaattatctgtatggca
ccagcggtgttctgaccgcactgagcctggttgcaccgcgttatgatcgttggcgtattcgtcgtgcagccgaatggctgatgcaatgtcagaatgcagatggtggatggggtgaaacctgttg
gagctatcatgatccgagtctgaaaggtaaaggtgatagcaccgcaagccagaccgcatgggcaattatcggtctgctggcagccggtgatgcaaccggtgattatgcaaccgaagcaat
tgaacgcggtattgcctatctgctggaaacccagcgtccggatggcacctggcatgaagattatttcaccggcaccggttttccgtgtcatttctatctgaaatatcactactatcagcagcattttc
cgctgacagcactgggtcgttatgcacgctggcgtaatctgctggccacctaa

12. *Afu*SHC

atgctgggtgcaattcgtgaaccgcctattgatgttcagattgcactgcatagccgtgatgataatcagacaggtctggttctgcgtggcacccgtcgtaccgttgatcgtgttctgaaaggtctgt
gtagcagcccgtgtttttttgtagcgttagcctgacaatggcaaccctgaccaccacaatggccaccaccgcaacgatggcaaccaccgaagcaagcaaaccgctggaagcacaggca
cgtaccgcactgaccaaagcaaccaattatgcatgggaaatctttagcaatcgtcattggtgtggtgaactggaaagcaatgttaccgttacctgtgaacacatcttctttctgtatgtgctgtacc
agcatattgatcctggtgaaggtagccagtatcgtcagtggctgctgagccagcagaatagtgatggtagctggggtattgcaccgaattatccgggtgatattagcaccagcgcagaagcat
atctggcactgcgtattattggtatgagcaccgatagtccggaactgtatcgtgcacgtacctttattcgtgcagccggtggtctgagcaaaatgcgtatgtttacccgtatctttttgccgaatttgg
tctggtgccgtggaccgcaattccgcagctgcctgcagaatttattctggttccggcacattttccgattagcatttatcgtctggcaagctgggcacgtagcaatgttgttccgctgctgattattgc
acatcatcgtccgctgtatccgctgccgaatggtctgcataaacagaatccgtttctggatgaactgtggttagatccggcaacaaaaccgctgccttatggtagcagcgatccgaccgatccg
gttgcatttgtttttaccattctggataaagccctgagctatttaggtggtctgcgtcgtagcccgacacgtggttatgcacgtcgtcgttgtgttcagtggattctgcagcatcaagaaaaagccggt
gattgggcaggtattattccgcctatgcatgccggtattaaagcactgctgctggaaggttataaactgcatgatgaaccgattcagctgggtttagcagcaattgaacgttttacctgggcagat
aatcgtggtaaacgtctgcagtgttgtattagtccggtttgggataccgttctgatgattcgtgcactgcaggatacaccggcaagcctgggtattaaacttgatccgcgtattgcagatgcactgg
catggaccgcagaaaatcagcatcgtggtccggaaggtgattggcgtgtgtataaaccgaacattccggttggtggttgggcatttgaatatcataatacatggtatccggacatcgatgatac
cgcagcagccgttctggcatttctgacccatgatcctgcaaccgcacgtagccgtctggttcgtgatgcagttctgtggattgttggtatgcagaatgcagatggtggctgggcagcatttgatcat
gaaaacaatcagctgttcctgaacaaaatcccgtttagcgatatggaaagcctgtgtgatccgagcacaccggatgttaccggtcgtaccattgaatgtctgggtatgctgcgtgatctgctgat
gcgtccggcagaaaatgccgaaaatggtgagaaatatggttatccggatggcgaaggtgatgcagcagcagacgcacatctgctgcagattattaacaccgcatgtgcacgtgccattcc
gtatctgattcgtagccaagaagcaaccggcacctggtatggtcgttgggcagttaattatgtttatggcacctgtctggtgctgtgtggtctgcagtatttcaaacatgatccgaaatttgcaccgg
aaattcaggcaatggcagcacgtgcagttaaatggctgaaacaggttcagaattccgatggcggttggggtgaaagcctgctgagctatcgcgaaccgtggcgtgcaggttgtggtccgtca
acaccgagccagaccgcatgggcactgatgggtattctgaccgtgtgtggtggtgaagatcgtagcgttcagcgtggtgttcgtcatctggttgatacccaggatgataccctgagccaaggt
gatggtggtgcagccgcatggacagaacgtgaatttaccatccgcgaaccgctgcatgaagcaagccagcgtattggtagcgattaa

13. *Apa*SHC

atgaatatggcaagccgtttcagcctgaaaaaaatcctgcgtagcggtagcgatacccagggcaccaatgttaataccctgattcagagcggcaccagcgatattgttcgtcagaaaccgg
caccgcaagaaccggcagatctgagcgcactgaaagcaatgggtaatagcctgacacataccctgagcagcgcatgtgaatggctgatgaaacagcagaaacctgatggtcattgggtt
ggtagcgtgggtagcaatgcaagcatggaagcagaatggtgtctggcactgtggtttctgggtttagaagatcatccgctgcgtccgcgtctgggtaaagcactgctggaaatgcagcgtccg
gatggtagctggggcacctattatggtgcaggtagcggtgatattaatgcaaccgttgaaagctatgcagcactgcgtagcctgggttatgcagaagatgatccggcagttagcaaagcagc
agcatggattattagcaaaggtggtctgaaaaatgtgcgtgtgtttacccgttattggctggcactgattggtgaatggccgtgggaaaaaaccccgaatctgcctccggaaattatctggtttcc
ggataattttgtgttcagcatctataactttgcacagtgggcacgtgcaaccatgatgccgctggcaattctgagtgcacgtcgtccgagccgtccgttacgtccgcaggatcgtctggatgccct
gtttcctggtggtcgtgcaaattttgattatgaactgccgaccaaagaaggtcgcgacgttattgcagatttttccgtctggcagataaaggtctgcattggctgcagagcagctttctgaaacgtg
caccgagccgtgaagcagcaatcaaatatgttctggaatggattatctggcatcaggatgcagatggtggttggggtggtattcagcctccgtgggttatggtctgatggccctgcatggtgaa
ggttatcagtttcatcatccggttatggcaaaagcactggatgcactgaatgatcctggttggcgtcatgataaaggtgatgcaagctggattcaggcaaccaatagtccggtttgggataccat
gctgagcctgatggcattacatgatgcaaatgccgaagaacgttttacaccggaaatggataaagccctggactggctgctgagccgtcaggttcgtgtgaaaggtgattggagcgttaaact
gccgaataccgaacctggtggctgggcatttgaatatgccaatgatcgttatcctgataccgatgataccgcagttgccctgattgccattgcaagctgtcgtaatcgtccggaatggcaggca
aaaggtgttgaagaggcaattggtcgtggtgttcgttggctggttgcaatgcagagtagctgtggcggttggggagcatttgataaagataacaataaaagcatcctggcgaaaatcccgtttt
gcgattttggtgaagcgctggaccctccgagcgttgatgttaccgctcatgtgctggaagcatttggtctgctgggtctgcctcgtgatctgccgtgtattcagcgtggtctggcatatattcgtaaag
aacaggacccgaccggtccgtggtttggccgttggggtgttaattatctgtatggtacaggtgcagttctgcctgcactggcagcactgggtgaagatatgacccagcccgtatattagtaaagc
ctgcgattggctgattaactgccagcaagaaaatggcggatggggtgaaagctgtgcaagctatatggaagttagcagcattggtcatggtgcaaccacaccgagccagaccgcatgggc
actgatgggtctgattgcagcaaatcgtcctcaggattatgaagcaattgcaaaaggttgccgctatctgattgatctgcaagaagaggacggtagctggaatgaagaagaattcaccggta

caggttttccaggttatggtgttggtcagacaattaaactggatgatccagccattagtaaacgtctgatgcagggtgcagaactgagccgtgcatttatgctgcgttatgatctgtatcgtcagctg
tttccgattattgcactgagtcgtgcaagccgtctgatcaaactgggtaattaa

14. *Bam*SHC2

atgattcgtcgcatgaataaaagcggtccgagtccgtggtcagcactggatgcagcaattgcacgtggtcgtgatgcactgatgcgtctgcagcagccggatggtagctggtgttttgaactgg
aaagtgatgcaaccattaccgcagaatatatcctgatgatgcacttcatggataaaatcgatgatgcccgtcaagaaaaaatggcacgttatctgcgtgcaattcagcgtctggatacacatg
gtggttgggatctgtatgttgatggtgatccggatgtgagctgtagcgttaaagcatattttgcactgaaagcagccggtgatagcgaacatgcaccgcacatggttcgtgcacgtgatgcaatt
ctggaattaggtggtgcagcacgtagcaatgtttttacccgtattctgctggcaacctttggtcaggttccgtggcgtgcaaccccgtttatgccgattgaatttgttctgtttccgaaatgggttccgat
cagcatgtataaagttgcatattgggcacgtaccacaatggttccgctgctggttctgtgtagcctgaaagcacgtgcccgtaatccgcgtaatattgcaattccggaactgtttgttacccctccg
gatcaagaacgtcagtattttcctccggcacgtggtatgcgtcgtgcatttctggcactggatcgtgttgttcgtcatgttgaaccgctgctgccgaaacgtctgcgtcagcgtgccattcgtcatgc
acaggcatggtgtgcagaacgtatgaatggtgaagatggtttaggtggtatttttccgcctatcgtttatagctatcagatgatggatgttctgggttatccggatgatcacccgctgcgtcgtgattg
tgaaaatgcactggaaaaactgctggtgacccgtccggatggttcaatgtattgtcagccgtgtctgagtccggtttgggataccgcatggtcaacaatggccctggaacaggcacgcggtgt
tgccgttccggaagcgggtgcaccggcaagtgccctggatgaactggatgcccgtattgcccgtgcctatgattggctggccgaacgtcaggttaatgatctgcgtggcgattggattgaaaa
cgcaccggcagatacccagcctggtggctgggcatttcagtatgcaaatccgtattatccggacattgatgatagcgcagttgttaccgcaatgctggatcgtcgtggtcgtacccatcgtaatg
cagatggtagccatccgtatgcagcccgtgttgcacgtgcgctggattggatgcgtggtctgcagagtcgtaatggtggttttgcagcatttgatgcagattgtgatcgtctgtatctgaatgccatt
ccgtttgcagatcatggtgcactgctggaccctccgaccgaagatgttagcggtcgtgttctgctgtgttttggtgttaccaaacgtgcagatgatcgtgcaagcctggcacgtgcgattgattatgt
taaacgtacccagcagcctgacggttcatggtggggtcgttggggcaccaattatctgtatggcacctggtcagttctggcaggtctggccctggcaggcgaagatccgagccagccgtatat
tgcgcgtgcactggcatggctgcgtgcccgtcagcatgccgatggcggttggggtgaaaccaatgatagttatattgatccggcactggcaggcaccaatgccggtgaaagcaccagcaat
tgtaccgcctgggctctgctggcccagatggcatttggtgatggtgaaagcgaaagcgtgcgtcgtggtattgcatatctgcagagcgttcagcaggatgatggtttttggtggcatcgtagccat
aatgcaccgggttttccgcgtattttctatctgaaatatcatggctataccgcctattttccgctgtgggcattagcccgttatcgtcgtttagccggtggtgttagcgcagcaggcgcacatgcagtt
ccggcaagtaccggtgcagatgcagcactggcctaa

15. *Mca*SHC

atgctgcgtgaagcaaccgcaattagcaatctggaaccgcctctgaccgcaagctatgttgaaagtccgctggatgcagcaattcgtcaggcaaaagatcgtctgctgagcctgcagcatct
ggaaggttattgggtttttgaactggaagcagattgtaccattccggcagaatatatcctgatgatgcactttatggatgaaattgatgcagcactgcaggccaaaattgcaaattatctgcgtag
ccatcagagcgcagatggtagctatccgctgtttcgtggtggtgccggtgatattagctgtaccgttaaagtttattacgccctgaaactggcaggcgatagcattgatgcaccgcacatgaaa
aaagcacgtgaatggattctggcacaaggtggtgcagcacgtagcaatgtttttacccgtattatgctggcaatgtttgagcagattccgtggcgtggtattccgtttattccggttgaaattatgct
gctgccgaaatggtttccgtttcatctggataaagttagctattggagccgtaccgttatggtgccgctgtttattctgtgtagccataaagttaccgcacgtaatccgagccgtattcatgttcgtga
actgtttaccgttgatccgcagaaagaacgccattattttgatcatgttaaaacaccgctgggcaaagcaattctggccctggaacgttttggtcgtatgctggaacctctgattccgaaagcagtt
cgtaaaaaagcaacccagaaagcctttgattggtttacagcacgtctgaatggtgttgatggtctgggtgcaatttttccggcaatggttaatgcctatgaagcactggattttctgggtgttcctcc
ggatgatgaacgtcgtcgtctggcacgtgaaagcattgatcgcctgctggtttttcagggtgatagcgtttattgtcagccgtgtgttagcccgatttgggataccgcactgaccagtctgaccctg
caagaagttgcacgtcatacagccgatctgcgtctggatgcggcactgagcaaaggtctgaaatggctggcaagcaaacaaatcgataaagatgcacctggtgattggcgtgttaatcgtg
caggtctggaaggcggtggttgggcatttcagtttggtaatgattattatccggatgtggatgatagcgcagttgttgcacatgcactgctgggtagcgaagatccgagctttgatgataatctgcg
tcgtgcagcaaattggattgcaggtatgcagagccgtaatggtggttttggtgcatttgatgcagataacacctattactatctgaacagcattccgtttgcagatcatggtgccctgctggaccct
ccgaccgcagatgttagcgcacgttgcgcaatgtttctggcacgctgggttaatcgtcagccggaactgcgtccggttctggaacgtaccattgattatttacgtcgtgaacaagaagcagacg
gtagctggtttggtcgttggggcaccaattatatctatggcacctggtcagttctgctggcgtatgaagcagccggtgttccgaatgatgatccgagcgttcgtcgtgccgttgcatggctgaaaa
gcattcagcgtgaagatggtggctggggtgaagataactttagctatcatgatccgtcatatcgtggtcgttttcataccagcaccgcgtttcagaccggttttgcactgattgccctgatggcagc
gggtgaagcaggtagtccggaagttcaggcaggcgttgattatctgctgcgtcagcagcgtccggatggtttttggaatgatgaatgttttaccgcaccgggttttccgcgtgtttttatctgaaat
atcacggctacgacaaatttttcccgctgtgggcattagcacgttatcgtaatgaacgttatgcactggcataa

16. *Sfu*SHC

atgcgtcgtctggatacctttccgcctgaaattccgaccggtagccgtgataaaccgcctagcggtgaagaacatagctgtagcacaccggcagaaccgctgcgtagccgtctggatgaag
gtattctgcgtgcagttgattggctggtttgtgatcagcatcctgatggtttttgggcaggtatgctgcagagcaatagctgtatggaagcagaatgggttttagccatgcattttctgggtattgatga
tgatccgaaatatgatggtgtgattcgtgcaattctgggtgaacagcgtgcagatggtagctggggtgtttttcataaagcaccgaatggtgatatcaataccaccgttgaatgttatgcagcact
gcgtgcaagcggtctggcaccggaaagcgcaccgctgagcagcgcacgtgaatggattctggcaggcggtggtctggcaaatattcgtaattttaccaaatattggctggccctgattggtg
aatggccgtgggaaggcaccccgaccattcctccggaactgatctttttccgcctcgtatgccgctgaacatttatcattttgcaagctgggcacgtagcaccattgttccgctgagtattctgag
cgcacgtcgtccggttcgtccgctgccggaagatcgtcgcctggatgaactgtttccgcagggtcgtagcgcatttgattttcgtctgcctcgtaaagatggttggctgagctgggaaggtttttttc
atgtttgcgatcgtatcctgcgtctgtatgcacgtacccgtcgtgcaccgtttcgtgaaaccgcaattcgtgtttgtctggaatggattattcgtcgtcaagaaaccgatggtgcatggtcaggtattc
agcctccgtggatttatgcactgctggcactgcatgccgaaggttatggtctggatcatccgatcctgcgtgccggtctgcgtgcctttgatagccattggagctatgaacgtgatggtggtatttat
ctgcaggcaagcgaaagtccggtttgggataccgttctgagcctgcgtgcactggcagattgtggcgaagaacgtaaagcaagcgttagcattgcaagcgcactggaatggctgctgaatc
gtcagattagcgttcctggtgattgggcagttcgtgttccgagcgttccgtgtggtggttgggcatttcagcgtgcaaatagcttttatccggatgttgatgataccgcagttgcaattgaagttctggc
acgtctgcgtccgtttaccgcaaatcagagcgcagttgatcgtgccattcgtagtgcacgtgattgggtgttagcaatgcagtgtagcaatggtggctgggcagcatttgatcgtgataatgatttt
aaactggtgaccaagattccgttttgcgattttggtgaactgctggacccaccgagcgttgatgtgaccgcacatgttattgaagccctggcagcattaggttgggatatgaccagccgtgaaat
tgaagcagcagttagctttattcgccgtgaacaagaagccgaaggtagctggtttggtcgttggggtgttaatcatatttatggcaccgcaaccgttctgcctgcgctgcgtgccattggtgaaga
tatgagcagtgcctatgtgctgcgtgcggcagactggctggcaagccgtcagaatgcagatggcggttggggtgaaacaccggcaagctatatggatgatagtctgcgtggtgttggtgaaa

gcaccgcatcacagaccgcatgggcaattatgggtttagttgcagttggtagcggtgcacatgatgatacagttcgtcgcggtattgattttctgctgtttgcacagcatggtggcacctgggaag
aaccgcagtataccggcaccggttttccgggttatagcgttggtgaacgtattcgtctgcgtgatatgggtgcaagcctgaaacagggcaccgaactgcagcgtgcatttatgattaattataac
ctgtaccgccactactttccgctgatggcactgggtcgtgcacgttatcatctgcagctgcgtcgttcagcacgcgaaggtggtaatggtgaaacaaccccgaatggtagcgcactgtaa

17. *Ttu*SHC

atggaaatccaggatgaagttgatctgctggaaccgcaagaaagcctgaccgcaagcgcagatagcgcagttgatcgtgcactgttttggctgctggatgcacagtatgaagatggttattg
ggcaggtattctggaaagcaatgcatgtatggaagcagaatggctgctgtgttttcatgttctgggtattgcaaatcatccgatgagccgtggtctggttcagggtctgctgcagcgtcagcgtgc
agatggtagctgggatgtttattatggtgcacgtgccggtgatattaacaccaccgttgaagtttatgcagcactgcgttgtcagggttatgcagccgatcatccggatattaaacgtgcccgtgat
tggattcagctgcaaggtggtgtgaaacaggttcgtgtttttacccgtttttggctggcactgattggtgaatggccgtgggaagaaaccccgaatctgcctccggaaattctgtttttccgcgttgg
tttccgttcaacatttatcattttgcagcatgggcacgtgcgaccctggttccgctgtgtattctgagcgcacgtcgtatggttgttccgctgaacaaaaaaagctgtctgcaagaactgtttccgga
agatcgtagcgcagtggttgcactgggtaaaaaagccggtgcctggtcaacctttttctatcatgcagatcgtgccctgaaaaaataccagcgtacctttaaacgtccgcctggtcgtcagcag
gccattaaaatgtgtctggaatggattctgcgtcgtcaggatgccgatggtgcatggggtggtattcagcctccgtggatttatagcctgatggcactgaaagcagaaggttatccggttacacat
ccggttatggcaaaaggtctggcagcactggatgcccattggagctatgaacgtcctggtggtgcccgttttgtgcaggcatgtgaaagtccggtttgggataccctgctgagcagctttgcact
gctggattgtggttttagctgtaccagcagcagcgaactgcgtaaagcagttgactggattctggatcagcaggttctgctgcctggtgattggcagcaaaaactgccgaccgtttcacctggtg
gttgggcatttgaacgtgccaatgttcattatcctgatgttgatgataccgcagttgccctgattgttctggcaaaagttcgtcctgattatccagataccgcacgtgttaatctggccattgaacgtg
gtctgaattggctgtttgcaatgcagtgtcgtaatggtggctggggtgcatttgataaagataacgataaagacctgctgaccaaaattccgtttagcgattttggtgaaaccattgatccggcaa
gcgttgatgtgaccgctcatgtgctggaagcactgggcctgctgggttatcgtacaacccatccggcagttgcaaaagcactggaatttattcgtagcgaacaagaaaatgatggttgctggttt
ggtcgttggggtgtgaattatatctatggcaccgcagcagttttaccggcactggcaagcctgaatatgaacatgaatcaagaatttatccgtcgcgcagcaaattggattttaggcaaacaga
ataatgatggcggttggggtgaaagctgtgcaagctatatggatgatacccagcgtggtcgtggtccgagcaccgccagtcagaccgcatgggcaatgatgagcctgctggcagttgatggt
ggcacctatgccgaaagcctgctgcgtgcagaagcatatctgaaaaccacacagacaccggaaggcacctgggatgaaccgtattacaccggcaccggttttccaggttatggtattggtc
gtcgtgaaattaaacgtcagcgtagcctgcagcagcatgcagagctgagtcgtggttttatgattaattataacctgtaccgccactactttccgctgatggccctgggtcgcctggcagccctgc
gtggtgcataa

18. *Aca*ACH

atgctgccgtataatcaggatcatcattttggtaaagttgccgaaaatgcaaccatgcctccgacactggatgaagcaattgaacgtagccaggattttctgctgagcctgcagtatccggaag
gttattggtgggcagaactggaagcaaatgttaccctgaccgcacagaccattatgctgtacaaaattctgggcatcgatcataaatacccgatccacaaaatgaaaacctatattctgcgtac
ccagcgtgcacatggtggttgggaaatcttttatggtgatggtggctgtctgagcaccaccattggtgcatatatggcactgcgtattctgggtgttccgaaaaccgatccggtgctgcagaaag
cactgaaactgattcatagcaaaggtggtgttaccaaaagccgcatgtttacaaaaatttgtctggcactgctgggttgctatgattggaaaggtattccgagcctgcctccgtggctggttctgct
gccgagctggtttccgtttagcctgtatgataccgcaagctgggttcgtggttgtgttgttccgctgaccattatctttgataaaaaaccggtgtacaaactgaatccgctgctgtgtctggatgaact
gtatagcgaaggtaaaggtaaagcacgtgttcacctgagctttattccaggtgattggaccagcaactttttgttggtctggatcacgtgtttaaatacatggaaaatctgggcgttgttccgtttcg
tcagtggggtattaaagaagcagaacgttggaccctggaacgtcatgaagatagcggtgattttcatggcatttatccgcctatgttttatagcattgttagctatagcctgctgggctatgaaatta
cagatccggttgttcatcgtgcactggaaagcatgcgtggttttaccgttgaacgtgaagatgaatgcgttgttcagagctgtattagcccgatgtgggataccgcatttgttattcgtagcctggca
gaaagcggtctgcagccggatcatcctgcactgcaaaaagccggtgaatggctgctgcaaaaacaggcgacccagcatggtaattggttttataagaaacgtaccggtcgtgcaggcggt
tgggcatttcagtttttaaccgttggtatccggatgttgatgatagcgcagcagttagcatggcactgaatgcaattaaactgcaggacgatgatgttaaaaaaggtgccattaaacgttgtgcc
gaatggattagcgttatgcagtgtaaagatggtggatgggcagcctatgattgtgataatgatcgcgaatggctgaattgtaccccgtttggtgatctgaaagcaatgattgatccgaataccgtt
gatgttaccgcacgtgtgctggaaatggttggtcgtgtgaaagaggcaggcgacgcaagcgcaattctgcctccgcgtgcaattgcccgtggtctggcatatctgcgtcgtgaacaagaaac
cgaaggttgttggtatggtcgttggggtgtgaattatatctatggcaccagcggtgcactgatggcactggcactggttgcaccgagcacacataaagaagaaatcgaacgcggagcacgtt
ggctggttgaagttcagaataaacgtggcaccaaaggtgcaaatggttatagccataccaatggcgcacgtgaaggtggcgttgcaatgaatggcaattgtaaaaacatgggtgcaccgg
aagatggcggttggggtgaaacctgttttagctataatgatattaccctgaaaggtcgcaatgaagtttcaaccgttagccagaccgcatgggcactgcagggcctgctggcagccggtgatg
cactgggtaaatatgaagttgaaagcattgaacatggcgtgcagtatctgctgtcaacccagcgcaaagatggtagctggtgtgaaaaacattttaccggtggtggttttccgcgtttttctatatt
cgttatcatctgtatgccggtcattttccgctgagtgccctggcacgttatcgtgatcgtgttcgtgcaggtaaaatggccaaataa

19. *Bsu*TC

atgggcaccctgcaagaaaaagttcgtcgttttcagaaaaaaaccattaccgaactgcgtgatcgtcagaatgcagatggtagctggaccttttgttttgaaggtccgattatgaccaacagctt
ttttatcctgctgctgaccagcctggatgaaggtgaaaatgaaaaagaactgattagcagcctggcagcaggtattcatgcaaaacagcagccggatggcacctttattaactatccggatga
aacccgtggtaatctgaccgcaaccgttcagggttatgtgggtatgctggcaagcggttgttttcatcgtaccgaaccgcacatgaaaaaagcagaacagtttattatcagtcatggtggtctgc
gtcatgtgcattttatgacaaaatggatgctggcagcaaatggtctgtatccgtggcctgcactgtatctgccgctgagcctgatggcactgcctccgacactgccgattcatttttatcagtttagc
agctatgcccgtattcattttgcaccgatggcagttaccctgaatcagcgttttgttctgattaatcgcaatatcagcagcctgcatcatctggaccctcacatgaccaaaaatccgtttacctggct
gcgtagtgatgcatttgaagaacgtgatctgaccagcattctgctgcattggaaacgtgtttttcatgcaccgtttgcatttcagcagctgggcctgcagaccgcaaaaacctatatgctggatcgt
attgaaaaagatggcaccctgtatagctatgcaagcgcaaccatttatatggtttatagcctgctgagtctgggtgttagccgttatagcccgattattcgtcgtgcaattaccggtattaaaagcct
ggttaccaaatgcaatggtatcccgtatctggaaaatagcaccagcaccgtgtgggataccgcactgattagttatgcactgcagaaaaatggtgttaccgaaaccgatggtagcgttacca
aagcagccgattttctgctggaacgtcagcataccaaaattgcagattggagcgttaaaaatccgaatagcgttcctggtggttggggttttagcaatatcaataccaataatccggactgtgat
gataccaccgcagttctgaaagcaattccgcgtaatcatagtccggcagcatgggaacgtggtgttagctggctgctgagcatgcagaataatgatggtggttttagcgcctttgagaaaaatg
ttaatcatccgctgattcgtctgctgccgctggaaagcgcagaagatgcagcagttgatccgagcacagcagatctgaccggtcgtgttctgcattttctgggtgaaaaagttggctttaccgaa
aaacatcagcatattcagcgtgcagttaaatggctgtttgaacatcaagaacagaatggcagctggtatggtcgttggggtgtttgttatatctatggcacctgggcagcactgaccggtatgca

tgcctgtggtgttgatcgtaaacatccgggtattcagaaagcactgcgttggctgaaaagcattcagaacgatgatggttcatggggtgaaagctgtaaaagtgcagaaatcaaaacctatgtt
ccgctgcatcgtggcaccattgttcagaccgcatgggcattagatgcactgctgacctatgaaaacagcgaacatccgagcgttgttaaaggtatgcagtatctgacagatagcagcagcca
tagcgcagatagcctggcatatccggcaggtattggtctgccgaaacagttttatatccgctatcatagctatccgtatgtttttagtctgctggccgttggtaaatatctggatagcatcgaaaaag
aaaccgccaacgaaacctaa

20. *Gni*PNG

atggcactgccgtttaatcaggatagctataaaggtgatgatgaagccgatgttagcaaaggtgcagcaaaaagccctccgagcctggaagaagcaattcagcgtagccaagaatttctgc
tggcacagcagtttccggaaggtttttggtttggtgaactggaagcaaacgttaccattattagccataccgtgatcctgtataaactgctgggtatcgaagaaaacttcccgatgtataaattcga
acgttatctgcgtcgtatgcagtgtagtcatggtggttgggaaattgcctatggtattggtagctatctgagcgcaaccattgaagcatatattgcactgcgtctgctgaatgttccgcagagcgatc
cggcactgcagaaagcactgcgtgttattctggatagcggtggtgttaccaaagcacgtatttttaccaaaatttgtctggccctgctgggttcatttgattggcgtggtattccgagtctgcctccgt
ggctgattctgtgtccgacctggtttccgctgagcatttatgaagttagcagctgggcacgtggttgtattgttccgctgctggttatcctggataaaaaaccggtgtttaaagttagtccggaagtga
gctttgatgaactgtatgccgaaggtcgtgaacatgcctgtaaaatcattccgattagcggtgattggaccagcaaatttttcattaccgttgatcgcgtgttcaagatgatggaacgtctgcgtgtt
gttccgtttcgtcagtggggtattcgtgaagcagaaaaaatggattctggaacgtcaagaggaatcaggtgattacgttaacatttttccggcaatgttctatagcgtgatgtgcatgaaagttctgg
gttatgaaaccaccgatccggttgttcagcgtgcactgctgggctttaaaggttttaccattgaaaccgcagatgagtgtaaagttcagagcaccgttagtccgatttgggataccgcatttattgtt
cgtgcactggttgatagcggtattccgcctgatcatcctgcgctgcaaaaagcaggtcagtggctgctgcagaaacaaattctgaaacatggtgattgggcctttaaagatcgtcagaatccg
gtgaatcagcgtggttttgcatgtctgcagcgtgatagccagattgaaacagccgatgaatgtcgtgtgcagagcaccctgtcaccggtgtgggatacagcctttgttgttaaagccctggttgat
tcaggtattcctccgaaccatccggctttacagaaagctggccagtggttactgcaaaatcagaccctgacgcacggcgattgggcattcaaaacccagagcggtcatctggcagcaggcg
gttgggcgtttcagagccataatcgttggtatccggatgcagatgatagcgcagcagttatgatggcactggattgcattgaactgccggatgaagatgttaaaaatggtgcaattgcccgtggt
ctgaaatggattagcgcactgcagtcaagaaatggtggctgggcaggttatgataaaaactgtgatcagcagtggattaacaaagtgccgttcaatgatctgaatggcatcctggatgttccg
acagcagatgttaccgcacgtgttctggaaatggttggtcgtctgagccgtctgggtgcagttggcaccccgtatagtccgcgtcattgtaccctggtggaaagcattccgcatctgctgctgccg
gaaaccattgcacgcggtctggcatacctgcgtcgcgaacaagagggtgaaggttgttggtggggtaaatggggtgtgaattatatctatggcacctgtggtgcgctgctggccctgagccag
gttgcaccgaccacacatcaagaagaaatcgcacgcggagcaaaatggctggcccaggttcagaatcgttgtgataaacagaaagcagcacagggtccgcgtgatggtggatggggtg
aaagctgttttagctatgatgatcctgcactgaaaggtcagaatgatgcaagcaccgcaagtcagaccgcatgggcagtgcagggcctgctggcagccggtgatgcactgggtaaatatga
agttgaagcaattgaacagggtgtgcagtatctgctggcgacccagcgtaaagatggtacatggcatgaagcacattttaccggtagctgttttgcccagcatttttatgtgcgttatcactattatg
cgcagcattttccgctgtcagcactgggtctgtatcgtacccgtatcctgcagcatcagtaa

21. *Bte*SHC

atgaacagcgaactggaacgtctgaccgcagttctgcagcgtgaacagcaggcagatggtagctggcgttattgttttgaaagcggtccgctgaccgatgcctatgcaattattctgctgcgta
ccctgaatattccgaatgaaccgctgattagcggtctggcaaaacgtattgcaagccgtcaggcaccggatggcacctggaaactgtatagtgatgaacgtgatggtaatctgagcaccacc
attgaaagctattttgcactgctggcagccggtgcagcagcaccgtcagatgaacgtctgcaggcagcacgtcgttttattcgtgcaaaaggtggtctggcacaggcaaatctgggcacccgt
gttatgctggcactgacaggtcagcatccgtggccaccgtttccgattccggcagaatttatgctggttccgcctttttttccgctgcacctgtttgatctggttggttttgcacgtgttcatctggttccga
ttatggttgcagccgcacgtacctttgcagttcgtagtcgtcagatgccggatctgtcagacctgtttcgtggtctgccgagcgcaggtccgcagcagctggatctgatttggtttcatgaactgatt
gatagcggtattcgtagcctgccgagctttctgcgtccggcacgtgaactgggtctgcgtgaagcagaacgttttctgctggatcgtctggaaccggatggtacactgtatagctttttttaccgcaa
cctttctgatgattttcgccctgctggccctgggttatcgtccggatcatccggttattattcgcgcagttcgtggtgcagaacgtctggtttgtccggttggtgatgttctgcacatgcagaatagcac
cagcaccatttgggataccgcactgctgagccatgcactgcagaccgcaggtatgccggttagtcatccggtgattcagcaggcaacccgttatctgctgagtcgtcagcataatcgttatggt
gattgggcacgtcgtagtccgggtgttccgcctggtggttggggttttagcgatattaacaccattaatccggatgtggatgataccaccgcagcactgcgtgcactgcatcgtgcaggtagcgg
tgatccggcaattcgtcaggcatgggatcgcggtctgcgttggctgctgagtatgcagaattcagatggcggttggcctgcatttgaacgtaataccgcaaatccgctggttaaactgttaccgg
caggcggtgccgaagcagcatttaccgatccgagcaccgcagatctgaccggtcgtaccctggaatttctgggtaatcatgcaggtatgaccctgcagcatccggcagtgcgtcgtggtgttg
actggctgctgcgtcatcaagaaaccaatggtagttggcatggtcgttggggtatttcatatctgtatggtacatgggcagcactgagcggtctgattgcagcgggtgttagccctgatcatcctg
caattcagaaaggcgttagctggctgcgtagcgttcagaatcgtgatggtggctggggtgaaagctgtcagtcagatgttctgaaacgttatgttccgctgggtgcaagcaccccgagtcaga
ccgcatgggcagttgatgccctgacagcagttagccgtcgtaccggtccggaactggaagccggtgttcgttttctgttagcagcaggtaaacgtcgtgattggaccagcagctatccgaccg
gtgccgcactgccaggtggcttttatatccattatcatagctatcgctatatctggcctctgctgaccctggcacagtatcgtaacaaatttcagccgtaa

22. *Cth*SHC

atgcctggttttgcaccgcgttttgttcagccggttgttgaaagtccgctgcctccggcatttcgtagcgcacgtccggcaccggcaaccgcagcagcagttgaagcagcaattcgtaaagcac
aggcatatctgctgagcaaacagtatccggaaggttattggtgggcagaactggaagcaaatgttaccctgaccgcagaatatgtgtttctgcataaagttctgggcaccgatggtgaacgta
cccgtcagtttgaaaaaattcgtacctatctgcgtcgtcagcagcgtgaacatggtggttgggaactgtattatggtgatggtggtgaactgagcaccagcattgaagcctattttgcactgaaac
tgctgggtgatagtccggatctgccgcacatggcacgtgcgcgtcagtttattctggcacgtggtggtattaccaaagcacgtgtttttaccaaaattcacctggcactgtttggtgcatttccgtgg
gaaggttgtccgacactgcctccgtggattatgctgctgccggattggtttccgtttaccatttatgaactggcaagctgggcacgtagcagcaccgttccgctgctgctggttagcgatcgtaaa
ccggttgttcgtgttcctggtggtgatgcagatgaactgtatgccgaaggtcgtgcacaggccgatctgagcctgccgaatcctgcaggtctgctgagtttaggtggtgtttttattggttttgactgg
atgctgaaactgatggaacgttttgatctgagtccgcgtcgtgccgaagcactggcacgcgcagaacagtggaccctggaacatcaggatgatagcggtgattggggtggcattattccggc
aatgctgaatagcctgctgggtctgcattgtcgtggttatgcaccgacacatccggtaatgcagaaaggtattgcagccgttgaacgtttttgtatcgaaaccgaagatgaatttcatacccagc
cgtgtgttagtccggtttgggataccggtctgaccattctggccctgctggatagcggtctgccgaacgatcatccggcactggttcgtgccggtgaatggctgctgtcaaaacaaatttttcgtga
tggtgattggcgctttaaaaaccgtaccggtccggcaggcggttgggcatttgaattctggaatgatttttttccggatgtggatgataccgcagttgttacaatggcactgcatcgtttaaaactgc
ctgatgaagcagaaaaacagcgtcgtctgaaactggcaattgaatggaccctgagcatgcagagcaaaaatggtggctggggtgcatttgatgttgataataccctggaaatcctgaacga

tattccgtatggtgatctgaaagcaatgattgatccgcctaccgcagatctgacaggtcatattctggaaatgctgggcgttaccggttatgcagcaccgcgtgaaaaagttgaacgtgcaattg
cctttatcaagagcaaacaagaacctgaaggctgttggtggggtcgttggggtgtgaattacatttatggcacccacatggttatttgtggtctggttgcactgggtttagatccgcgtgaagccttt
attatgcgtggcacccagtggctgaatagttgtcagaatgaagatggcggatggggtgaaacctgtgcaagctatggcgatcgtaccctgatgggtgttggtaaaagcaccccgagccaga
ccgcatgggcactgctgggactgatggctggcggtgaaggtaaaagcgattgtgcccgtcgtggtattgaatatctggttacccatcagaacgatgatggtagctggaccgaagcagaattta
ccggtacaggctttccgaatcacttctatatgaactatcacttttaccgcaactactttccgctgatggcactgggtcgttatcgtgcatttgcacgtacctaa

23. *Ctt*SHC

atgaatctgagcggtcaggttgatcaggcagttggtcgtctgagcgaaagcctgagccgtatgcagagtgatgatggtagctggcgtttttgttatgaaaatgcagttctgaccgatgcctatatg
attattgcactgcgtaccctggaaattgcagatgaaccgctgattcgtcagctgcgtgatcgtctgctggcaacccagtatgcagatggtgcatggcgtgcatatccggatgaacgtgaaggta
atctgagtgcaaccgttgaatgttattatgcactgctgtatagcggttatagccgtgatgccgatccgcctctggttaaagcccgtgcatttattctggcaaaaggtggtattcagcagattggtggt
ctgctgaccaaagttatgctggcaagcaccggtcagtatccgtggcctcgtagcctgaaaatcccgctggaatttctgctgctgccgagccagtttccgctgagcgtgtttgattttagtggttatg
cacgtgttcacatgattccggcactgctgctggcagatcgtcgttttagcctgcgtaccaaaaccagtccggatctgagtgaactggcaggcgatcgtagccgtgaaccgcctagctggtttgat
ccggcattaggtcgtggtcatccgcgtgaactgcagagcctgctggaacaaattggtaaaggtattgaacgcctgaatggtattccgagccagctgcatgaagaagcagttcgtcgcgcag
aacgttatatgctggaacgtattgaagcagatggcaccctgtatagctatgcaaccagcacctttctgatgatcctggcgctgctggccattggttatgataaacgtcatgcagttattcgtaatgc
cgttcagggtctgcgtgcaatggtttgtcgtggtgcacagccgatttttctgcaaaatgcaccgagcaccgtttgggataccgcactgctgagcagcgcactgcaagaggcaggcgcagatg
caaatagcccgatgattcgtcgtgcaaatgcatatctgctggcgaaacagcatcgtaaacctggtgattggctggttcataatccgagcgcagttccaggtggttggggttttagcgataccaat
accattaatccggatgttgatgataccaccgcagcactgcgtgccattaaacgtcaagccggtgcagatcctgcatatcgtgaagcatggaatcgtggtctgcattggctgctgtctatgcaga
acgatgacggtggttggcctgcatttgaaaaaaacaccgatcgtcagattctggttctgctgccgctgcgtgaagcaaaaagcagcgcaattgatccgagcaccagcgatctgaccggtcg
caccctggaatttttaggtaattatgcaggtttaggtatgggccatgcctttattcgccgtggcaccgattggctgattggtcatcaagaaaaagatggttcatggtatggtcgttggggtgtttgttat
atctatggcacctgggcagcactgacaggtctggcagcagccggtattcgtgcagatcatccggcagttcgtgccggtgcacagtggctgaccgatattcaacaggccgatggtggctggg
gtgaaagctgtgatagcgatcgccagatgcgttatattccgctggaagaaagcaccccgagccagaccgcatgggcattagatgcactgattgcagttcatgaagcaccgacaccgacca
ttgatcgtggtattcgtcgtctgatcggcctgctgcaagaagaaagccgttttgcagcatatccgaccggtgcaggtctgcctggtatcttttatagccactattatagctatcgctacatctggcctc
tgtttgcactggcacactataaaagcaaatatagcagctaa

24. *Gth*SHC

atggcaggcgaacgtagcgcactgattaccgcactgaaacgtagccaggcagcagatggtagctggcgttttccgtttgaaaccggtattagcaccgatgcctatatgattattctgctgcgta
ccctggatattaatgatgaaccgctgattcaggcactggttgaacgtattgaaagccgtcaagaggcaaatggtgcatggaaactgtttgcagatgaaggtgatggtaatgttaccgcaaccg
ttgaagcatactatgcactgctgtatagcggttatcgtcagccgaccgatcgtcacatgcagaaagcaaaacgtcgtattctggatatgggtggtctggatcgtattcacctgtttaccaaagttat
gctggcactgacaggtcagtatccgtggccaggtcgttttccgctgccgctggaatttttctgctgccaccgagctttccgctgaatatgtatgatctgagcgtttatggtcgtgccaatatgattcc
gctgctgattgcagcagatagccgttatagccgtaaaaccgataaaagtccggatctgagtgacctgtttgcaagccgtggtgattggggtatgccggaaagccgtagcctgctgacctatgtt
aaacgtagtctgattggtctgcctgcacagctgcatcaggcagcaaaacagcgtgcagttcgttacctgtttgaacatatcgaaccggatggcaccctgtatagctattttagcagcacctttctg
tttatctttgccctgctggccctgggttatcgtaacgatgatccgcgtattcgtcaggcagttcgtggtctgcgttcactgcgtaccaccattgatggtcatgtgcatctgcagtataccaccgcaagc
gtttggaatacagcactggcaagctataccctgcaagaggcaggcgttccgatgaccgatcgcgcaattgaaaaagcaaatcgttatctgctgagccgtcagcatgttcgttatggcgattgg
gcagttcataatccgtatagcacccctggtggttggggttttagtgatgttaataccatgaatccggatgtggatgataccacagcagcactgcgtgcaattcgtcaagcagcagcaaaagaa
accgcatttcgtcatgcatgggatcgtgcaaatcagtggctgtttagcatgcagaatgatgatggtggttttgcagcctttgaaaaaaatgttagcagccgtttttggcgctatctgccgattgaag
gtgcagaatttctgctgatggacccgagcaccgcagatctgaccggtcgcaccctggaatatttcggcacctttgcaggtctgaccaaagatcagcgtgccgttagccgtgcagttgattggct
gctgagtcatcaagaacgtaatggtagttggtatggtcgttggggtatttgttatatctatggcacctgggcagcaattaccggtctgaccgcagttggtgttccggcacatcatccggcactgca
aaaagccgttcgctggctgctgtcaattcagaacgatgacggtggctggggtgaaagctgtaaaagtgatggtgcaaaaacctatgttccgctgggtgatagcacaccggttcataccgcat
gggcattagatgccctggttgcagcagcagaacgtccgacaccggaaatgaaagcaggttttcgtgcactgtttcgtctgctgcatcatcctgattggaccgcaagctatccggttggtcaagg
tatggctggtgccttttatatccattatcatagctatcgctatatcttcccgctgctggcgctggcacattatgaacagaaatttggtccgctggatgactaa

25. *Sth*SHC

atggaccctgcactgagccgtgcagttgattggctgctggaacatcaagatccggcaggttggtggtgtggtgaatttgaaaccaatgttaccattaccgcagaacatattctgctgctgcgtttt
ctgggtttagatccgagtccgctgcgtgatgcagttacccgttatctgctgggtcagcagcgtgaagatggtagctgggcactgtattatgaaggtccggcagatctgagcaccagcattgaag
cctatgcagcactgaaagttctgggccttgatccgaccagcgaaccgatgcgtcgtgcactgcaggttattcatgatttaggtggtgttgcacaggcacgtgtttttacccgtatttggctggcaat
gtttggtcagtatccgtgggatggtgttccgagcatgcctccggaactgatttggctgcctccgagcgcaccgtttaatctgtatgattttgcatgttgggcacgtgcaaccattacaccgctgctga
ttattctggcacgtcgtccggttcgtccgctgggttgtgatctgggtgaactggttctgcctggtagcgaacatctgctgacacgtgttccaggtagcggtccgttttggtggggtgataaagttctga
aacgttatgatcatctggttcgtcatccgggtcgtgatcgtgcatgtcagcgtattgttgaatggattattgcacgtcaagaagcagacggtagttggggtggtattcagagcgcatgggttatgag
cctgattgcactgcatctggaaggtctgccgctggatcatccggttatgcgtgcaggtctggcaggttttgatcgtgttgcactggaagatgaacgtggttggcgtctgcaggcaagcaccagtc
cggtttgggataccgcatgggcagttctggcactgcgtcgcgcaggtctgcctcgtgaacatccgcgtctggccctggccgtggactggctgctgcaagagcagattcctggtggtggtgattg
gcaggttcgtaccggcaccattccaggcggtggttgggcatttgaatttgataacgatcattatccggacatcgatgataccgcagttgttgttctggctctgctggaagcaggtcatgaagatcg
tgttcgtaatgcagttgaacgtgcagcccgttggattctggccatgcgtagcaccgatggtggctggggtgcatttgatcgcgataatgcccgtgaagttattcatcgtctgccgattgcagattttg
gcaccctgattgatccgcctagtgaagatgttaccgcacatgttctggaaatgctggcacgcctgagctttccgagcaccgatccggttgttgcccgtggtctggaatttctgcagcagacccag
cgtccggatggtgcatggtttggtcgttggggtgtgaattatatctatggcacctggtgtgcagtttcagcactgaccgcatttgcagataccgatgcaaccgcacgcgcaatggttccgcgtgcc
gttgcctggctgttagatcgtcagaatgcagatggcggttggggtgaaacctgtggtagctatgaagatccgaatctggcaggcgttggtcgtagcaccccgagccagacagcctgggcagt

tttagccctgcaggcagccggtctgggccagcatcctgcatgtcgtcgcggtctggatttcctgcgtgaacgtcaggttggcggtacatgggaagaacgtgaacacaccggcaccggttttcc
gggtgatttttcattaactatcatctgtatcgccacgtgtttccgacaatggcattagccggtgcagcaaccggtatggatagtccgcgttaa

26. ***Tsg*SHC**

atgcagagccagtggattctgcatgttcaggcaatgattcatcgtctgaaaaaagagctgatccagaaacagaatccggatggcacctggccgttttgttttgaaaatggtattggcaccgatg
cctattatgttctgctgcatcaggttctgaaacgtcctgatccgggtacactggcaccggttctggaacgtattctggataaacagaccggtgatggtacatggaaagcatttccggatgaaaaa
gaaggtaacgttagcgcaaccctggatgcaagcctggcactgctgtatagcggtgttaaaacaccggatgatccgagcctgaaacgtgcccgtgattttctgctgagccgtggtatggaaac
caaagcaggtagtctgacccaggttgttctggccctgctgggtcatcgtagctggtcacgtattaccaaactgccggttgaatttttctgcttccggcagcaagtccggttaactttttgattttgttg
gttatgcccgtgtgcatattgcaccggttatgctggcaagcgatcaggatttctatattcatctgaaaggttatcgcgaggttgaagattggctgccgagcagctttcgtacctatatggaacgcat
gcatccggactatttttggaccaaagaagatctgctgcctgcagttgaaaccaccgcatatagcacctttttaagaaaaatgttcatcagcgtgccctgtattggggtgaaaactttctgctgtctc
gtattgaagaagatggcaccctgtatagttatatgaccagcacctttctgatgattttgcactgctgagtctggattatccgcctgatcatccgctgattcagaaagcaatggaaggtctggatcgt
atgatttttccgctgcaagaaggtgcacatctgcaagaggcaaccagcaccgtttgggataccagcctggttatgaccgcactgcagaatgcaggtctgagccctggtcatggtgttattcaaa
aaggtcgtaattacctgctgctgaaacagcataccagctgtggtgattggtgcctgaaaaatcgttatgcaattcctggtggttggggttttagcgataccaatacaattaacccggatgttgatg
ataccgttcagtgtctgcatgcaattgctccggcagttcgtgaaggttgggcacaagatgaatggaaacgtggtctgcagtggctgctgagtatgcagaaccgtgatggtggctggcctgcattt
gaacgtaataccaataaaatgtggctgaaactgctgccagcacgtaatgaaaaacgtgtttggggtgatccgagtaccgcagatctgaccggtcgtgttctgcattttctgggtagcgaattag
gttggaccattgatcgtccggaagttcgtcgtgcatggtcatggctgtatcatcaccagaattcagatggtagctggtttggtcgttggggtgtgagctatatctatggtacgtgggcagcactgaa
aggtctggcagccgttggtgttccggaaacacatgttagcgtgcagaaaggtattcgtttcctgctgagcaaacagcgtccggatggtggatggggtgaatcatgttatagtgatgcagaagat
cgttttgttccgctgagctttagcacaccggttcagaccgcatgggcattagatgcactgattgcatatcatgatcatcctacaccggcaattgaaaaaggtatggcctgtctgctggaaatgatg
gaaaaacgcggtgaagaatggtcatatccggcaggcgcaggtctgtcaggtcagttttatgtttattatcacagctacccgtatgtttggagcctgatggcaatgacccattatctgcagaaatat
agctaa

27. ***Afa*SHC**

atgaacaccattagccatccgagcaaagttaaagcagcagttagcgcaaccgttccgcctacaccgagctgtgttaccccgacaccgtttaccggtatgggtaatagcctggcacataccgt
tgcagcagcatgtgattggctgattggtgaacagaaagcagatggtcattgggttggtccggttgcaagcaatgcaagcatggaagcagaatggtgtctggcactgtggtatctgggtttaga
agatcatccgctgcgtccgcgtctgggtaaagcactgctgcacatgcagcgtgaagatggtagctggggcacctattggggtgcaggtaatggtgatattaatgcaaccgttgaagcctatgc
agcactgcgtagcctgggttatgcagcagatacaccggaactgagcaaagcatgtgcatggattatgcgtatgggtggtctgcgtaatgttcgtgtttttacccgttattggctggcactgattggc
gaatggccgtgggaacagaccccgaatctgcctccggaagttatttggtttccgaacaaattcgtgttcagcatctataactttgcacagtgggcacgtgcaaccctggttccgctggcaattct
gagcgcacgtcgtccgagccgtccgttacgtccgcaggatcgtctggatgcactgtttccgcagggtcgtgaaaattttgattatgtgctgccgaaaaaagaaggcgttgatctttggagcagct
ttttcgtaccaccgataaaggtctgcattggctgcagagccgttttctgaaacgtaataccgttcgtgaagcagcaattcgccacatgctggaatggattattcgtcatcaggatgcagatggtg
gttggggtggtattcagcctccgtgggtttatggtctgatggcactgcatggtgaagattatcagtttcatcatccggttatggcaaaagcactggcagcactggatgatcctggttggcgtcgtgat
cagggtgatgcaagctgggttcaagcaaccaatagtccggtttgggataccatgctggccctgatggcattacatgatgcaaatgcagaagaacgttatacaccgcagatggataaagccc
tggactggctgctggcacgtcaggttcgtgttaaaggtgattggagcattaaactgccggatgttgaacctggtggctgggcatttgaatatgccaatgatcgttatcctgataccgatgataccg
cagttgccctgattgcactgagcagctgtcgtaatcgtgaagaatggaaagaaaaaggtgtggaagatgcaattacccgtggtgttaattggttaattgcaatgcagagcagttgtggcggttg
gggagcatttgataaagataataatcgtagcctgctgagcaaaatcccgttttgtgattttggtgaagcactggaccctccgagcgttgatgttaccgcacatgttctggaagcatttggtctgctg
ggtgttccgcgtcagacaccggcactgcaacgtggtctggcatatattcgtgcagaacaagaggcaagcggtgcatggtttggccgttggggtgtgaattatctgtatggtacaggtgcagttct
gcctgcgctggcagcaattggcgaagatatgacccagccgtatattacacgtgcctgcgattggcttattgcacatcagcaagaggatggcggatggggtgaaagctgtgcaagctatatgg
atgttagcagcattggttggggcaccaccacaccgagccagaccgcatgggcactgatgggtctgattgcagcaaatcgtgaacaggaccatccggcaattgcacgtggttgtcgttatctg
attgatcgtcaagaaaccgatggtagttggaccgaagaagaattcaccggcaccggttttccaggttatggtgttggtcagacaattaaactggacgatccagcagttgcaaaacgtctgcag
cagggtgcagaactgagccgtgcatttatgctgcgttatgatctgtatcgtcagtttttccgctgatggccctgagtcgtgcagcacgtattatgccggttggtcagtaa

28. ***Aor*SHC**

atgaccacaccgctgtttaaaggtatgggtaatagtctgacccataccgttagcagcgcatgtgaatggctgattagccagcagaatccggatggtcattgggttggtccggttggtagcaatg
caagcatggaagcagaatggtgtctggcactgtggtttctgggtttagaagatcatccgctgcgtccgcgtctgggtaatgcactgctgcagacccagcgtgaagatggtagctgggatgtttat
ttaggtgcaggtaatggtgatattaatgcaaccgttgaagcctatgcagcactgcgtagcctgggttatccggaaaatacaccggcactgcagaaagcagcaacctggattaaacagaaag
gtggcctgaaaaacattcgtgtgtttacccgttattggctggcactgattggtgaatggccgtgggaaaaaaccccgaatctgcctccggaaattatctggtttccgaacaaattcgtgttcagca
tctataactttgcacagtgggcacgtgcaaccctggttccgctggcaattctgagcgcacgtcgtccgagccgtccgttacgtccgcaggatcgtctgaatgcactgtttccggaaggtcgtggt
aattttgattataccctgccgaaaaaagaaggcgttgatctttggagcgatttttttcgtaccaccgataaaggtctgcattggctgcagagcaaatttctgaaacgtaataccatgcgtgaagca
gcaattcgtcacatgctggaatggattattcgtcatcaggatgcagatggtggttggggtggtattcagcctccgtgggtttatggtctgatggccctgcatggtgaaggttatcagtttcatcatccg
gttatggcaaaaggtctggatgccctgaatgatcctggttggcgtcatgataaaggtaatgccagctggattcaggcaaccaatagtccggtttgggataccatgctggccattatggcactgc
atgatgcaaaagcagaagatcgttttacaccgcaggttgataaagcattaggttggctgctggatcgtcaggttcgtgttaaaggtgattggagcattaaactgccggatgttgaacctggtggc
tgggcatttgaatatgccaatgatttttatccggacaccgatgataccgcagttgccctgattgcactggcaagctgtcgtcatcgtccggaatggcaagaacgtggtgttgaagatgcaattgc
ccgtgcagttcgttggctggttgccatgcagagcagctgtggcggttggggagcatttgataaagataataacaaagccctgctgagcaaaatcccgttttgtgattttggtgaagcactggacc
ctccgagcgttgatgttaccgcacatattctggaagcatttggtctgctgggcctgcctcgtgatctgccgtgtattaaacatgcactggattatgttcgtgcagaacaggacccgcaaggtccgt
ggtttggccgttggggtgttaattatgtttatggtacaggtgcagttctgccagcactggcagcaattggtgaagatatgacccagccgtatattaccaaagcctgtgattggctggtagcacatca

gcaagaggatggcggatggggtgaaagctgtgcaagctatatggatgcaagcaccattggtcgcggtaaaaccacaccgagccagaccgcatgggcactgatgggtctgattgcagca
gcacgtcctcaggattatccggcaattgaaaaaggttgtcgctatctgattgatcgtcaagaaccggatggcagctggaccgaagaagattacaccggtacaggttttccaggttatggtgttg
gtcagaccattcgtctggatgatccggcactgagcaaacgcctgcagcagggtgcagaactgagccgtgcatttatgctgcgttatgatctgtatcgtcagtttttccgattatggccctgagtcg
tgcaagccgtctgattagtccggaaaccgcaaccgaacaggcagttgaagcagccgcaaaaaatctggaaaagattattgcctaa

29. *Gfr*SHC

atgggtgtttggcgtatgagcgcaccgattctgaaaggtatgagcaatagcctggcacataccgttagctgtgcatgtgattggctgattggtcagcagaaagcagatggtcattgggttggtag
cgttgaaagcaatgcaagcatggaagcagaatggtgtctggcactgtggtttctgggtttagaagatcatccgctgcgtccgcgtctgggtaatgcactgctggaaatgcagcgtgatgatggt
gcatggggtgtttatctgggtgcacagagcggtgatattaatgcaaccgttgaagcctatgcagcactgcgtagcctgggttatagcgcaaatagtccggttctgctgaaagccggtgcatgga
ttagcgaaaaaggtggtctgaaaaacattcgtgtgtttacccgttattggctggcactgattggtgaatggccgtgggaaaaaaccccgaatctgcctccggaaattatctggtttccgaacaatt
ttgtgttcagcatctataactttgcacagtgggcacgtgcaaccctggcaccgctggcaattctgagcgcacgtcgtccgagccgtccgttacgtccgcaggatcgtctggatgcactgtttccg
gaaggtcgtgaaaaatttgattataccctgccgaaaaaagatcgcgttgatctgtggtctagctttttcgtaccaccgataaaggtctgcattggctgcagagccgttttctgaaacgtaataccg
ttcgtgaagcagcaattcgtcacatgctggaatggattattcgtcatcaggatgcagatggtggttggggtggcattcagcctccgtgggtttatggtctgatggccctgcatggtgaaggttatcc
gtttcatcatccggttatggcaaaagcactggcagcactggatgatcctggttggcgttatgatcgtggtgaagcaagctggattcaggcaaccaattcaccggtttgggataccatgctggccc
tgatggcattacatgatgcaaatgcacaagaacgttttacaccggaaatggataaagcattaggttggctgctggaacgtcaggttcgtgttaaaggtgattggagcattaaactgccggatgtt
gaaccaggtggttggagctttgaatatgcaaatgatcgttatccggacaccgatgataccgcagttgccctgattgcactgagcttttgtcgtcatcgtgaagaatggaaacagaaaggcgttg
ataaagcaattgatcgtgcagtgaactggctgatcgcaatgcagagcagctgtggtggctggggagcatttgataaagataataacaaaagcctgctgagcaaaatcccgttttgtgattttgg
tgaagcgctggaccctccgagcgttgatgttaccgcacatattctggaagcatttggtctgctgggtctgagccgtgatctgccggttgttcagaaagccctggcctatgttcgtctggaacagga
cccgcaaggtccgtggtttggccgttggggtgttaattatctgtatggtacaggtgcagttctgcctgcgctggcagcaattggtgaagatatgacccagccgtatatcctgaaagcgtgcgaat
ggctgattagctgtcagcaggatgatggcggatggggagaaagctgtgcaagctatatggatattagcagcattggtcgtggtagcaccaccgcaagccagaccgcatgggcactgatgg
gtctgattgcagttggtcgtcctcaggatcatgaagcaattgcaaaaggttgtcgctttctgattgatcgtcaagaggcagatggtagctggaccgaagaagaattcacaggcaccggttttcca
ggttatggtgttggtcagacaattaaactggacgatccggcactgagcaaacgtctgatgcagggtgcagaactgagtcgtgcatttatgctgcgctatgatatgtatcgtcagtattttccgatta
tggcactggcacgtgcgagccgtctgctgacacaggatatttaa

30. *Kna*SHC

atgaatagcgaaagccgtctgagccgtaaacaggcaggcgcaccgggtcctgataaaattgaagcacgtccggatagcacaccggcagcagcatttcgtggtattgataatagcctgaca
cataccctgagcagcgcatgtaaatggctgatggaacagcagaaaccggatggtcattgggttggtagcgttgcaagcaatgcaagcatggaagcagaatggtgtctggcactgtggtttct
gggtttagaagatcatccgctgcgtccgcgtctgggtaaagcactgctggaaatgcagcgtgaagatggtagctggggtatctattatggtgcaggtaatggtgatattaatgccaccgttgaa
agctatgcagcactgcgtagcctgggttatgcagcagatgatccggcactgagccgtgcagcaacctggattgccagcaaaggtggtctgcgtaatgttcgtgtttttacccgttattggctggc
actgattggtgaatggccgtgggaaaaaaccccgaatctgcctccggaaattatctggtttccgaacaaattcgtgttcagcatctataactttgcacagtgggcacgtgcaaccctggttccgc
tggcaattctgagcgcacgtcgtagcagccgtccgttacgtccgcaggatcgtctggatgccctgtttcctggtggtcgtgaaaattttgattatgaactgcctccgcgtgatggtcaggatctgtg
ggcaacctttttcgtaaaaccgatcgtgcactgcattggctgcagaccaaatttctgaaacgtaataccatgcgtgaagcagcaattcgtcacatgctggaatggattattcgtcatcaggatgc
agatggtggttggggtggtattcagcctccgtgggtttatggtctgatggccctgcatggtgaagattatcagtttcatcatccggttatggcaaaaggtctggcagcactggatgatcctggttgg
cgttatgatcgtggtgatgcaagctgggttcaagcaaccaatagtccggtttgggataccatgctggccctgatggcattacatgatgccgatgccgaaaccgattttacaccggaaatggata
aagcattaggttggctgctggaacgtcaggttcgtgttaaaggtgattggagcgttaaactgccggatctggaacctggtggctgggcatttgaatatgccaatgatcgttatcctgataccgatg
ataccgcagttgccctgattgcactggcagcatgtcgtgatcgtgaagaatggaaaggtcgtggtgttgaagccgcaattacccgtggtgttaattggctggttggtatgcagagcacctgtggc
ggttggggagcatttgataaagataataatcgtgccctgctgagcaaaattccgttttgtgattttggtgaagcactggaccctccgagcgttgatgttaccgcacatgttctggaagcatttggtgt
tctgggtctgcctcgtgatatgcctgcactgcagcgtggtctggcatatattcgtgcagaacaagaggcagatggtccgtggtttggtcgctggggtgtgaattatctgtatggtacaggtgcagtt
ctgcctgcgctggcagcaattggcgaagatatgacccagccgtatattgcccgtgcatgtgattggttagttgcacatcagcaagaaaatggcggatggggtgaaagctgtgcaagctatatg
gaaattgcaagcattggtcgtggtccgaccacaccgagccagaccgcatgggcactgatgggtctgattgcagcaaatcgcaaacaggatcatgaagcaattgttcgtggttgccgttatctg
attgatcagcagcaggccgatggtagttgggaagaaaaagaattcaccggcaccggttttccaggttatggtgttggtcagacaattaaactggacgatccagcactgaccagccgtctgca
gcagggtgcagaactgagtcgtgcatttatgctgcgctatgatctgtatcgtcagtttttccgattatggcactgtcacgtgcagttcgtgttctgaaaggtagcaaataa

31. *Kxy*SHC1

atggataccaccggtcatacaccggttaccaccgcaccggcagcaccggatgcaaccggtacacagacccagaccgcaggcgcaccgtttgcaggtatgggtaattctatgacccatac
cattagcgcagcatgtgattggctgattcagcagcagaaaccggatggtcattgggttggtagcgtgggtagcaatgcaagcatggaagcagaatggtgtattgcactgtggtttctgggtttag
aaaatcatccgctgcgtccgcgtctgggtaatgcactgctggaaatgcagcgtgaagatggtagctggggtgtttatcatggtgcaggtaatggtgatattaatgcaaccgttgaagcctatgc
agcactgcgtagcctgggttatccggcagatacaccggcactggcacgtgcagcaacctggattgcacgtaaaggtggtctgcgtaatattcgtgtttttacccgttattggctggcactgattgg
tgaatggccgtgggaaaaaaccccgaatctgcctccggaaattatctggtttccgaataaattcgtgttcagcatctataactttgcacagtgggcacgtgcgaccctggttccgctggcaattct
gagcgcacgtcgtccgagccgtccgttacgtccgcaggatcgtctggatgcactgtttccggaaggtcgtgcaaattttgattataccctgccgaaaaaagaaggtcgcgatctttgggcaac
ctttttcgtaccaccgatcgtggtctgcattggctgcagagcaatgttctgcgtcgtagcaccatgcgtggtgcagcaattcgccacatgctggaatggattattcgtcatcaggatgcagatggt
ggttggggtggtattcagcctccgtgggtttatggtctgatggccctgcatggtgaagattatcagctgcatcatccggttatggcacgttcactgggtgcactggatgatcctggttggcgtcatga
tcgtggtaatgccagctggattcaggcaaccaatagtccggtttgggataccatgctggccctgatggcattacatgatgcaggcggtgaagatcgttttacaccggaaatggatcgtgccctg
gactggctgctggcacgtcaggttcgtgttcgtggtgattggagcattaaactgccggatgttgaacctggtggctgggcatttgaatatgccaatgatcgttatcctgataccgatgataccgca
gttgccctgattgccctggcaccgtgtcgtaatcgtccggaatggaaagaaaaaggtgttgatgcagccattgatcgtgcagttcgttggctggttgcaatgcagagcgaatgcggtggatggg

gtgcatttgataaagataataatcgtagcctgctgagcaaaatcccgttttgtgattttggtgaagcactggaccctccgagcgttgatgttaccgcacatattctggaagcatttggtctgctgggt
ctgcctcgtgatatgcctgcaattcagcgtgcactggcctatgttcgtgcagaacaagatccggcaggtccgtggtttggccgttggggtgttaattatgtttatggtacaggtgcagttctgcctgc
actggcagcaattggcgaagatatgacccagccgtatattgcccgtgcctgcgattggctggtagcacatcagcaagaggatggcggatggggagaaagctgtgcaagctatatggaaatt
gcaagcgttggtcgtggcaccaccacaccgagccagaccgcctgggcagttatgggtttagttgcagcaaatcgtgcacaggattatccagcaattgcgcgtggttgtcgttatctgattgaac
gtcagcagccggatggtagttggcatgaagccgaattcaccggcaccggttttccaggttatggtgttggtcagacaattaaactggatgacccgatgctgagccagcgtctgagccagggt
gcagaactgagccgtgcatttatgctgcgttatgatctgtatcgtcagctgtttccgattatggccctgagtcgtgcagcacgtctgatgccggttggtggcgcaaaacagcagggtacagtttaa

32. *Kxy*SHC2

atgacccgtgaaagccgtccgctgaccaaaaccgcagcaagcagcggcaatattacaagcggtaatacctttccggcagttggcacccagagcgttaatggtggtggtaaaagcaccgg
tagcgcaagcgcactgcgtacaatggataatagcctgagccatgcaattagcagcgcatgtgattggctggttggtcagcagaaaccggatggtcattgggttggtccggttgcaagcaatg
caagcatggaagcagaatggtgtctggcactgtggtttctgggtttagatgatcacccgctgcgtccgcgtctgggtaaagcactgctggaaatgcagcgtgaagatggtagctggggcacct
attatggtgcaggtaatggtgatattaatgccaccgttgaaagctatgcagccctgcgtagcctgggttatccggcagatgatccggcaattagccgtgcagcaacctggattgccagcaaag
gtggtctgaaaaacattcgtgtttttacccgttattggctggcactgattggtgaatggccgtgggaaaaaaccccgaatctgcctccggaagttatttggtttccgaacaattttgtgttcagcatct
ataactttgcacagtgggcacgtgcaaccctggttccgctggcaattctgagcgcacgtcgtccgagtcgtccgttacgtccgcaggatcgtctggatgccctgtttcctggtggtcgtgcaaattt
tgattatgaactgcctgcacgtggtgatcgtgatctgtgggatcgtttttttcgtgcaaccgatcgtggtctgcattggctgcagagccgttttctgaaacgtaataccctgcgtgaagcagcaattc
gtcacatgctggaatggattattcgtcatcaggatgcagatggtggttggggtggtattcagcctccgtgggtttatggtctgatggccctgcatggtgaagattatcagtttcatcatccggttatgg
caaaagcactgagtgcactgaatgatcctggttggcgtcatgataaaggtgatgcaagctggattcaggcaaccaatagtccggtttgggataccatgctggccattatggcactgcatgatg
ccgatggtgaaacccagtttagtccgcagatggaaaaagcattaggttggctgctggatcgtcaggttcgtgtgaaaggtgattggagcattaaactgcctgatgttgaacctggtggctgggc
atttgaatatgccaatgatcgttatcctgataccgatgataccgcagttgccctgattgcactgagcagctgtcgtaatcgtgaagaatggaaaaaacgtggtgttgaagaggcaatttctcgtg
gtgttaattggctgattggcatgcagtcagaatgtggcggttggggagcatttgataaagataataatcgtagcatcctgagcaaaatcccgttttgtgattttggtgaagcactggaccctccga
gcgttgatgttaccgcacatgttctggaagcatttggtattctgggtctgcctcgccacatgccgaccattcagcgtgcactggcatatattcgtgcagaacaagaacctgatggtccgtggtttgg
tcgctggggtgtgaattatctgtatggtacaggtgcagttctgccagcactggcagcaattggcgaagatatgacccagccgtatattaccaaagcctgcgattggcttgttgcacatcagcaa
gaaaatggcggatggggtgaaagctgtgcaagctatatggaactgagcatggttggtcgtggtgtgaccacaccgagccagaccgcatgggcactgatgggtctgattgcagcaaatcgtc
ctcaggattatggcgcaattgcccgtggttgtcgttatctgattgatctgcagcaggcagatggttcatggcatgaaaaagaattcaccggcaccggttttccaggttatggtgttggtcagacaat
taaactggatgatccagcgctgagcaaacgtctgcagcaaggtgcagaactgagccgtgcatttatgctgcgttatgatctgtatcgtcagtttttccgattatggccctgagtcgtgcaagccgt
ctgatgaaattagaaaaataa

33. *Aci*SHC_R1.1

atgacccaggcaagcgttcgtgaagatgcaaaagcagcactggatcgtgcagttgattatctgctgagcctgcaggatgaaaaaggtttttggaaaggtgaactggaaaccaacgttacca
ttgaagccgaagatctgctgctgcgtgaatttctgggtattcgtacaccggatattaccgcagaaaccgcacgttggattcgtgcaaaacagcgtagtgatggcacctgggcaaccttttatgat
ggtccgcctgatctgagcaccagcgttgaagcctatgttgcactgaaactggctggtgatgatccggcagcaccgcacatggaaaaagcagccgcatatattcgtggtgccggtggtgttga
acgtacccgtgtttttacccgtctgtggctggcactgtttggtctgtggccgtgggatgatctgccgacactgcctccggaaatgatttttctgccgagctggtttccgctgaacatttatgattggggtt
gttgggcacgtcagaccgttgtgccgctgaccattgttagcgcactgcgtccggttcgtccgattccgctgagtattgatgaaattcgtacaggtgcaccgcctccgcctcgcgatccggcatgg
accattcgtggtttttttcagcgtctggatgatttactgcgtggttatcgtcgtgttgcagatcatggtccggcacgtctgtttcgtcgtctggcaatgcgtcgtgcagcagaatggattattgcacgtca
agaggcagatggtagctggggtggtattcagcctccgtgggtttatagcctgattgcactgcatctgctgggttatccgctggatcatccggttctgcgtcgtggtctggatggtctgaatggttttac
cattcgcgaagaaacagcagatggtgcagttcgtcgcctggaattttgccagagtccggtttgggataccgcactggcagttacagcactgcgtgatgcaggtctgcctgccgatcatccgcg
tgttcaggcagcagcccgttggctggttggtgaagaggtgcgtgttgccggtgattgggcagtgcgtcgtccgggtctgcctcctggtggttgggcatttgaatttgccaatgataattacccggat
accgatgatacagcagaagttgttctggccctgcgtcgcgttcgtctggaagatgcagatcagcaggcgctggaagcagccgttcgtcgtgcaaccacctgggttattggtatgcagagcac
cgatggcggttggggtgcatttgatgcagataatacccgtgaactggtgctgcgtctgccgttttgtgattttggtgccgttattgatccgcctagcgcagatgttaccgcacatattgttgaaatgct
ggcagccctgggtatgcgtgatcatcctgccaccgttgcgggtgttcgttggctgctggcacatcaagaacctgatggtagttggtttggtcgttggggagcaaatcatatttatggtacgggtgc
agttgttccggcactgattgcagccggtgttagtccggatacaccgcctattcgtcgcgcaattcgctggctggaagaacatcagaatccggatggtggatggggtgaagatttacgtagctat
accgatcctgcactgtgggttggtcgtggtgttagcaccgcaagccagaccgcatgggcactgctggcattactggcagcgggtgaagaagcaagtccggcagttgatcgtggcgttcggtg
gctggttaccacacagcagcctgatggtggctgggatgagccgcattacaccggtacaggttttccgggtgatttctatattaactatcatctgtatcgcctggtgtttccgattagtgcactgggtc
gttatgttaatcgt

34. *Aci*SHC_R2.1

atgacccaggcaagcgttcgtgaagatgcaaaagcagcactggatcgtgcagttgattatctgctgagcctgcaggatgaaaaaggtttttggaaaggtgaactggaaaccaacgttacca
ttgaagccgaagatctgctgctgcgtgaatttctgggtattcgtacaccggatattaccgcagaaaccgcacgttggattcgtgcaaaacagcgtagtgatggcacctgggcaaccttttatgat
ggtccgcctgatctgagcaccagcgttgaagcctatgttgcactgaaactggctggtgatgatccggcagcaccgcacatggaaaaagcagccgcatatattcgtggtgccggtggtgttga
acgtacccgtgtttttacccgtctgtggctggcactgtttggtctgtggccgtgggatgatctgccgacactgcctccggaaatgatttttctgccgagctggtttccgctgaacatttatgattggggtt
gttggccgcgccagaccgttgtgccgctgaccattgttagcgcactgcgtccggttcgtccgattccgctgagtattgatgaaattcgtacaggtgcaccgcctccgcctcgcgatccggcatgg
accattcgtggtttttttcagcgtctggatgatttactgcgtggttatcgtcgtgttgcagatcatggtccggcacgtctgtttcgtcgtctggcaatgcgtcgtgcagcagaatggattattgcacgtca
agaggcagatggtagctggggtggtattcagcctccatgggtttatagcctgattgcactgcatctgctgggttatccgctggatcatccggttctgcgtcgtggtctggatggtctgaatggttttac
cattcgcgaagaaacagcagatggtgcagttcgtcgcctggaaatgtgccagagtccggtttgggataccgcactggcagttacagcactgcgtgatgcaggtctgcctgccgatcatccgc
gtgttcaggcagcagcccgttggctggttggtgaagaggtgcgtgttgccggtgattgggcagtgcgtcgtccgggtctgcctcctggtggttgggcatttgaatttgccaatgataattacccgg

ataccgatgatacagcagaagttgttctggccctgcgtcgcgttcgtctggaagatgcagatcagcaggcgctggaagcagccgttcgtcgtgcaaccacctgggttattggtatgcagagca
ccgatggcggttggggtgcatttgatgcagataatacccgtgaactggtgctgcgtctgccgttttgtgattttggtgccgttattgatccgcctagcgcagatgttaccgcacatattgttgaaatgc
tggcagccctgggtatgcgtgatcatcctgccaccgttgcgggtgttcgttggctgctggcacatcaagaacctgatggtagttggtttggtcgttggggagcaaatcatatttatggtacgggtgc
agttgttccggcactgattgcagccggtgttagtccggatacaccgcctattcgtcgcgcaattcgctggctggaagaacatcagaatccggatggtggatggggtgaagatttacgtagctat
accgatcctgcactgtgggttggtcgtggtgttagcaccgcaagccagaccgcatgggcactgctggcattactggcagcgggtgaagaagcaagtccggcagttgatcgtggcgttcggtg
gctggttaccacacagcagcctgatggtggctgggatgagccgcattacaccggtacaggcttcccgggtgatttctatgttaattatcatctgtatcgcctggtgtttccgattagtgcactgggtc
gttatgttaatcgt

35. *Aci*SHC_R2.2

atgacccaggcaagcgttcgtgaagatgcaaaagcagcactggatcgtgcagttgattatctgctgagcctgcaggatgaaaaaggtttttggaaaggtgaactggaaaccaacgttacca
ttgaagccgaagatctgctgctgcgtgaatttctgggtattcgtacaccggatattaccgcagaaaccgcacgttggattcgtgcaaaacagcgtagtgatggcacctgggcaaccttttatgat
ggtccgcctgatctgagcaccagcgttgaagcctatgttgcactgaaactggctggtgatgatccggcagcaccgcacatggaaaaagcagccgcatatattcgtggtgccggtggtgttga
acgtacccgtgtttttacccgtctgtggctggcactgtttggtctgtggccgtgggatgatctgccgacactgcctccggaaatgatttttctgccgagctggtttccgctgaacatttatgattggggtt
gttggccgcgccagaccgttgtgccgctgaccattgttagcgcactgcgtccggttcgtccgattccgctgagtattgatgaaattcgtacaggtgcaccgcctccgcctcgcgatccggcatgg
accattcgtggtttttttcagcgtctggatgatttactgcgtggttatcgtcgtgttgcagatcatggtccggcacgtctgtttcgtcgtctggcaatgcgtcgtgcagcagaatggattattgcacgtca
agaggcagatggtagctggggtggtattcagtggccatgggtttatagcctgattgcactgcatctgctgggttatccgctggatcatccggttctgcgtcgtggtctggatggtctgaatggttttac
cattcgcgaagaaacagcagatggtgcagttcgtcgcctggaaatgtgccagagtccggtttgggataccgcactggcagttacagcactgcgtgatgcaggtctgcctgccgatcatccgc
gtgttcaggcagcagcccgttggctggttggtgaagaggtgcgtgttgccggtgattgggcagtgcgtcgtccgggtctgcctcctggtggttgggcatttgaatttgccaatgataattacccgg
ataccgatgatacagcagaagttgttctggccctgcgtcgcgttcgtctggaagatgcagatcagcaggcgctggaagcagccgttcgtcgtgcaaccacctgggttattggtatgcagagca
ccgatggcggttggggtgcatttgatgcagataatacccgtgaactggtgctgcgtctgccgttttgtgattttggtgccgttattgatccgcctagcgcagatgttaccgcacatattgttgaaatgc
tggcagccctgggtatgcgtgatcatcctgccaccgttgcgggtgttcgttggctgctggcacatcaagaacctgatggtagttggtttggtcgttggggagcaaatcatatttatggtacgggtgc
agttgttccggcactgattgcagccggtgttagtccggatacaccgcctattcgtcgcgcaattcgctggctggaagaacatcagaatccggatggtggatggggtgaagatttacgtagctat
accgatcctgcactgtgggttggtcgtggtgttagcaccgcaagccagaccgcatgggcactgctggcattactggcagcgggtgaagaagcaagtccggcagttgatcgtggcgttcggtg
gctggttaccacacagcagcctgatggtggctgggatgagccgcattacaccggtacaggcttcccgggtgatttctatcttaattatcatctgtatcgcctggtgtttccgattagtgcactgggtc
gttatgttaatcgt

36. *Aci*SHC_R2.3

atgacccaggcaagcgttcgtgaagatgcaaaagcagcactggatcgtgcagttgattatctgctgagcctgcaggatgaaaaaggtttttggaaaggtgaactggaaaccaacgttacca
ttgaagccgaagatctgctgctgcgtgaatttctgggtattcgtacaccggatattaccgcagaaaccgcacgttggattcgtgcaaaacagcgtagtgatggcacctgggcaaccttttatgat
ggtccgcctgatctgagcaccagcgttgaagcctatgttgcactgaaactggctggtgatgatccggcagcaccgcacatggaaaaagcagccgcatatattcgtggtgccggtggtgttga
acgtacccgtgtttttacccgtctgtggctggcactgtttggtctgtggccgtgggatgatctgccgacactgcctccggaaatgatttttctgccgagctggtttccgctgaacatttatgattggggtt
gttggccgcgccagaccgttgtgccgctgaccattgttagcgcactgcgtccggttcgtccgattccgctgagtattgatgaaattcgtacaggtgcaccgcctccgcctcgcgatccggcatgg
accattcgtggtttttttcagcgtctggatgatttactgcgtggttatcgtcgtgttgcagatcatggtccggcacgtctgtttcgtcgtctggcaatgcgtcgtgcagcagaatggattattgcacgtca
agaggcagatggtagctggggtggtattcagtggccatgggtttatagcctgattgcactgcatctgctgggttatccgctggatcatccggttctgcgtcgtggtctggatggtctgaatggttttac
cattcgcgaagaaacagcagatggtgcagttcgtcgcctggaactgtgccagagtccggtttgggataccgcactggcagttacagcactgcgtgatgcaggtctgcctgccgatcatccgc
gtgttcaggcagcagcccgttggctggttggtgaagaggtgcgtgttgccggtgattgggcagtgcgtcgtccgggtctgcctcctggtggttgggcatttgaatttgccaatgataattacccgg
ataccgatgatacagcagaagttgttctggccctgcgtcgcgttcgtctggaagatgcagatcagcaggcgctggaagcagccgttcgtcgtgcaaccacctgggttattggtatgcagagca
ccgatggcggttggggtgcatttgatgcagataatacccgtgaactggtgctgcgtctgccgttttgtgattttggtgccgttattgatccgcctagcgcagatgttaccgcacatattgttgaaatgc
tggcagccctgggtatgcgtgatcatcctgccaccgttgcgggtgttcgttggctgctggcacatcaagaacctgatggtagttggtttggtcgttggggagcaaatcatatttatggtacgggtgc
agttgttccggcactgattgcagccggtgttagtccggatacaccgcctattcgtcgcgcaattcgctggctggaagaacatcagaatccggatggtggatggggtgaagatttacgtagctat
accgatcctgcactgtgggttggtcgtggtgttagcaccgcaagccagaccgcatgggcactgctggcattactggcagcgggtgaagaagcaagtccggcagttgatcgtggcgttcggtg
gctggttaccacacagcagcctgatggtggctgggatgagccgcattacaccggtacaggcttcccgggtgatttctatgttaattatcatctgtatcgcctggtgtttccgattagtgcactgggtc
gttatgttaatcgt

G. References

[1] F. Sievers, A. Wilm, D. Dineen, T. J. Gibson, K. Karplus, W. Li, R. Lopez, H. McWilliam, M. Remmert, J. Söding, J. D. Thompson, D. G. Higgins, *Mol. Syst. Biol.* **2011**, *7*, DOI 10.1038/msb.2011.75.
[2] M. N. Price, P. S. Dehal, A. P. Arkin, *PLoS One* **2010**, *5*, DOI 10.1371/journal.pone.0009490.
[3] T. Frickey, E. Kannenberg, *Environ. Microbiol.* **2009**, *11*, 1224–1241.
[4] D. Ochs, C. Kaletta, K. D. Entian, A. Beck-Sickinger, K. Poralla, *J. Bacteriol.* **1992**, *174*, 298–302.
[5] G. Siedenburg, D. Jendrossek, M. Breuer, B. Juhl, J. Pleiss, M. Seitz, J. Klebensberger, B. Hauer, *Appl. Environ. Microbiol.* **2012**, *78*, 1055–1062.
[6] G. Siedenburg, M. Breuer, D. Jendrossek, *Appl. Microbiol. Biotechnol.* **2013**, *97*, 1571–1580.
[7] M. Perzl, P. Müller, K. Poralla, E. L. Kannenberg, *Microbiology* **1997**, *143*, 1235–1242.
[8] A. Tippelt, L. Jahnke, K. Poralla, *Biochim. Biophys. Acta - Lipids Lipid Metab.* **1998**, *1391*, 223–232.
[9] J. Shinozaki, M. Shibuya, K. Masuda, Y. Ebizuka, *FEBS Lett.* **2008**, *582*, 310–318.
[10] J. Shinozaki, M. Shibuya, Y. Takahata, K. Masuda, Y. Ebizuka, *ChemBioChem* **2010**, *11*, 426–433.
[11] T. Sato, S. Yoshida, H. Hoshino, M. Tanno, M. Nakajima, T. Hoshino, *J. Am. Chem. Soc.* **2011**, *133*, 9734–9737.

[12] T. Sato, H. Hoshino, S. Yoshida, M. Nakajima, T. Hoshino, *J. Am. Chem. Soc.* **2011**, *133*, 17540–17543.
[13] A. Waterhouse, M. Bertoni, S. Bienert, G. Studer, G. Tauriello, R. Gumienny, F. T. Heer, T. A. P. De Beer, C. Rempfer, L. Bordoli, R. Lepore, T. Schwede, *Nucleic Acids Res.* **2018**, *46*, W296–W303.
[14] O. Trott, A. J. Olson, *J. Comput. Chem.* **2009**, *31*, 455–461.
[15] E. Eichhorn, E. Locher, S. Guillemer, D. Wahler, L. Fourage, B. Schilling, *Adv. Synth. Catal.* **2018**, *360*, 2339–2351.
[16] B. A. Baker, Ž. V. Bošković, B. H. Lipshutz, *Org. Lett.* **2008**, *10*, 289–292.
[17] E. Brenna, C. Fuganti, S. Serra, P. Kraft, *European J. Org. Chem.* **2002**, 967–978.
[18] C. Fuganti, S. Serra, A. Zenoni, *Helv. Chim. Acta* **2000**, *83*, 2761–2768.
[19] S. Racolta, P. B. Juhl, D. Sirim, J. Pleiss, *Proteins Struct. Funct. Bioinforma.* **2012**, *80*, 2009–2019.
[20] S. C. Hammer, P. O. Syrén, B. Hauer, *ChemistrySelect* **2016**, *1*, 3589–3593.
[21] A. Schneider, P. Jegl, B. Hauer, *Angew. Chem. Int. Ed.* **2021**, 60, 13251–13256; *Angew. Chem.* **2021**, 133, 13359–13365.
